# A palladium-catalyzed synthesis of (hetero)aryl-substituted imidazoles from aryl halides, imines and carbon monoxide†
†Electronic supplementary information (ESI) available: Experimental procedures, characterization data, and NMR spectra for compounds. See DOI: 10.1039/c6sc04371b



**DOI:** 10.1039/c6sc04371b

**Published:** 2016-11-03

**Authors:** Jevgenijs Tjutrins, Bruce A. Arndtsen

**Affiliations:** a Department of Chemistry , McGill University , 801 Sherbrooke St. W. , Montreal , QC , Canada H3A 0B8 . Email: bruce.arndtsen@mcgill.ca ; Fax: +1-514-398-3797 ; Tel: +1-514-398-6999

## Abstract

We describe here a tandem catalytic route to prepare imidazoles in a single operation from aryl iodides, imines and CO. The reaction involves a catalytic carbonylation of aryl halides with imines to form 1,3-dipoles, which undergo spontaneous 1,3-dipolar cycloaddition. Overall, this offers an alternative to coupling reactions to construct the (hetero)aryl-imidazole motif, where variation of the building blocks can allow the synthesis of broad families of imidazoles with independent control of all substituents.

## Abstract 2

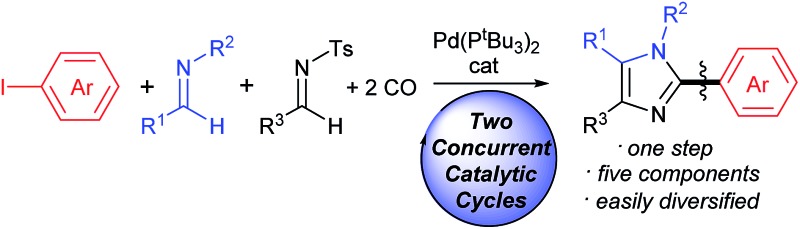
We describe here a tandem catalytic route to prepare imidazoles in a single operation from aryl iodides, imines and CO.

## Introduction

**Fig. 1:**
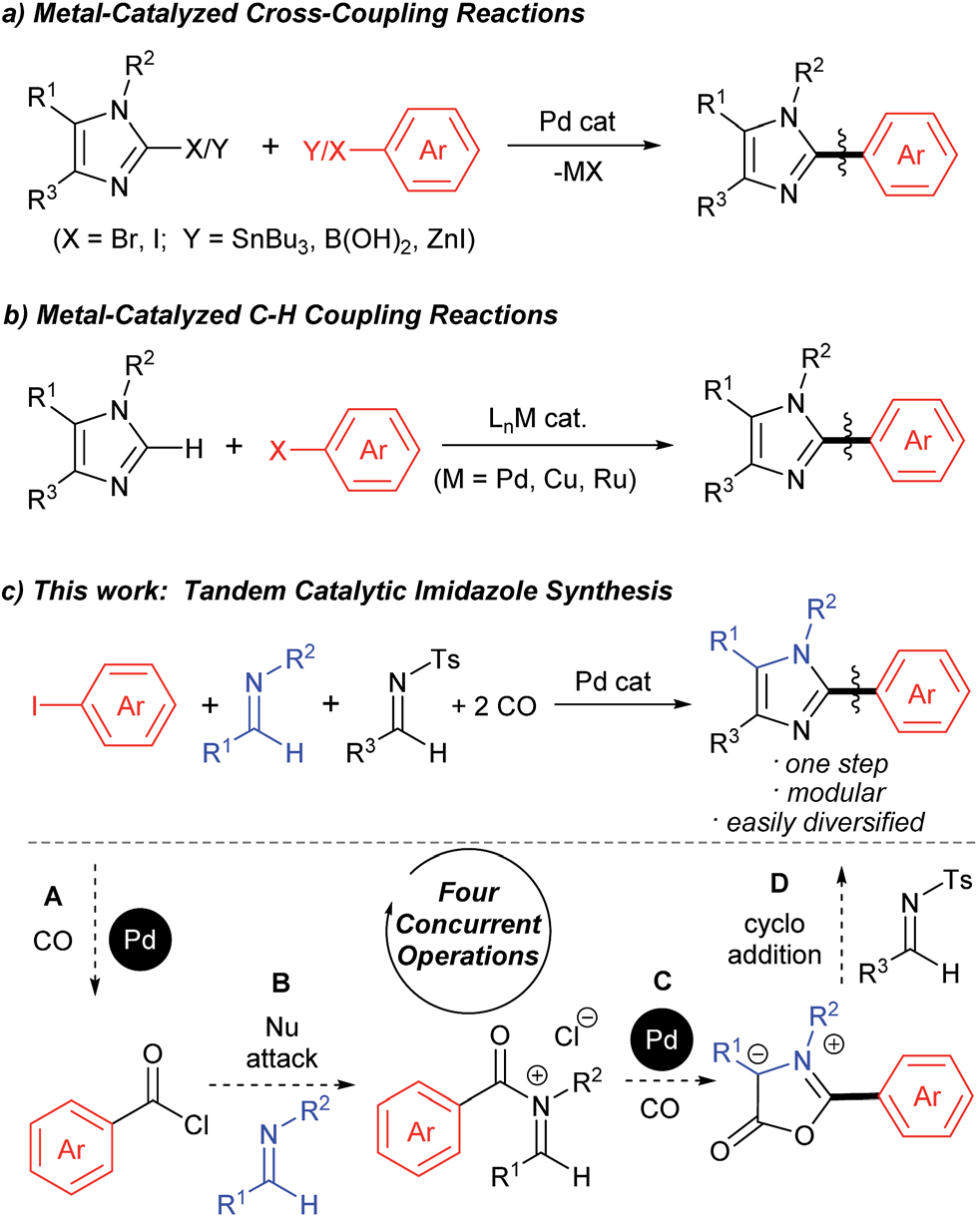
Transition metal-catalyzed approaches to aryl-substituted imidazoles.

The aryl-(hetero)aryl motif is among the most common structural motifs found in pharmaceutical design.^1^ An important example of these are aryl- and heteroaryl-substituted imidazoles, which are key units in a diverse range of anti-inflammatory^2^ and other pharmaceutically relevant agents,^3^ as well as electronic materials,^4^ polymers,^5^ metal coordinating ligands,^6^ and other areas of application.^7^ Traditional approaches to assemble aryl-substituted imidazoles involve the cyclization of pre-synthesized diamines with electrophiles.^8^ While effective, these require the initial multistep synthesis of the substituted precursors, and can suffer from poor regioselectivity. In this regard, the rapid rise in the use of cross-coupling reactions with heterocycles has had a tremendous impact. These commonly exploit the reaction of halogenated imidazoles with organometallic reagents (Fig. 1a).^9^ More recently, efforts by a number of research groups have demonstrated that cross-coupling can be replaced with even more efficient metal catalyzed C–H bond functionalization (Fig. 1b).^10^ The latter obviate the need to pre-activate the imidazole unit and the use metallated coupling partners, and instead can employ the broad range of commercially available (hetero)aryl halides.

Despite the many attractive features of coupling reactions, there remain drawbacks to their use in assembling substituted imidazoles. Perhaps most importantly, intrinsic to this chemistry is the need for the pre-formed, substituted imidazole for use in bond formation. These must be prepared, often by classical cyclization chemistry, and can make the systematic tuning of the imidazole core an involved process. Other routes to imidazoles have been developed,^11^ including our own Pd-catalyzed synthesis with *N*-acyl iminium salts,^11*b*^ but these also often involve reactive and/or synthetic reagents. In principle, a more flexible method to generate to these products would be to assemble the aryl-imidazole bond at the same time as the heterocycle. A potential approach to such a synthesis is to exploit tandem catalysis. Tandem catalytic reactions have seen growing interest due to their ability to generate multiple bonds through a series of spontaneous catalytic operations.^12^ We questioned if these features might be applied to design a synthesis of imidazoles from aryl halides, *via* the reaction illustrated in Fig. 1c. We have recently shown that aryl halides can undergo palladium catalyzed carbonylation into reactive acid chlorides.^13^ Performing this reaction in the presence of an imine can initiate a second carbonylation and the overall generation of 1,3-dipolar münchnones.^14^ An interesting facet of the latter reaction is its ability to generate a reactive, 1,3-dipole intermediate from an aryl halide, which has the ability to undergo cycloaddition.^15^ The overall sequence in Fig. 1c would offer what is to our knowledge a rare example of a tandem catalytic reaction involving five separate reagents.^16^ While each of these steps has precedent, the complete transformation requires the performance of two separate palladium catalyzed carbonylation reactions (**A** and **C**), together with nucleophilic attack (**B**) and cycloaddition (**D**) with perfect selectivity. Similarly, the catalyst, multiple reagents, reactive intermediates, coordinating product and two separate imines must all be compatible and react in sequence in a single reaction mixture.

We describe below our efforts towards this goal. This has led to a novel route to assemble imidazoles from combinations of substrates that are available, easily diversified and stable (aryl halides, two imines and CO). The catalytic reaction proceeds with high chemoselectivity, and opens an alternative to coupling reactions to prepare aryl-imidazoles from aryl halides, where variation of the substrates can allow the formation of broad families of products.

## Results and discussion

Our initial studies involved the Pd(P^*t*^Bu_3_)_2_ catalyzed coupling of aryl iodide with the two imines shown in Table 1, based upon our previous observations of the activity of these catalysts in aryl iodide carbonylation. This leads to minimal product after several days at elevated temperatures (entry 1). Modulation of the reaction conditions did not favor the reaction, nor did changing the palladium coordinating ligand (entries 2–5, see Table S1† for full catalyst development). We have previously noted that the presence of chloride can dramatically accelerate carbonylations with aryl iodides by allowing the *in situ* build-up of acid chlorides.^13^ Similarly, the addition of Bu_4_NCl, or the even more straightforward use of [Pd(allyl)Cl]_2_ as the catalyst and chloride sources allowed the formation of imidazole **1a** in moderate yield (entries 6, 7). No subsequent optimization improved this yield.

**Table 1:** Catalyst development for the generation of aryl-imidazoles^*a*^

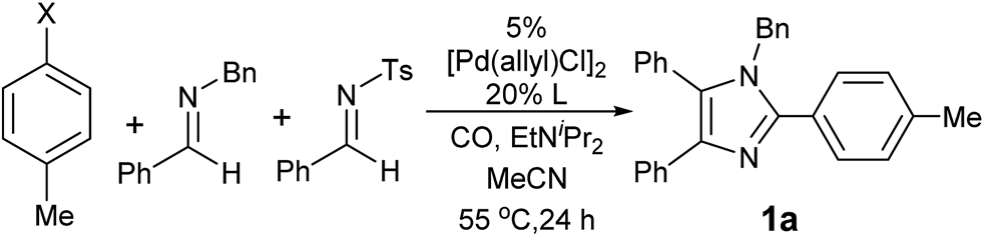					
Entry	L	**1a** ^*b*^ (%)	Entry	L	**1a** ^*b*^ (%)
**X = I**					
1	Pd(P^*t*^Bu_3_)_2_	4	6^*b*^	Pd(P^*t*^Bu_3_)_2_	43
2	P(*o*-tolyl)_3_	5	7	P^*t*^Bu_3_	42
3	DPPE	—	8^*c*^	P^*t*^Bu_3_	—
			9^*d*^	P^*t*^Bu_3_	30
4	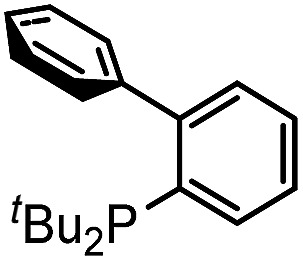	7	10^*e*^	P^*t*^Bu_3_	12
5	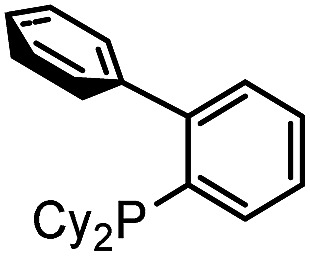	7	11^*f*^	P^*t*^Bu_3_	77 (75)^*g*^

**X = Br**					
12^*b*^ ^,^ ^*f*^	Pd(P^*t*^Bu_3_)_2_	—	13^*b*^ ^,^ ^*f*^ ^,^ ^*h*^	P^*t*^Bu_3_	73 (65)^*i*^

^*a*^ *p*-Iodotoluene (109 mg, 0.50 mmol), PhC = NBn (20 mg, 0.10 mmol), PhC = NTs (31 mg, 0.12 mmol), [Pd(allyl)Cl]_2_ (0.005 mmol), L (0.02 mmol) EtN^*i*^Pr_2_ (0.3 mmol), 0.7 mL CD_3_CN, 4 atm CO.

^*b*^10% Pd(P^*t*^Bu_3_)_2_, 0.1 mmol Bu_4_NCl.

^*c*^PhC = NMs.

^*d*^PhC = NSO_2_C_6_H_4_Cl.

^*e*^PhC = NNs.

^*f*^ *p*-Halotoluene (0.3 mmol), PhC = NBn (39 mg, 0.2 mmol).

^*g*^Isolated.

^*h*^95 °C, 25 atm CO, 20% P^*t*^Bu_3_.

^*i*^4 atm CO.

Closer examination of the reaction mixture shows that the *N*-benzyl imine is fully consumed, suggesting that the initial catalytic carbonylation cycle may be effective, but subsequent steps are inhibited. We suspected that this may arise from the formation of the sulfinate byproduct of cycloaddition. Control experiments show that the addition of stoichiometric sodium sulfinate completely blocks catalysis.^17^ Attempts to improve the reaction yield by varying this leaving group did not increase the yield of imidazole (entries 8–10), nor did the addition of potential sulfinate trapping agents.^18^ However, the simple change of making the *N*-tosyl imine the limiting reagent dramatically improved catalysis (entry 11). From a synthetic perspective this proved useful, as *N*-tosyl imines are the most valuable building blocks in the reaction. Under these conditions, imidazole **1a** is formed in high yield and with the exclusive incorporation of two separate imines (*vide infra*).

In considering the substrates employed in this reaction, we next questioned if even more broadly available and inexpensive aryl bromides might also be employed in the catalytic sequence. A challenge in the use of aryl bromides is the required use of pressing conditions required to activate the Ar–Br bond, which must be compatible with the sensitive iminium salt or münchnone intermediates generated in this reaction. However, after probing various reaction conditions (see Table S2† for details), we have found that the rapid trapping of the intermediates to imidazoles (*vide infra*) can allow this tandem catalytic sequence to be performed with aryl bromides, and affords imidazole with similar efficiency (entry 13).

A feature of this catalytic synthesis is its ability to generate substituted imidazoles from combinations of building blocks that can easily diversified. This can allow for the modular assembly of a diverse range of these products (Table 2). For example, a number of simple imines can be incorporated into the reaction, including those with phenyl substituents on the imine carbon, electron deficient aromatics (**1b**, **f**) those with donor substituents (**1c**, **d**, **h**, **k**) as well as heterocyclic imines (**1m**). Each of these form imidazoles in good yields. Similarly, those with various *N*-alkyl, -benzyl and -aryl substituents are viable partners, as are those with heteroaromatic units. The *N*-tosyl substituted imine can be even more widely diversified as a tool to modulate the 4-imidazole position. Examples of these latter can involve electron rich or electron deficient aromatics (**1n–t**) or naphthyl substituents (**1u**). α,β-Unsaturated *N*-tosyl imines are also viable substrates, as are those with heteroaromatic (thiophene, furan, pyridine) units (**1w–z**). The reaction also tolerates the use of enolizable, *C*-alkyl substituted imines (**1aa**).

**Table 2:** Scope of imine coupling partners^*a*^

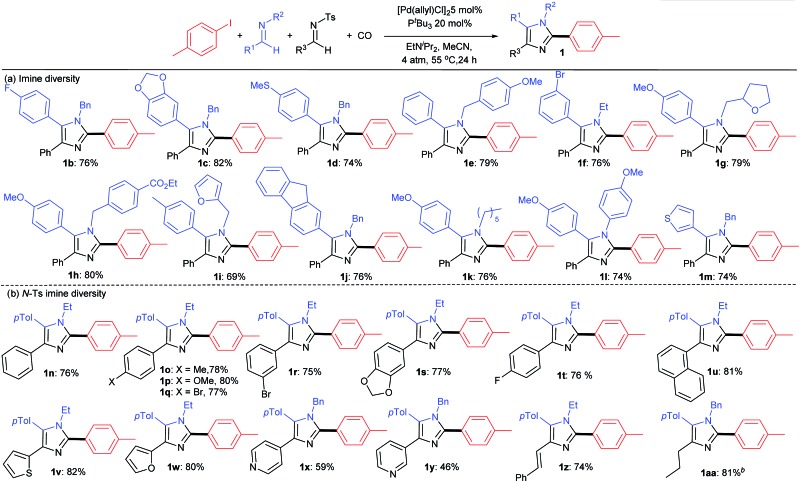

^*a*^Aryl iodide (327 mg, 1.50 mmol), *N*-Ts imine (0.50 mmol), [Pd(allyl)Cl]_2_ (9 mg, 0.025 mmol), PtBu_3_ (20 mg, 0.10 mmol), EtNiPr_2_ (194 mg, 1.50 mmol), MeCN (2.0 mL), 4 atm CO, 55 °C, 24 h.

^*b*^ *N*-Ts imine added after münchnone formation.

As shown in Table 3, the aryl iodide coupling partner can also be modulated in this transformation. As representative examples, various *para*-, *meta*-, and even sterically encumbered *ortho*-substituents can be incorporated onto the aryl-iodide or -bromide (**2a–n**), as can those with palladium reactive functionalities (**2d–i**). Electron rich aryl halides generally lead to slightly higher yields (**2c**, **n**, **o**), but electron withdrawing substituents are also viable reagents. This chemistry can be extended to the use of heteroaryl iodides, such as those with thiophene and indole units. When combined with the diversity of imine(s) that can be employed, this transformation provides what is to our knowledge a novel method to prepare imidazoles from aryl halides, where every substituent can be varied at the same time as the imidazole is generated.

**Table 3:** Scope of aryl halide coupling partners^*a*^

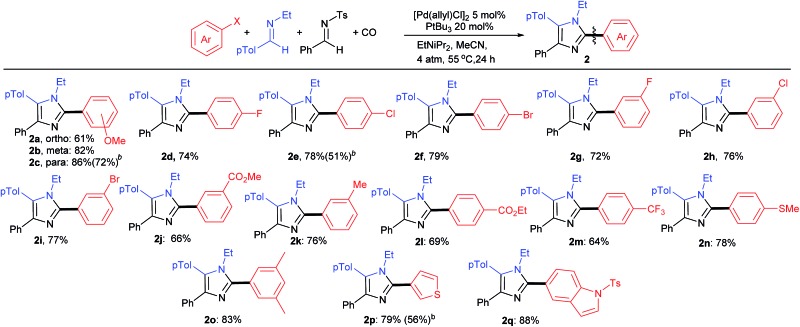

^*a*^Reaction conditions of Table 2.

^*b*^Reaction performed with aryl bromide.

**Fig. 2:**
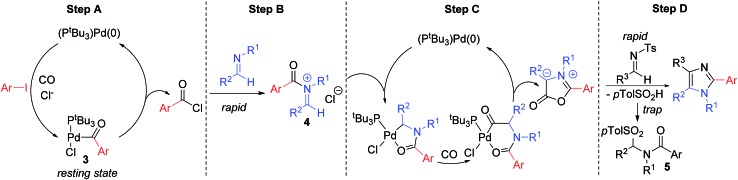
Proposed catalytic cascade to generate (hetero)aryl-substituted imidazoles.

We have performed preliminary studies to probe the mechanism of this catalytic reaction. Monitoring the reaction by ^1^H and ^31^P NMR analysis shows the build-up of palladium–aroyl complex **3** (Fig. 2) as the catalyst resting state, suggesting the elimination of acid chloride (step **A**) is rate determining in catalysis.^19^ This data is consistent with the results in Table 1, where the sterically encumbered P^*t*^Bu_3_ ligand and a chloride source are both required for efficient catalysis, as they can favor acid chloride reductive elimination.^13^ Once acid chloride is formed, it presumably reacts rapidly with imine to form an *N*-acyl iminium salt. *In situ* ^1^H NMR analysis shows no evidence for iminium salt **4**, and likely reflects its more rapid oxidative addition to palladium than aryl iodide for subsequent cyclocarbonylation to münchnone (step **C**). The final cycloaddition of *N*-tosyl imine leads to the liberation of sulfinate. ^1^H NMR analysis shows that this sulfinate is rapidly trapped with iminium salt to form PhCON(Bn)CH(*p*-Tol)Ts (**5**), and does not therefore block either of the two palladium catalyzed carbonylation cycles. The high imine selectivity in this system presumably arises from their different electronic characteristics, wherein the poorly nucleophilic *N*-tosyl imine cannot react with acid chloride to form iminium salt. Conversely, once münchnone is generated, it undergoes selective 1,3-dipolar cycloaddition with the more electron deficient imine and aromatization.

**Fig. 3:**
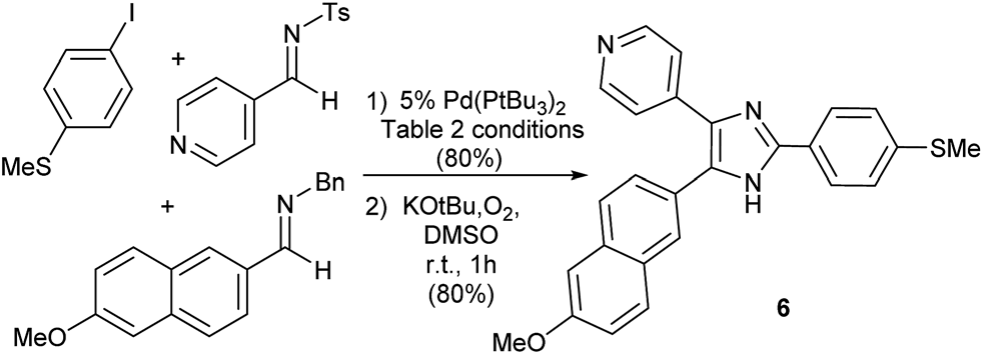
Aryl iodide carbonylative synthesis of imidazole **6**.

Finally, as an illustration of the potential utility of this transformation, we have probed its use in more targeted synthesis. Imidazole **6** (Fig. 3) has attracted attention as a potent Tie2 kinase inhibitor.^20^ While **6** is typically prepared *via* cyclization of preformed polysubstituted ketones, this palladium catalyzed platform can allow access to the imidazole core of **6** directly from combinations of two imines, CO and aryl iodide. The modularity of this catalytic reaction should in principle allow facile access to variants of this product, by systematic tuning of either the imine or aryl halide building blocks.

## Conclusions

In conclusion, we have described a new palladium-based tandem catalytic transformation that can allow the generation of polysubstituted imidazoles from combinations of imines, aryl halides and carbon monoxide. This provides an alternative and modular route to prepare (hetero)aryl-substituted imidazoles from aryl halides. The reaction proceeds with high selectivity, and allows the build-up of these products in one operation, from available substrates, and with independent control of all substituents.
