# Supplementary material for: A palladium-catalyzed synthesis of (hetero)aryl-substituted imidazoles from aryl halides, imines and carbon monoxide
Source: Chem Sci. 2016 Nov 3;8(2):1002–7. doi: 10.1039/c6sc04371b (PMC5354067; doi:10.1039/c6sc04371b)

Electronic Supplementary Material (ESI) for Chemical Science.
This journal is © The Royal Society of Chemistry 2016

A Palladium-Catalyzed Synthesis of (Hetero)Aryl-Substituted Imidazoles from Imines, Aryl Halides and Carbon Monoxide

Jevgenijs Tjutrins and Bruce A. Arndtsen*

Department of Chemistry, McGill University, 801 Sherbrooke Street West, Montreal, Quebec, H3A 0B8, Canada

Supporting Information

Table of Contents

**I.**	**General Considerations**	S2
**II.**	**Synthetic Procedures**	S2
**III.**	**Spectroscopic Data**	S6
**IV.**	**References**	S19
**V.**	**NMR Spectra**	S20

I. General Considerations

All reactions were carried out under an inert atmosphere in a glovebox or using standard Schlenk techniques, unless otherwise indicated. Research grade carbon monoxide (99.99%) was used as received. All solvents were dried using a solvent purification system and stored in glovebox over activated 4 Å molecular sieves. Deuterated solvents were dried over CaH_2, vacuum transferred and stored over 4 Å molecular sieves. N-alkyl, N-aryl imines, and N-tosyl imines were prepared according to literature procedures.[1-4] All other reagents were purchased from commercial suppliers and used as received. All ^{1}H and ^{13}C NMR spectra were acquired on 400 and 500 MHz spectrometers. High resolution mass spectra were obtained using a quadrupole-time of flight and an Orbitrap detector by direct infusion in positive ESI mode.

II. Synthetic Procedures

Typical Procedure for Catalyst Screening (Table 1)

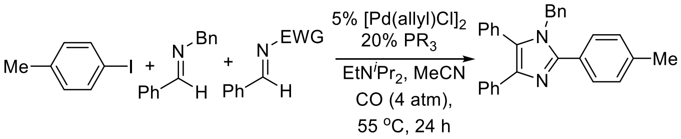

In a glovebox, 4-iodotoluene (109 mg, 0.50 mmol), PhC=NBn (20 mg, 0.10 mmol), PhC=NTs (31 mg, 0.12 mmol), $EtN^{i}Pr_2$ (40 mg, 0.3 mmol), $[Pd(allyl)Cl]_2$ (2 mg, 0.005 mmol), $P^{t}Bu_3$ (4 mg, 0.02 mmol), and benzyl benzoate internal standard were dissolved in CD_3CN (0.7 mL) and added to a J-Young NMR tube. The tube was removed from the glovebox, frozen in liquid nitrogen, the headspace evacuated, and 4 atm carbon monoxide was condensed into the NMR tube. The reaction was heated at 55 ^{o}C and monitored by ^{1}H NMR spectroscopy. The yield of **1a** (43%) was determined by ^{1}H NMR analysis relative to the internal standard. Also, monitoring the reaction during the course of catalysis by ^{1}H and ^{31}P NMR shows the generation of the palladium complex $(^{t}Bu_3P)Pd(CO$*p*Tol)Cl (^{31}P NMR: 73.1 ppm) as the major observable phosphine-containing intermediate, and the formation of (N-benzylbenzamido)(phenyl)methyl 4-methylbenzenesulfinate **5** in 48% yield at the end of catalysis. The results of screening of other ligands, reagents and additives to the reaction are shown in Table S1 below.

Table S1. Catalyst Development for Generation of Aryl-Imidazoles from Aryl Iodides

Entry	PR_3	EWG	Yield, %	Entry	PR_3	EWG	Additive	Yield, %
1	$Pd(P^tBu_3)_2$	Ts	4	10	$Pd(P^tBu_3)_2$	Ts	Bu_4NCl	43
2	PPh_3	Ts	0	11	P^tBu_3	Ts		42
3	PCy_3	Ts	0	12	P^tBu_3	$SO_2C_6H_4Cl$		30
4	P(*o*-Tol)$_3$	Ts	5	13	P^tBu_3	NS		12
5	DPPE	Ts	0	14	P^tBu_3	Ms		0
6	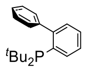	Ts	7	15	P^tBu_3	Ts		0
7	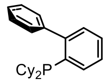	Ts	7	16	P^tBu_3	Ts	*n*-propyl-Br	0
8	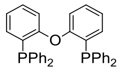	Ts	0	17	P^tBu_3	Ts	TMSCl	32
9	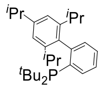	Ts	8	18	P^tBu_3	Ts		77(75)

Typical Procedure for Catalyst Screening with Aryl Bromides (Table 1)

In a glovebox, 4-bromotoluene (68 mg, 0.40 mmol), PhC=NBn (40 mg, 0.20 mmol), PhC=NTs (26 mg, 0.12 mmol), EtN^iPr_2 (40 mg, 0.3 mmol), $Pd(P^tBu_3)_2$ (5 mg, 0.01 mmol), Bu_4NCl (28 mg, 0.1 mmol), P^tBu_3 (4 mg, 0.02 mmol), and benzyl benzoate internal standard were dissolved in MeCN (1 mL) in a vial with a loosely capped screw cap and added to a high pressure reactor. The reactor was removed from the glovebox, and was pressurized with 25 atm carbon monoxide. The reaction was heated at 95 ^oC for 24 h. The reaction was then cooled to room temperature, carbon monoxide was removed and all volatiles were evaporated in vacuo. The yield of **1a** (73%) was determined by ^1H NMR analysis relative to the internal standard. The results of screening other conditions and additives is provided in Table S2.

Table S2. Catalyst Development for Generation of Aryl-Imidazoles from Aryl Bromides

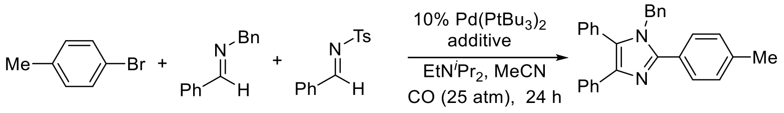

Entry	Temp., ^oC	Additive	Yield, %
1[a]	85	-	31%
2	75	1.0 eq. Bu_4NCl	35%
3	85	1.0 eq. Bu_4NCl	36%
4	95	1.0 eq. Bu_4NCl	39%

5	95	0.1 eq. Bu_4NCl	31%
6	95	0.1 eq. Bu_4NCl<br>20 mol% P^tBu_3	40%
7^b	95	1.0 eq. Bu_4NCl,<br>20 mol% P^tBu_3	72%
8^c	95	1.0 eq. Bu_4NCl,<br>20 mol% P^tBu_3	65%
9	95	1.0 eq. Bu_4NCl,<br>20 mol% P^tBu_3	73%

[a] $[Pd(allyl)Cl]_2/P^tBu_3$ used as catalyst. [b] 10 eq. of *p*-bromotoluene. [c] CO pressure 4 atm.

Typical Synthesis of Imidazoles (Tables 2 and 3)

In a glovebox, 4-iodotoluene (327 mg, 1.50 mmol), PhC=NBn (98 mg, 1.00 mmol), Ph=NTs (130 mg, 0.50 mmol), NEt^iPr_2 (194 mg, 1.50 mmol), $[Pd(allyl)Cl]_2$ (9 mg, 0.025 mmol), P^tBu_3 (20 mg, 0.10 mmol) were combined in acetonitrile (2 mL) and added to a 50 mL sealable pressure vessel. The vessel was closed, removed from the glovebox, and pressurized with 4 atm of carbon monoxide. The reaction was heated at 55 °C for 24 h. After the reaction was cooled to room temperature, all volatiles were removed in vacuo. The crude product was purified by column chromatography (silica gel, gradient hexane / ethyl acetate 4% to 20% with 1 % Et_3N additive) affording pure imidazole **1a** as a pale yellow solid in 75% yield (151 mg).

For the synthesis of **1aa**, the reaction was performed as above without the initial addition of the *N*-tosyl imine. After the reaction was complete, the CO was removed, the pressure vessel was brought back into glovebox and freshly synthesized (propyl)HC=NTs[3] (225 mg, 1.00 mmol) was added to the reaction mixture. The reaction heated at 55 °C for 5 h, then cooled to room temperature, and all volatiles were removed in vacuo. The crude product was purified by column chromatography (silica gel, gradient hexane / ethyl acetate 4% to 20% with 1 % Et_3N additive) affording imidazole **1aa** as a colorless oil in 81% yield (155 mg).

Procedure for the Synthesis of Imidazoles from Aryl Bromides

In a glovebox, 4-bromotoluene (342 mg, 2.0 mmol), Ph=NBn (195 mg, 1.0 mmol), PhC=NTs (130 mg, 0.5 mmol), EtN^iPr_2 (193 mg, 1.5 mmol), $[Pd(P^tBu_3)_2$ (12 mg, 0.025 mmol), P^tBu_3 (10 mg, 0.1 mmol), and benzyl benzoate internal standard were combined in MeCN (5.0 mL) and

added to a high pressure reactor. The reactor was removed from the glovebox, and was pressurized with 25 atm carbon monoxide. The reaction was heated at 95 ^{o}C for 24 h. Afterwards the reaction was cooled to room temperature, carbon monoxide was removed and all volatiles were evaporated in vacuo. The crude product was purified by column chromatography (silica gel, gradient hexane / ethyl acetate 4% to 20% with 1 % Et_3N additive) affording pure imidazole **1a** as a pale yellow solid in 70% yield (140.0 mg).

Procedure for the Synthesis of 4-(5-(6-methoxynaphthalen-2-yl)-2-(4-(methylthio)phenyl)-1H-imidazol-4-yl)pyridine **6**.

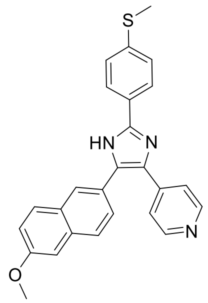

In a glovebox, 4-iodothioanisole (1000 mg, 4.0 mmol), N-benzyl-1-(6-methoxynaphthalen-2-yl)methanimine (275 mg, 1.00 mmol), $NEt^{i}Pr_2$ (194 mg, 1.50 mmol), $Pd(P^{t}Bu_3)_2$ (12 mg, 0.05 mmol), and Bu_4NCl (278 mg, 1.0 mmol) were combined in acetonitrile (15 mL) and added to a 50 mL sealable pressure vessel. The vessel was closed, removed from the glovebox, and pressurized with 4 atm of carbon monoxide. The reaction was heated at 40°C for 24 h. After the reaction was cooled to room temperature, the CO was removed on a Schlenk line, and the pressure vessel was brought into a glovebox. 4-Methyl-N-(pyridin-4-ylmethylene)benzenesulfonamide (260 mg, 1.00 mmol) was added to the crude mixture and stirred for 4 h at 40°C. Afterwards all the volatiles were removed in vacuo, and the crude mixture was purified by column chromatography (silica gel, gradient hexane / ethyl acetate 4% to 85% with 1 % Et_3N additive) affording the imidazole as a pale yellow solid in 80% yield (413 mg).

Based on literature procedure,[5] this product, [4-(1-benzyl-5-(6-methoxynaphthalen-2-yl)-2-(4-(methylthio)phenyl)-1H-imidazol-4-yl)pyridine], (102 mg, 0.2 mmol), DMSO (156 mg, 2.0 mmol) and potassium tert-butoxide (1.4 mL, 1 mol/L in THF) were combined in a flame-dried

flask. The reaction was stirred and oxygen was bubbled through the solution for 1h. Upon completion, the reaction mixture was quenched with aqueous sodium carbonate solution. The THF was removed in vacuo, and the product was extracted three times with EtOAc. The organics were combined, dried and concentrated in vacuo. The crude mixture was purified by column chromatography (silica gel, gradient hexane / ethyl acetate 30% to 85%) affording the imidazole. An ethyl acetate solution of the product was washed with a saturated solution of Na_2CO_3, and then dried in vacuo to yield **6** as a pale brown solid in 80% yield (67 mg).

<u>Reaction of N-Acyliminium Salt with Sodium *p*-Toluenesulfinate</u>

Ph(H)C=NBn (20 mg, 0.10 mmol), benzoyl chloride (21 mg, 0.15 mmol), and benzyl benzoate internal standard were dissolved in 0.7 mL of CD_3CN and stirred at room temperature for 10 min. To this solution was added sodium p-toluenesulfinate (27 mg, 0.15 mmol), and the obtained mixture was heated at 55 ^oC and reaction was monitored by in situ ^1H NMR. ^1H NMR shows generation of (N-benzylbenzamido)(phenyl)methyl 4-methylbenzenesulfinate in 52% yield after 18 h,[6] and N-benzylbenzamide in 6% yield compared to internal standard.

III. Spectroscopic Data

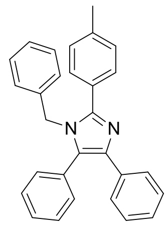

1-Benzyl-4,5-diphenyl-2-(p-tolyl)-1H-imidazole 1a. Pale yellow solid, 151 mg, 75% yield. ^1H NMR (500 MHz, $CDCl_3$) δ 7.62 (d, *J* = 7.2 Hz, 2H), 7.59 (d, *J* = 8.1 Hz, 2H), 7.34 (m, 3H), 7.27 – 7.21 (m, 9H), 7.17 (t, *J* = 7.3 Hz, 1H), 6.88 – 6.82 (m, 2H), 5.13 (s, 2H), 2.40 (s, 3H). ^{13}C NMR (126 MHz, $CDCl_3$) δ 148.2, 138.8, 138.0, 137.7, 134.6, 131.15, 131.10, 129.9, 129.3, 129.0, 128.8*, 128.6, 128.08, 128.05, 127.3, 126.8, 126.3, 126.0, 48.3, 21.4. HRMS. Calculated for $C_{29}H_{25}N_2$ $(M+H^+)$: 401.2012, found: 401.1994.

*Selective HSQC NMR experiment indicates that signal at δ 128.8 ppm corresponds to two carbons.

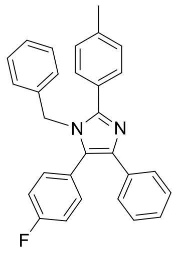

1-Benzyl-5-(4-fluorophenyl)-4-phenyl-2-(p-tolyl)-1H-imidazole 1b. White solid, 159 mg, 76% yield. ^1H NMR (500 MHz, $CDCl_3$) δ 7.59 (d, *J* = 8.2 Hz,

4H), 7.27 – 7.21 (m, 7H), 7.20 – 7.16 (m, 3H), 7.02 (t, *J* = 8.7 Hz, 2H), 6.84 (dd, *J* = 7.3, 1.9 Hz, 2H), 5.11 (s, 2H), 2.40 (s, 3H). ^{13}C NMR (126 MHz, $CDCl_3$) δ 162.84 (d, *J* = 248.8 Hz), 148.4, 139.0, 138.3, 137.5, 134.4, 133.0 (d, *J* = 8.3 Hz), 129.3, 129.1 (d, *J* = 59.9 Hz), 128.9, 128.7, 128.1, 127.9, 127.5, 127.13 (d, *J* = 3.4 Hz), 126.8, 126.5, 126.0, 115.9 (d, *J* = 21.5 Hz), 48.3, 21.4. HRMS. Calculated for $C_{29}H_{24}N_2F$ ($M+H^+$): 419.1918, found: 419.1918.

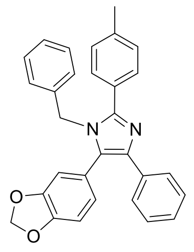

5-(Benzo[d][1,3]dioxol-5-yl)-1-benzyl-4-phenyl-2-(p-tolyl)-1H-imidazole 1c. Pale yellow solid, 182 mg, 82% yield. ^{1}H NMR (500 MHz, $CDCl_3$) δ 7.66 (d, *J* = 7.5 Hz, 2H), 7.57 (d, *J* = 7.8 Hz, 2H), 7.22 (m, 8H), 6.87 (d, *J* = 6.6 Hz, 2H), 6.78 (d, *J* = 7.9 Hz, 1H), 6.71 (d, *J* = 7.8 Hz, 1H), 6.67 (s, 1H), 5.99 (s, 2H), 5.13 (s, 2H), 2.39 (s, 3H). ^{13}C NMR (126 MHz, $CDCl_3$) δ 148.0, 147.9, 147.9, 138.8, 138.0, 137.7, 134.5, 129.4, 129.3, 128.9, 128.6, 128.1, 128.0, 127.4, 126.7, 126.3, 126.0, 125.1, 124.4, 111.3, 108.7, 101.3, 48.2, 21.4. HRMS. Calculated for $C_{30}H_{25}N_2O_2$ ($M+H^+$): 445.1911, found: 445.1913.

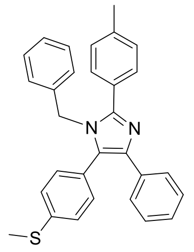

1-Benzyl-5-(4-(methylthio)phenyl)-4-phenyl-2-(p-tolyl)-1H-imidazole 1d. Colorless oil 165 mg, 74% yield. ^{1}H NMR (500 MHz, $CDCl_3$) δ 7.63 (d, *J* = 7.2 Hz, 2H), 7.58 (d, *J* = 8.0 Hz, 2H), 7.27 – 7.21 (m, 7H), 7.20 – 7.17 (m, 3H), 7.12 (d, *J* = 8.4 Hz, 2H), 6.84 (d, *J* = 7.8 Hz, 2H), 5.13 (s, 2H), 2.51 (s, 3H), 2.39 (s, 3H). ^{13}C NMR (126 MHz, $CDCl_3$) δ 148.0, 139.7, 137.3, 131.9, 131.3, 129.4, 129.1, 128.7, 128.2, 128.2, 127.7, 127.5, 127.2, 127.1, 127.0, 126.7, 126.2, 126.0, 124.0, 48.4, 21.4, 15.2. HRMS. Calculated for $C_{30}H_{27}N_2S$ ($M+H^+$): 447.1889, found: 447.1893.

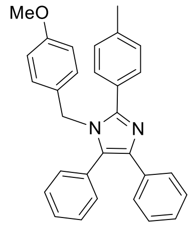

1-(4-Methoxybenzyl)-4,5-diphenyl-2-(p-tolyl)-1H-imidazole 1e. Pale yellow solid, 170 mg, 79% yield. ^{1}H NMR (400 MHz, $CDCl_3$) δ 7.61 (t, *J* = 7.7 Hz, 4H), 7.40 – 7.32 (m, 3H), 7.30 – 7.21 (m, 6H), 7.17 (d, *J* = 7.3 Hz, 1H), 6.80 – 6.69 (m, 4H), 5.07 (s, 2H), 3.77 (s, 3H), 2.41 (s, 3H). ^{13}C NMR (126 MHz, $CDCl_3$) δ 158.8, 148.1, 138.8, 137.9, 134.5, 131.2, 131.1, 129.9, 129.7, 129.3, 129.0, 128.8, 128.6, 128.1, 127.3, 126.8, 126.3, 113.9, 113.7, 55.2, 47.8, 21.4. HRMS. Calculated for $C_{30}H_{27}N_2O$ ($M+H^+$): 431.2118, found: 431.2125.

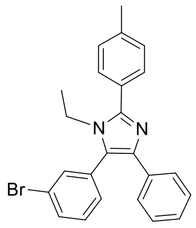

5-(3-Bromophenyl)-1-ethyl-4-phenyl-2-(p-tolyl)-1H-imidazole 1f. Pale yellow solid, 159 mg, 76% yield. ^1H NMR (500 MHz, $CDCl_3$) δ 7.89 – 7.57 (m, 4H), 7.54 (d, *J* = 7.2 Hz, 1H), 7.45 – 7.36 (m, 2H), 7.33 (d, *J* = 8.1 Hz, 2H), 7.25 (dd, *J* = 8.1, 6.8 Hz, 3H), 7.18 (t, *J* = 7.3 Hz, 0H), 7.05 (t, *J* = 7.9 Hz, 1H), 4.00 – 3.93 (m, 2H), 2.45 (s, 3H), 1.05 (t, *J* = 7.2 Hz, 3H). ^{13}C NMR (126 MHz, Chloroform-*d*) δ 148.1, 139.2, 136.7, 133.7, 133.6, 133.3, 132.1, 131.9, 130.7, 130.6, 129.9, 129.8, 129.7, 129.6, 129.4, 129.4, 129.4, 129.1, 129.0, 128.2, 128.1, 128.0, 127.5, 126.9, 126.6, 125.1, 123.0, 122.9, 122.5, 39.8, 21.4, 16.3. Calculated for $C_{24}H_{22}N_2Br$ ($M+H^+$): 417.0961, found: 417.0963.

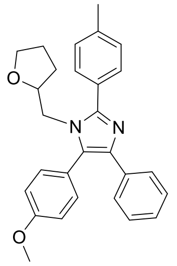

5-(4-Methoxyphenyl)-4-phenyl-1-((tetrahydrofuran-2-yl)methyl)-2-(p-tolyl)-1H-imidazole 1g. White solid, 168 mg, 79% yield. ^1H NMR (500 MHz, $CDCl_3$). δ 7.64 (d, *J* = 8.1 Hz, 2H), 7.57 (d, *J* = 7.0 Hz, 2H), 7.37 (d, *J* = 8.7 Hz, 2H), 7.32 – 7.26 (m, 2H), 7.21 (dd, *J* = 8.4, 6.9 Hz, 2H), 7.13 (t, *J* = 7.2 Hz, 1H), 7.02 (d, *J* = 8.7 Hz, 2H), 4.02 (dd, *J* = 14.3, 7.5 Hz, 2H), 3.89 (s, 3H), 3.74 (dd, *J* = 7.4, 5.7 Hz, 1H), 3.60 – 3.35 (m, 2H), 2.43 (s, 3H), 1.80 – 1.47 (m, 2H), 1.26 – 1.14 (m, 2H). ^{13}C NMR (126 MHz, $CDCl_3$) δ 159.7, 148.1, 138.5, 137.6, 134.9, 132.5, 129.4, 129.3, 129.2, 128.9, 127.9, 126.9, 126.0, 123.6, 114.5, 77.1, 67.7, 55.3, 48.5, 29.0, 25.1, 21.4. HRMS. Calculated for $C_{28}H_{29}N_2O_2$ ($M+H^+$): 425.2224, found: 425.2229.

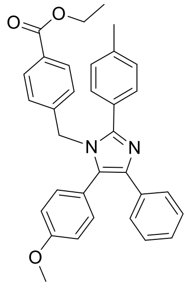

Ethyl 4-((5-(4-methoxyphenyl)-4-phenyl-2-(p-tolyl)-1H-imidazol-1-yl)methyl) benzoate **1h.** Pale yellow solid, 201 mg, 80% yield. ^1H NMR (500 MHz, $CDCl_3$). δ 7.93 (d, *J* = 8.2 Hz, 2H), 7.63 (d, *J* = 7.3 Hz, 2H), 7.53 (d, *J* = 8.1 Hz, 2H), 7.26 – 7.19 (m, 5H), 7.14 (d, *J* = 8.7 Hz, 2H), 6.93 (d, *J* = 8.2 Hz, 2H), 6.86 (d, *J* = 8.7 Hz, 2H), 5.14 (s, 2H), 4.38 (q, *J* = 7.2 Hz, 2H), 3.82 (s, 3H), 2.38 (s, 3H), 1.40 (t, *J* = 7.1 Hz, 3H). ^{13}C NMR (126 MHz, $CDCl_3$) δ 166.2, 159.9, 148.0, 142.8, 138.9, 138.0, 134.5, 132.3, 129.9, 129.7, 129.6, 129.3, 128.8, 128.1, 127.9, 126.7, 126.3, 125.9, 122.9, 114.4, 61.0, 55.2, 48.0, 21.3, 14.3. HRMS. Calculated for $C_{33}H_{31}N_2O_3$ ($M+H^+$): 503.2329, found: 503.2342.

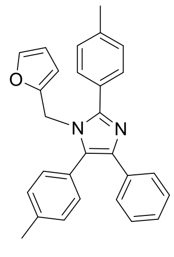

1-(furan-2-ylmethyl)-4-phenyl-2,5-di-p-tolyl-1H-imidazole 1i. Yellow solid, 140 mg, 69% yield. ^{1}H NMR (500 MHz, $CDCl_3$). δ 7.64 (d, *J* = 8.1 Hz, 2H), 7.59 (d, *J* = 7.2 Hz, 2H), 7.33 – 7.27 (m, 3H), 7.26 – 7.19 (m, 6H), 7.15 (t, *J* = 7.3 Hz, 1H), 6.22 (dd, *J* = 3.2, 1.8 Hz, 1H), 5.71 (d, *J* = 2.9 Hz, 1H), 5.01 (s, 2H), 2.44 (s, 3H), 2.43 (s, 3H).^{13}C NMR (126 MHz, $CDCl_3$) δ 150.1, 147.9, 142.1, 138.6, 137.4, 131.0, 129.7, 129.6, 129.3, 129.2, 128.0, 127.9, 126.8, 126.3, 110.4, 108.0, 42.1, 21.4, 21.4. HRMS. Calculated for $C_{28}H_{25}N_2O$ (M+H$^+$): 405.1963, found: 405.1973.

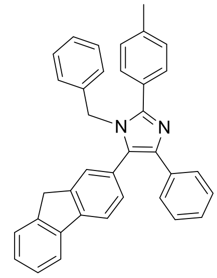

1-benzyl-5-(9H-fluoren-2-yl)-4-phenyl-2-(p-tolyl)-1H-imidazole 1j. Pale yellow solid, 185 mg, 76% yield. ^{1}H NMR (500 MHz, $CDCl_3$). δ 7.83 (d, *J* = 7.5 Hz, 1H), 7.77 (d, *J* = 7.8 Hz, 1H), 7.69 (d, *J* = 7.3 Hz, 2H), 7.64 (d, *J* = 8.1 Hz, 2H), 7.58 (d, *J* = 7.5 Hz, 1H), 7.43 (t, *J* = 7.3 Hz, 1H), 7.39 – 7.33 (m, 2H), 7.30 – 7.20 (m, 8H), 7.17 (t, *J* = 7.2 Hz, 1H), 6.87 (dd, *J* = 6.5, 2.7 Hz, 2H), 5.17 (s, 2H), 3.83 (s, 2H), 2.42 (s, 3H). ^{13}C NMR (126 MHz, $CDCl_3$) δ 148.2, 143.6, 143.5, 142.0, 141.1, 138.8, 137.9, 137.8, 134.7, 130.3, 129.8, 129.4, 129.3, 129.0, 128.6, 128.1, 127.8, 127.3, 127.2, 127.0, 126.8, 126.3, 126.1, 125.2, 120.2, 48.4, 36.9, 21.4. HRMS. Calculated for $C_{36}H_{29}N_2$ (M+H$^+$): 489.2325, found: 489.2329.

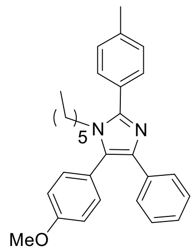

1-Hexyl-5-(4-methoxyphenyl)-4-phenyl-2-(p-tolyl)-1H-imidazole 1k. White solid, 161 mg, 76% yield. ^{1}H NMR (500 MHz, $CDCl_3$) δ 7.60 (m, 4H), 7.35 (d, *J* = 8.7 Hz, 2H), 7.31 (d, *J* = 7.9 Hz, 2H), 7.22 (t, *J* = 7.6 Hz, 2H), 7.14 (t, *J* = 7.3 Hz, 1H), 7.03 (d, *J* = 8.7 Hz, 2H), 3.90 (s, 3H), 3.89 – 3.85 (m, 2H), 2.44 (s, 3H), 1.43 – 1.34 (m, 2H), 1.10 (m, 2H), 0.99 (m, 4H), 0.78 (t, *J* = 7.3 Hz, 3H). ^{13}C NMR (126 MHz, $CDCl_3$) δ 159.8, 147.5, 138.7, 137.4, 134.7, 132.3, 129.9, 129.3, 129.3, 129.1, 128.6, 128.0, 126.8, 126.1, 123.6, 114.5, 55.3, 44.6, 30.8, 30.3, 25.9, 22.2, 21.4, 13.9. HRMS. Calculated for $C_{29}H_{33}N_2O$ (M+H$^+$): 425.2587, found: 425.2591.

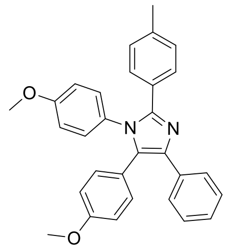

1,5-bis(4-methoxyphenyl)-4-phenyl-2-(p-tolyl)-1H-imidazole 1l. Yellow solid, 165 mg, 74% yield. ^{1}H NMR (500 MHz, $CDCl_3$) δ 7.65 (d, *J* = 7.4 Hz, 2H), 7.37 (d, *J* = 8.1 Hz, 2H), 7.28 (dd, *J* = 10.6, 4.4 Hz, 2H), 7.20 (t, *J* = 7.3 Hz, 1H), 7.08 (d, *J* = 8.6 Hz, 4H), 6.99 (d, *J* = 8.8 Hz, 2H), 6.79 (d, *J* = 7.7

Hz, 4H), 3.80 (s, 3H), 3.80 (s, 3H), 2.33 (s, 3H).^{13}C NMR (126 MHz, $CDCl_3$) δ 159.2, 159.0, 147.0, 137.9, 137.7, 134.8, 132.4, 130.8, 130.2, 129.5, 128.8, 128.7, 128.1, 127.9, 127.3, 126.3, 123.1, 114.2, 113.8, 55.4, 55.1, 21.3. HRMS. Calculated for $C_{30}H_{27}N_2O_2$ (M+H$^+$): 447.2067, found: 447.2067.

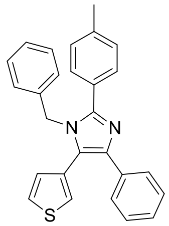

1-benzyl-4-phenyl-5-(thiophen-3-yl)-2-(p-tolyl)-1H-imidazole 1m. White solid, 150 mg, 74% yield. ^{1}H NMR (500 MHz, $CDCl_3$) δ 7.64 (d, *J* = 8.1 Hz, 2H), 7.56 (d, *J* = 8.1 Hz, 2H), 7.33 – 7.25 (m, 6H), 7.21 (dd, *J* = 10.4, 7.6 Hz, 3H), 7.11 (dd, *J* = 2.9, 1.2 Hz, 1H), 6.94 (d, *J* = 6.7 Hz, 2H), 6.88 (dd, *J* = 5.0, 1.2 Hz, 1H), 5.14 (s, 2H), 2.39 (s, 3H).^{13}C NMR (126 MHz, $CDCl_3$) δ 148.4, 138.9, 138.0, 134.6, 130.8, 129.5, 129.3, 128.8, 128.8, 128.1, 128.0, 127.4, 126.7, 126.4, 126.3, 126.0, 125.8, 124.6, 48.4, 21.4. HRMS. Calculated for $C_{27}H_{23}N_2S$ (M+H$^+$): 407.1576, found: 407.1595.

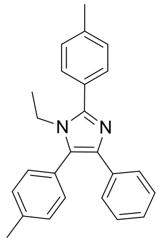

1-Ethyl-4-phenyl-2,5-di-p-tolyl-1H-imidazole **1n.** white solid, 134 mg, 76% yield. ^{1}H NMR (500 MHz, $CDCl_3$) δ 7.62 (d, *J* = 8.1 Hz, 2H), 7.58 (d, *J* = 7.2 Hz, 2H), 7.39 – 7.29 (m, 8H), 7.22 (t, *J* = 7.6 Hz, 2H), 7.14 (t, *J* = 7.3 Hz, 1H), 3.95 (q, *J* = 7.2 Hz, 2H), 2.47 (s, 3H), 2.45 (s, 3H), 1.04 (t, *J* = 7.2 Hz, 3H).^{13}C NMR (126 MHz, $CDCl_3$) δ 147.3, 138.7, 138.5, 137.5, 134.8, 130.9, 129.8, 129.29, 129.28, 129.0, 128.63, 128.59, 128.0, 126.7, 126.0, 39.5, 21.4, 21.4, 16.3. HRMS. Calculated for $C_{25}H_{25}N_2$ (M+H$^+$): 353.2012, found: 353.2018.

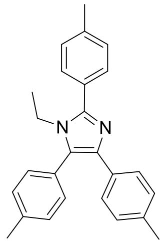

1-Ethyl-2,4,5-tri-p-tolyl-1H-imidazole 1o. Pale yellow solid, 143 mg, 78% yield. ^{1}H NMR (500 MHz, $CDCl_3$) δ 7.62 (d, *J* = 8.1 Hz, 2H), 7.47 (d, *J* = 8.2 Hz, 2H), 7.32 (dt, *J* = 8.0, 6.4 Hz, 6H), 7.04 (d, *J* = 8.0 Hz, 2H), 3.94 (q, *J* = 7.2 Hz, 2H), 2.47 (s, 3H), 2.45 (s, 3H), 2.30 (s, 3H), 1.04 (t, *J* = 7.2 Hz, 3H). ^{13}C NMR (126 MHz, $CDCl_3$) δ 147.2, 138.6, 138.4, 137.6, 135.6, 132.0, 131.0, 129.7, 129.3, 129.1, 128.8, 128.72, 128.71, 128.68, 126.7, 39.5, 21.44, 21.39, 21.1, 16.3. HRMS. Calculated for $C_{26}H_{27}N_2$ (M+H$^+$): 367.2169, found: 367.2162.

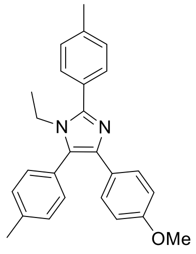

1-Ethyl-4-(4-methoxyphenyl)-2,5-di-p-tolyl-1H-imidazole 1p. Pale yellow solid, 153 mg, 80% yield. ^{1}H NMR (500 MHz, $CDCl_3$) δ 7.62 (d, *J* = 8.1 Hz, 2H), 7.51 (d, *J* = 8.9 Hz, 2H), 7.32 (m, 6H), 6.78 (d, *J* = 8.9 Hz, 2H), 3.94 (q, *J* = 7.1 Hz, 2H), 3.77 (s, 3H), 2.46 (s, 3H), 2.44 (s, 3H), 1.03 (t, *J* = 7.2 Hz, 3H). ^{13}C NMR (126 MHz, $CDCl_3$) δ 158.1, 147.1, 138.6, 138.3, 137.4, 131.0, 129.8, 129.3, 129.0, 128.8, 128.7, 128.3, 127.9, 127.7, 113.5, 55.1, 39.5, 21.43, 21.38, 16.3. HRMS. Calculated for $C_{26}H_{27}N_2O$ (M+H$^+$): 383.2118, found: 383.2120.

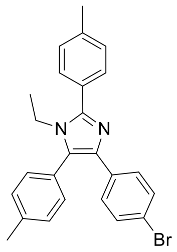

4-(4-bromophenyl)-1-ethyl-2,5-di-p-tolyl-1H-imidazole 1q. Pale yellow solid, 166 mg, 77% yield. ^{1}H NMR (500 MHz, $CDCl_3$) δ 7.60 (d, *J* = 8.1 Hz, 2H), 7.44 (d, *J* = 8.7 Hz, 2H), 7.35 – 7.29 (m, 8H), 3.93 (q, *J* = 7.2 Hz, 2H), 2.47 (s, 3H), 2.45 (s, 3H), 1.03 (t, *J* = 7.2 Hz, 3H). ^{13}C NMR (126 MHz, $CDCl_3$) δ 147.5, 138.8, 138.8, 136.5, 133.9, 131.1, 130.8, 129.9, 129.6, 129.3, 129.0, 128.4, 128.3, 128.2, 119.9, 39.6, 21.44, 21.39, 16.3. HRMS. Calculated for $C_{25}H_{24}N_2Br$ (M+H$^+$): 431.1117, found: 431.1132.

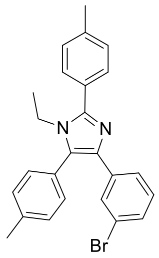

4-(3-Bromophenyl)-1-ethyl-2,5-di-p-tolyl-1H-imidazole 1r. Pale yellow solid, 162 mg, 75% yield. ^{1}H NMR (500 MHz, $CDCl_3$) δ 7.88 (t, *J* = 1.8 Hz, 1H), 7.60 (d, *J* = 8.1 Hz, 2H), 7.35 – 7.30 (m, 7H), 7.25 (ddd, *J* = 7.9, 2.0, 1.0 Hz, 1H), 7.02 (t, *J* = 7.9 Hz, 1H), 3.93 (q, *J* = 7.2 Hz, 2H), 2.48 (s, 3H), 2.45 (s, 3H), 1.03 (t, *J* = 7.2 Hz, 3H). ^{13}C NMR (126 MHz, $CDCl_3$) δ 147.5, 138.9, 138.8, 137.0, 136.1, 130.8, 130.0, 129.9, 129.6, 129.4, 129.3, 129.0, 128.9, 128.3, 128.1, 125.0, 122.4, 39.6, 21.5, 21.4, 16.3. HRMS. Calculated for $C_{25}H_{24}N_2Br$ (M+H$^+$): 431.1117, found: 431.1107.

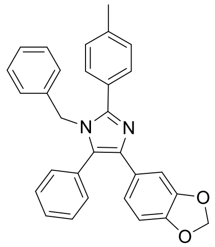

4-(Benzo[d][1,3]dioxol-5-yl)-1-benzyl-5-phenyl-2-(p-tolyl)-1H-imidazole 1s. Pale yellow solid, 171 mg, 77% yield. ^{1}H NMR (500 MHz, $CDCl_3$) δ 7.55 (d, *J* = 8.1 Hz, 2H), 7.38 – 7.30 (m, 3H), 7.21 (td, *J* = 5.3, 2.2 Hz, 8H), 7.11 (dd, *J* = 8.1, 1.7 Hz, 1H), 7.08 (d, *J* = 1.5 Hz, 1H), 6.82 (dd, *J* = 7.2, 2.2 Hz, 2H), 6.69 (d, *J* = 8.1 Hz, 1H), 5.90 (s, 2H), 5.10 (s, 2H), 2.39 (s, 3H). ^{13}C

NMR (126 MHz, $CDCl_3$) δ δ 147.9, 147.3, 146.1, 138.8, 137.7, 131.1, 129.3, 129.1, 128.9, 128.8, 128.6, 128.5, 127.3, 126.0, 120.5, 108.1, 107.6, 100.7, 48.3, 21.3 HRMS. Calculated for $C_{30}H_{25}N_2O_2$ ($M+H^+$): 445.1911, found: 445.1921.

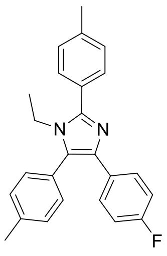

1-Ethyl-4-(4-fluorophenyl)-2,5-di-p-tolyl-1H-imidazole 1t. Pale yellow solid, 141 mg, 76% yield. ^{1}H NMR (500 MHz, $CDCl_3$) δ 7.61 (d, *J* = 8.1 Hz, 2H), 7.53 (dd, *J* = 9.0, 5.5 Hz, 2H), 7.36 – 7.30 (m, 6H), 6.90 (t, *J* = 8.9 Hz, 2H), 3.94 (q, *J* = 7.2 Hz, 2H), 2.47 (s, 3H), 2.45 (s, 3H), 1.03 (t, *J* = 7.2 Hz, 3H). ^{13}C NMR (126 MHz, $CDCl_3$) δ 161.51 (d, *J* = 244.6 Hz), 147.3, 138.8, 138.6, 136.7, 131.0 (d, *J* = 3.0 Hz), 130.9, 129.9, 129.3, 129.2, 129.0, 128.5, 128.4, 128.3 (d, *J* = 7.8 Hz), 114.8 (d, *J* = 21.2 Hz), 39.6, 21.43, 21.38, 16.3. HRMS. Calculated for $C_{25}H_{24}N_2F$ ($M+H^+$): 371.1918, found: 371.1923.

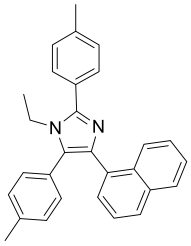

1-Ethyl-4-(naphthalen-1-yl)-2,5-di-p-tolyl-1H-imidazole 1u. Pale yellow solid, 163 mg, 81% yield. ^{1}H NMR (500 MHz, $CDCl_3$) δ 8.17 (s, 1H), 7.74 (dd, *J* = 8.5, 6.4 Hz, 2H), 7.69 – 7.64 (m, 3H), 7.60 (m, 1H), 7.43 – 7.31 (m, 8H), 3.98 (q, *J* = 7.1 Hz, 2H), 2.49 (s, 3H), 2.47 (s, 3H), 1.06 (t, *J* = 7.2 Hz, 3H). ^{13}C NMR (126 MHz, $CDCl_3$) δ 147.5, 138.8, 138.6, 137.5, 133.6, 132.3, 132.2, 133.0, 129.8, 129.8, 129.3, 129.1, 129.0, 128.6, 128.2, 127.5, 127.3, 125.6, 125.4, 125.1, 125.1, 39.6, 21.5, 21.4, 16.3. HRMS. Calculated for $C_{29}H_{27}N_2$ ($M+H^+$): 403.2169, found: 403.2177.

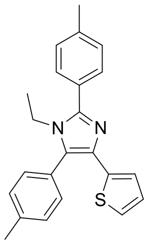

1-Ethyl-4-(thiophen-2-yl)-2,5-di-p-tolyl-1H-imidazole 1v. White solid, 147 mg, 82% yield. ^{1}H NMR (500 MHz, $CDCl_3$) δ 7.60 (d, *J* = 8.0 Hz, 2H), 7.37 (q, *J* = 8.0 Hz, 4H), 7.31 (d, *J* = 7.9 Hz, 2H), 7.07 (d, *J* = 5.0 Hz, 1H), 6.95 (d, *J* = 2.7 Hz, 1H), 6.88 (dd, *J* = 4.9, 3.7 Hz, 1H), 3.91 (q, *J* = 7.1 Hz, 2H), 2.49 (s, 3H), 2.44 (s, 3H), 1.05 (t, *J* = 7.2 Hz, 3H). ^{113}C NMR (126 MHz, $CDCl_3$) δ 147.2, 139.0, 138.8, 138.5, 133.5, 131.1, 129.8, 129.3, 129.1, 128.3, 128.3, 127.7, 127.0, 122.9, 122.3, 39.7, 21.5, 21.4, 16.3. HRMS. Calculated for $C_{23}H_{23}N_2S$ ($M+H^+$): 359.1576, found: 359.1581.

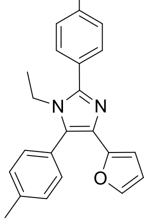

1-Ethyl-4-(furan-2-yl)-2,5-di-p-tolyl-1H-imidazole 1w. Pale yellow solid, 137 mg, 80% yield. ^{1}H NMR (500 MHz, $CDCl_3$) δ 7.57 (d, *J* = 8.1 Hz, 2H), 7.38 – 7.19

(m, 7H), 6.27 (dd, *J* = 3.3, 1.8 Hz, 1H), 6.09 (dd, *J* = 3.3, 0.7 Hz, 1H), 3.92 (q, *J* = 7.2 Hz, 2H), 2.44 (s, 3H), 2.41 (s, 3H), 0.99 (t, *J* = 7.2 Hz, 3H). ^{13}C NMR (126 MHz, $CDCl_3$). HRMS. Calculated for $C_{23}H_{23}N_2O$ (M+H$^+$): 343.1805, found: 343.1814.

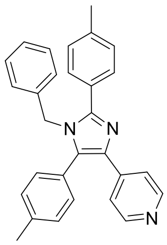

4-(1-benzyl-2,5-di-p-tolyl-1H-imidazol-4-yl)pyridine 1x. Pale yellow solid, 122 mg, 59% yield. ^{1}H NMR (500 MHz, $CDCl_3$δ 8.41 (d, *J* = 6.3 Hz, 2H), 7.53 (d, *J* = 8.1 Hz, 2H), 7.47 (d, *J* = 6.3 Hz, 2H), 7.24 – 7.21 (m, 5H), 7.18 (d, *J* = 7.9 Hz, 2H), 7.10 (d, *J* = 8.1 Hz, 2H), 6.84 – 6.78 (m, 2H), 5.09 (s, 2H), 2.40 (s, 3H), 2.38 (s, 3H). ^{13}C NMR (126 MHz, $CDCl_3$) δ 149.6, 148.7, 142.2, 139.2, 139.2, 137.3, 135.1, 132.6, 130.6, 129.8, 129.4, 128.9, 128.6, 127.6, 127.4, 127.1, 126.0, 120.6, 48.2, 21.4, 21.4. HRMS. Calculated for $C_{29}H_{26}N_3$ (M+H$^+$): 416.2121, found: 416.2131.

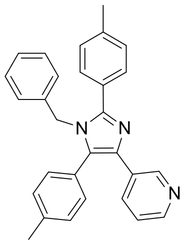

3-(1-benzyl-2,5-di-p-tolyl-1H-imidazol-4-yl)pyridine 1y. Pale yellow solid, 96 mg, 46% yield. ^{1}H NMR (500 MHz, $CDCl_3$) δ 8.71 (d, *J* = 1.5 Hz, 1H), 8.38 (dd, *J* = 4.7, 1.7 Hz, 1H), 7.98 (d, *J* = 8.1 Hz, 1H), 7.54 (d, *J* = 8.1 Hz, 2H), 7.22 (d, *J* = 7.3 Hz, 5H), 7.19 – 7.07 (m, 5H), 6.87 – 6.83 (m, 2H), 5.12 (s, 2H), 2.39 (s, 3H), 2.38 (s, 3H).^{13}C NMR (126 MHz, $CDCl_3$) δ 148.6, 148.0, 147.2, 139.0, 138.9, 137.5, 135.1, 133.7, 131.0, 130.7, 130.7, 129.8, 129.3, 128.9, 128.6, 127.8, 127.4, 127.2, 126.0, 123.1, 48.2, 21.4. HRMS. Calculated for $C_{29}H_{26}N_3$ (M+H$^+$): 416.2121, found: 416.2123.

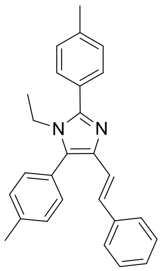

(E)-1-Ethyl-4-styryl-2,5-di-p-tolyl-1H-imidazole 1z. Pale yellow solid, 141 mg, 74% yield. ^{1}H NMR (500 MHz, $CDCl_3$) δ 7.60 (d, *J* = 8.1 Hz, 2H), 7.46 (m, 3H), 7.36 (m, 4H), 7.34 – 7.25 (m, 4H), 7.18 (t, *J* = 7.3 Hz, 1H), 6.92 (d, *J* = 16.0 Hz, 1H), 4.01 (q, *J* = 7.1 Hz, 2H), 2.49 (s, 3H), 2.45 (s, 3H), 0.99 (t, *J* = 7.2 Hz, 3H). ^{13}C NMR (126 MHz, $CDCl_3$) δ 148.4, 138.9, 138.3, 138.3, 136.9, 131.7, 130.4, 129.6, 129.3, 129.1, 128.4, 127.2, 126.84, 126.82, 126.7, 126.2, 119.6, 39.8, 21.41, 21.40, 16.1. HRMS. Calculated for $C_{27}H_{27}N_2$ (M+H$^+$): 379.2169, found: 379.2180.

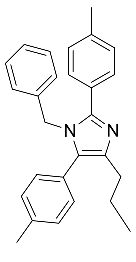

1-benzyl-4-propyl-2,5-di-p-tolyl-1H-imidazole 1aa. Colorless oil, 155 mg, 81% yield. ^{1}H NMR (500 MHz, $CDCl_3$) δ 7.49 (d, *J* = 8.1 Hz, 2H), 7.24 – 7.16 (m, 5H), 7.14 (d, *J* = 7.9 Hz, 2H), 7.10 (d, *J* = 8.2 Hz, 2H), 6.79 (d, *J* = 6.3 Hz, 2H), 5.12 (s,

2H), 2.57 (dd, *J* = 8.4, 7.0 Hz, 2H), 2.36 (s, 6H), 1.74 (h, *J* = 7.4 Hz, 2H), 0.93 (t, *J* = 7.4 Hz, 3H). ^{13}C NMR (126 MHz, $CDCl_3$) δ 147.4, 140.0, 138.3, 138.2, 137.6, 130.5, 129.9, 129.13, 129.08, 128.8, 128.5, 128.4, 127.7, 127.1, 125.9, 48.3, 29.6, 23.6, 21.3, 21.3, 14.1 Calculated for $C_{27}H_{29}N_2$ (M+H$^+$): 381.2325, found: 381.2333.

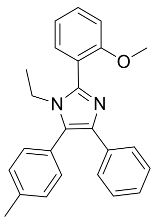

1-Ethyl-2-(2-methoxyphenyl)-4-phenyl-5-(p-tolyl)-1H-imidazole 2a. White solid, 112 mg, 61% yield. ^{1}H NMR (500 MHz, $CDCl_3$) δ 7.58 (d, *J* = 7.5 Hz, 2H), 7.49 – 7.41 (m, 1H), 7.36 (d, *J* = 7.9 Hz, 2H), 7.32 – 7.25 (m, 3H), 7.20 (t, *J* = 7.6 Hz, 2H), 7.11 (dt, *J* = 11.1, 7.4 Hz, 2H), 7.02 (d, *J* = 8.3 Hz, 1H), 3.86 (s, 3H), 3.77 (q, *J* = 7.2 Hz, 2H), 2.47 (s, 3H), 0.93 (t, *J* = 7.2 Hz, 3H). ^{13}C NMR (126 MHz, $CDCl_3$) δ 157.6, 144.6, 138.3, 137.4, 134.8, 132.7, 130.9, 130.8, 129.7, 128.8, 128.7, 127.9, 126.7, 125.9, 120.9, 120.7, 111.0, 55.6, 39.5, 21.4, 15.9. HRMS. Calculated for $C_{25}H_{25}N_2O$ (M+H$^+$): 369.1961, found: 369.1966.

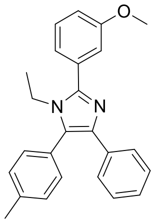

1-Ethyl-2-(3-methoxyphenyl)-4-phenyl-5-(p-tolyl)-1H-imidazole 2b. Pale yellow solid, 151 mg, 82% yield. ^{1}H NMR (500 MHz, $CDCl_3$) δ 7.58 (d, *J* = 7.2 Hz, 2H), 7.41 (t, *J* = 7.9 Hz, 1H), 7.35 – 7.27 (m, 6H), 7.22 (t, *J* = 7.5 Hz, 2H), 7.14 (t, *J* = 7.3 Hz, 1H), 7.02 (dd, *J* = 8.2, 2.5 Hz, 1H), 3.96 (q, *J* = 7.2 Hz, 2H), 3.90 (s, 3H), 2.47 (s, 3H), 1.05 (t, *J* = 7.2 Hz, 3H). ^{13}C NMR (126 MHz, $CDCl_3$) δ 159.7, 147.0, 138.6, 137.6, 134.3, 132.8, 130.0, 129.1, 129.6, 129.5, 128.5, 128.0, 126.7, 126.1, 121.4, 114.9, 114.5, 55.4, 39.6, 21.5, 16.3. HRMS. Calculated for $C_{25}H_{25}N_2O$ (M+H$^+$): 369.1961, found: 369.1961.

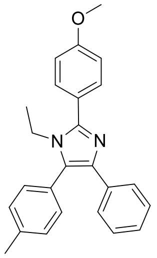

1-Ethyl-2-(4-methoxyphenyl)-4-phenyl-5-(p-tolyl)-1H-imidazole 2c. Pale brown solid, 158 mg, 86% yield. ^{1}H NMR (500 MHz, $CDCl_3$) δ 7.66 (d, *J* = 8.8 Hz, 2H), 7.57 (d, *J* = 7.1 Hz, 2H), 7.36 – 7.29 (m, 4H), 7.22 (t, *J* = 7.5 Hz, 2H), 7.17 – 7.11 (m, 1H), 7.03 (d, *J* = 8.8 Hz, 2H), 3.93 (q, *J* = 7.2 Hz, 2H), 3.88 (s, 3H), 2.47 (s, 3H), 1.04 (t, *J* = 7.2 Hz, 3H). ^{13}C NMR (126 MHz, $CDCl_3$) δ 160.0, 147.1, 138.5, 137.4, 134.8, 130.9, 130.5, 129.8, 129.2, 128.6, 128.0, 126.7, 126.0, 124.0, 114.0, 55.4, 39.5, 21.4, 16.3. HRMS. Calculated for $C_{25}H_{25}N_2O$ (M+H$^+$): 369.1961, found: 369.1962.

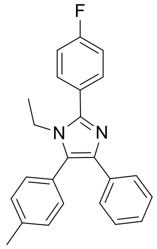

1-Ethyl-2-(4-fluorophenyl)-4-phenyl-5-(p-tolyl)-1H-imidazole 2d. Pale yellow solid, 132 mg, 74% yield. ^{1}H NMR (500 MHz, $CDCl_3$) δ 7.72 (dd, *J* = 8.8, 5.4 Hz, 2H), 7.57 (d, *J* = 7.2 Hz, 2H), 7.35 – 7.30 (m, 4H), 7.21 (dt, *J* = 10.7, 8.1 Hz, 4H), 7.15 (t, *J* = 7.3 Hz, 1H), 3.92 (q, *J* = 7.2 Hz, 2H), 2.47 (s, 3H), 1.04 (t, *J* = 7.2 Hz, 3H). ^{13}C NMR (126 MHz, $CDCl_3$) δ 163.1 (d, *J* = 248.7 Hz), 146.2, 138.6, 137.7, 134.7, 131.0 (d, *J* = 8.4 Hz), 130.9, 129.9, 129.5, 128.4, 128.0, 127.7 (d, *J* = 3.3 Hz), 126.7, 126.2, 115.7 (d, *J* = 21.7 Hz), 39.6, 21.5, 16.3. HRMS. Calculated for $C_{24}H_{22}N_2F$ ($M+H^+$): 357.1762, found: 357.1767.

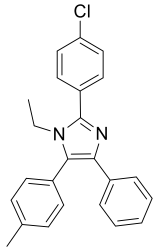

2-(4-Chlorophenyl)-1-ethyl-4-phenyl-5-(p-tolyl)-1H-imidazole 2e. White solid, 145 mg, 78% yield. ^{1}H NMR (500 MHz, $CDCl_3$) δ 7.69 (d, *J* = 8.5 Hz, 2H), 7.55 (d, *J* = 7.3 Hz, 2H), 7.49 (d, *J* = 8.5 Hz, 2H), 7.35 – 7.31 (m, 4H), 7.22 (t, *J* = 7.5 Hz, 2H), 7.15 (t, *J* = 7.3 Hz, 1H), 3.94 (q, *J* = 7.2 Hz, 2H), 2.48 (s, 3H), 1.05 (t, *J* = 7.2 Hz, 3H). ^{13}C NMR (126 MHz, $CDCl_3$) δ 145.9, 138.7, 137.8, 134.9, 134.5, 130.9, 130.4, 129.9, 129.9, 129.8, 128.9, 128.2, 128.1, 126.7, 126.3, 39.6, 21.5, 16.3. HRMS. Calculated for $C_{24}H_{22}N_2Cl$ ($M+H^+$): 373.1466, found: 373.1469.

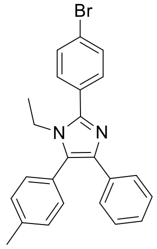

2-(4-Bromophenyl)-1-ethyl-4-phenyl-5-(p-tolyl)-1H-imidazole 2f. Pale yellow solid, 164 mg, 79% yield. ^{1}H NMR (500 MHz, $CDCl_3$) δ 7.63 (q, *J* = 8.7 Hz, 4H), 7.54 (d, *J* = 7.2 Hz, 2H), 7.32 (s, 4H), 7.22 (t, *J* = 7.5 Hz, 2H), 7.15 (t, *J* = 7.3 Hz, 1H), 3.94 (q, *J* = 7.2 Hz, 2H), 2.47 (s, 3H), 1.05 (t, *J* = 7.2 Hz, 3H). ^{13}C NMR (126 MHz, $CDCl_3$) δ 145.9, 138.7, 137.9, 134.5, 131.8, 130.9, 130.6, 130.4, 129.8, 128.2, 128.1, 126.7, 126.2, 123.1, 39.6, 21.4, 16.3. HRMS. Calculated for $C_{24}H_{22}N_2Br$ ($M+H^+$): 417.0961, found: 417.0972.

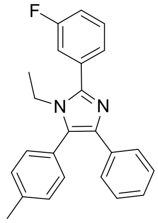

1-Ethyl-2-(3-fluorophenyl)-4-phenyl-5-(p-tolyl)-1H-imidazole 2g. White solid, 128 mg, 72% yield. ^{1}H NMR (500 MHz, $CDCl_3$) δ 7.57 – 7.50 (m, 3H), 7.47 (dd, *J* = 7.9, 2.0 Hz, 2H), 7.32 (s, 4H), 7.23 (d, *J* = 7.3 Hz, 2H), 7.16 (d, *J* = 7.4 Hz, 2H), 3.97 (d, *J* = 7.2 Hz, 2H), 2.48 (s, 3H), 1.06 (t, *J* = 7.2 Hz, 3H). ^{13}C NMR (126 MHz, $CDCl_3$) δ 162.8 (d, *J* = 246.6 Hz), 145.7 (d, *J* = 2.6 Hz), 138.7, 137.9, 134.5,

133.5 (d, J = 8.2 Hz), 130.9, 130.2 (d, J = 8.4 Hz), 129.8, 128.2, 128.0, 126.7, 126.2, 124.70, 124.67, 116.2 (d, J = 22.7 Hz), 115.7 (d, J = 21.1 Hz), 39.6, 21.4, 16.3. HRMS. Calculated for $C_{24}H_{22}N_2F$ (M+H$^+$): 357.1762, found: 357.1769.

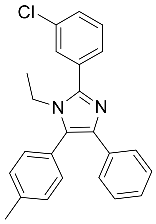

2-(3-Chlorophenyl)-1-ethyl-4-phenyl-5-(p-tolyl)-1H-imidazole 2h. White solid, 142 mg, 76% yield. ^{1}H NMR (500 MHz, $CDCl_3$) δ 7.76 (d, J = 1.0 Hz, 1H), 7.64 – 7.61 (m, 1H), 7.55 (d, J = 7.2 Hz, 2H), 7.46 – 7.44 (m, 2H), 7.32 (s, 4H), 7.23 (t, J = 7.5 Hz, 2H), 7.16 (t, J = 7.3 Hz, 1H), 3.96 (q, J = 7.2 Hz, 2H), 2.48 (s, 3H), 1.06 (t, J = 7.2 Hz, 3H). ^{13}C NMR (126 MHz, $CDCl_3$) δ 145.6, 138.8, 137.8, 134.6, 134.2, 133.0, 130.9, 130.9, 129.9, 129.8, 129.3, 129.0, 128.8, 128.1, 127.1, 126.7, 126.3, 39.7, 21.4, 16.3. Calculated for $C_{24}H_{22}N_2Cl$ (M+H$^+$): 373.1466, found: 373.1468.

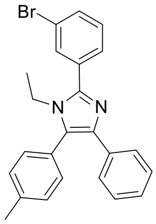

2-(3-Bromophenyl)-1-ethyl-4-phenyl-5-(p-tolyl)-1H-imidazole 2i. White solid, 161 mg, 77% yield. ^{1}H NMR (500 MHz, $CDCl_3$) δ 7.93 (t, J = 1.7 Hz, 1H), 7.66 (d, J = 7.7 Hz, 1H), 7.60 (d, J = 7.1 Hz, 1H), 7.55 (d, J = 7.2 Hz, 2H), 7.38 (t, J = 7.9 Hz, 1H), 7.32 (s, 4H), 7.22 (t, J = 7.5 Hz, 2H), 7.16 (t, J = 7.3 Hz, 1H), 3.96 (q, J = 7.2 Hz, 2H), 2.48 (s, 3H), 1.06 (t, J = 7.2 Hz, 3H). ^{13}C NMR (126 MHz, $CDCl_3$) δ 145.5, 138.8, 137.9, 134.3, 133.3, 132.2, 131.9, 130.9, 130.1, 129.9, 129.87, 128.09, 128.07, 127.5, 126.7, 126.3, 122.7, 39.7, 21.5, 16.3. HRMS. Calculated for $C_{24}H_{22}N_2Br$ (M+H$^+$): 417.0961, found: 417.0969.

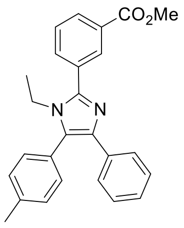

Methyl 3-(1-ethyl-4-phenyl-5-(p-tolyl)-1H-imidazol-2-yl)benzoate 2j. Pale yellow solid, 131 mg, 66% yield. ^{1}H NMR (500 MHz, $CDCl_3$) δ 8.41 (s, 1H), 8.15 (d, J = 7.9 Hz, 1H), 7.99 (d, J = 7.7 Hz, 1H), 7.61 (t, J = 7.8 Hz, 1H), 7.57 (d, J = 7.2 Hz, 2H), 7.35 – 7.31 (m, 4H), 7.23 (t, J = 7.5 Hz, 2H), 7.16 (t, J = 7.3 Hz, 1H), 4.00 – 3.95 (m, 5H), 2.47 (s, 3H), 1.06 (t, J = 7.2 Hz, 3H). ^{13}C NMR (126 MHz, $CDCl_3$) δ 166.6, 145.9, 138.8, 137.6, 134.1, 133.7, 131.4, 130.9, 130.6, 130.0, 129.9, 129.86, 128.9, 128.1, 128.0, 126.8, 126.4, 52.3, 39.8, 21.5, 16.2. HRMS. Calculated for $C_{26}H_{25}N_2O_2$ (M+H$^+$): 397.1911, found: 397.1920.

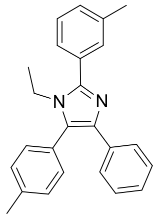

1-Ethyl-4-phenyl-2-(m-tolyl)-5-(p-tolyl)-1H-imidazole 2k. Pale yellow solid, 134 mg, 76% yield. ^{1}H NMR (500 MHz, $CDCl_3$) δ 7.61 – 7.55 (m, 3H), 7.49 (d, *J* = 7.6 Hz, 1H), 7.39 (t, *J* = 7.6 Hz, 1H), 7.33 (q, *J* = 8.1 Hz, 4H), 7.28 (d, *J* = 5.6 Hz, 1H), 7.22 (t, *J* = 7.6 Hz, 2H), 7.15 (t, *J* = 7.3 Hz, 1H), 3.96 (q, *J* = 7.1 Hz, 2H), 2.47 (s, 3H), 2.46 (s, 3H), 1.04 (t, *J* = 7.2 Hz, 3H). ^{13}C NMR (126 MHz, $CDCl_3$) δ 147.4, 138.5, 138.4, 137.6, 134.8, 131.4, 130.9, 130.1, 129.8, 129.6, 129.4, 128.6, 128.4, 128.0, 126.7, 126.1, 126.0, 39.6, 21.5, 21.4, 16.3. HRMS. Calculated for $C_{25}H_{25}N_2$ (M+H$^+$): 353.2012, found: 353.2018.

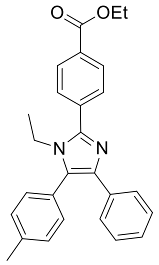

Ethyl 4-(1-ethyl-4-phenyl-5-(p-tolyl)-1H-imidazol-2-yl)benzoate 2l. White solid, 142 mg, 69% yield. ^{1}H NMR (500 MHz, $CDCl_3$) δ 8.19 (d, *J* = 8.5 Hz, 2H), 7.84 (d, *J* = 8.4 Hz, 2H), 7.55 (d, *J* = 7.2 Hz, 2H), 7.33 (s, 4H), 7.23 (t, *J* = 7.5 Hz, 2H), 7.16 (t, *J* = 7.3 Hz, 1H), 4.44 (q, *J* = 7.2 Hz, 2H), 3.99 (q, *J* = 7.2 Hz, 2H), 2.48 (s, 3H), 1.45 (t, *J* = 7.2 Hz, 3H), 1.06 (t, *J* = 7.2 Hz, 3H). ^{13}C NMR (126 MHz, $CDCl_3$) δ 166.3, 146.0, 138.7, 138.2, 135.7, 134.5, 130.8, 130.4, 130.3, 129.8, 129.8, 128.8, 128.1, 128.1, 126.7, 126.3, 61.2, 39.7, 21.4, 16.3, 14.4. HRMS. Calculated for $C_{27}H_{27}N_2O_2$ (M+H$^+$): 411.2067, found: 411.2067.

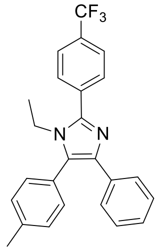

1-Ethyl-4-phenyl-5-(p-tolyl)-2-(4-(trifluoromethyl)phenyl)-1H-imidazole 2m. White solid, 130 mg, 64% yield. ^{1}H NMR (500 MHz, $CDCl_3$) δ 7.90 (d, *J* = 8.1 Hz, 2H), 7.78 (d, *J* = 8.2 Hz, 2H), 7.56 (d, *J* = 7.1 Hz, 2H), 7.33 (m, 4H), 7.23 (t, *J* = 7.4 Hz, 2H), 7.17 (t, *J* = 7.3 Hz, 1H), 3.99 (q, *J* = 7.2 Hz, 2H), 2.48 (s, 3H), 1.07 (t, *J* = 7.2 Hz, 3H). ^{13}C{^{19}F} NMR (126 MHz, $CDCl_3$) δ 145.3, 139.0, 137.8, 134.4, 133.9, 130.8, 130.2, 129.9, 129.4, 128.1, 126.8, 126.5, 125.6, 125.1, 122.9, 39.8, 21.4, 16.3. ^{19}F NMR (470 MHz, $CDCl_3$) δ -62.7. HRMS. Calculated for $C_{25}H_{22}N_2F_3$ (M+H$^+$): 407.1730, found: 407.1727.

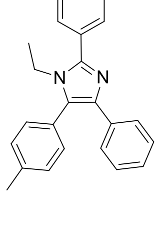

2-(3,5-Dimethylphenyl)-1-ethyl-4-phenyl-5-(p-tolyl)-1H-imidazole 2o. Yellow solid, 152 mg, 83% yield. ^{1}H NMR (500 MHz, $CDCl_3$) δ 7.56 (d, *J* = 7.2 Hz, 2H), 7.36 – 7.29 (m, 6H), 7.21 (t, *J* = 7.5 Hz, 2H), 7.14 (t, *J* = 7.3 Hz, 1H), 7.10 (s, 1H), 3.95 (q, *J* = 7.2 Hz, 2H), 2.47 (s, 3H), 2.41 (s, 6H), 1.04 (t, *J* = 7.2 Hz, 3H). ^{13}C

NMR (126 MHz, $CDCl_3$) δ 147.6, 138.4, 138.1, 137.5, 134.8, 131.3, 130.9, 130.5, 129.8, 129.2, 128.6, 128.0, 127.0, 126.8, 126.0, 39.6, 21.43, 21.37, 16.3. HRMS. Calculated for $C_{26}H_{27}N_2$ ($M+H^+$): 367.2169, found: 367.2174.

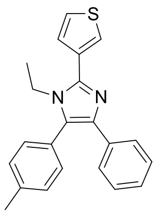

1-Ethyl-4-phenyl-2-(thiophen-3-yl)-5-(p-tolyl)-1H-imidazole 2p. Pale red solid, 136 mg, 79% yield. ^{1}H NMR (500 MHz, $CDCl_3$) δ 7.67 (dd, *J* = 2.9, 1.2 Hz, 1H), 7.57 – 7.53 (m, 3H), 7.45 (dd, *J* = 5.0, 3.0 Hz, 1H), 7.31 (m, 4H), 7.22 (t, *J* = 7.5 Hz, 2H), 7.16 – 7.12 (m, 1H), 3.98 (q, *J* = 7.2 Hz, 2H), 2.47 (s, 3H), 1.14 (t, *J* = 7.2 Hz, 3H). ^{13}C NMR (126 MHz, $CDCl_3$) δ 142.69, 138.59, 137.51, 134.69, 132.07, 130.95, 129.79, 129.33, 128.73, 128.35, 128.20, 128.00, 126.66, 126.57, 126.11, 125.85, 124.30, 39.42, 21.44, 16.29. HRMS. Calculated for $C_{22}H_{21}N_2S$ ($M+H^+$): 345.1420, found: 345.1427.

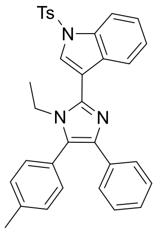

3-(1-Ethyl-4-phenyl-5-(p-tolyl)-1H-imidazol-2-yl)-1-tosyl-1H-indole 2q. White solid, 234 mg, 88% yield. ^{1}H NMR (500 MHz, $CDCl_3$) δ 8.13 (d, *J* = 8.6 Hz, 1H), 7.87 (d, *J* = 1.2 Hz, 1H), 7.79 (d, *J* = 8.4 Hz, 2H), 7.67 (dd, *J* = 8.6, 1.6 Hz, 1H), 7.64 (d, *J* = 3.7 Hz, 1H), 7.57 (d, *J* = 7.2 Hz, 2H), 7.33 (q, *J* = 8.1 Hz, 4H), 7.24 (d, *J* = 8.1 Hz, 2H), 7.21 (t, *J* = 7.5 Hz, 2H), 7.13 (t, *J* = 7.3 Hz, 1H), 6.74 (dd, *J* = 3.6, 0.4 Hz, 1H), 3.95 (q, *J* = 7.1 Hz, 2H), 2.47 (s, 3H), 2.36 (s, 3H), 1.01 (t, *J* = 7.2 Hz, 3H). ^{13}C NMR (126 MHz, $CDCl_3$) δ 147.2, 145.2, 138.6, 137.6, 135.1, 135.0, 134.8, 131.0, 130.9, 130.0, 129.8, 129.4, 128.5, 128.0, 127.3, 126.9, 126.8, 126.7, 126.1, 125.8, 122.3, 113.8, 109.4, 39.6, 21.6, 21.5, 16.3. HRMS. Calculated for $C_{33}H_{30}N_3OS$ ($M+H^+$): 532.2053, found: 532.2065.

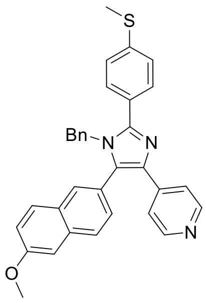

4-(1-benzyl-5-(6-methoxynaphthalen-2-yl)-2-(4-(methylthio) phenyl)-1H-imidazol-4-yl)pyridine Pale yellow solid in 80% yield (413 mg). ^{1}H NMR (500 MHz, $CDCl_3$) δ 8.38 (d, *J* = 6.3 Hz, 2H), 7.74 (d, *J* = 8.4 Hz, 1H), 7.64 – 7.58 (m, 4H), 7.46 (d, *J* = 6.4 Hz, 2H), 7.30 (d, *J* = 8.5 Hz, 2H), 7.26 – 7.22 (m, 4H), 7.19 (d, *J* = 8.7 Hz, 2H), 6.83 (dd, *J* = 6.5, 3.0 Hz, 2H), 5.14 (s, 2H), 3.97 (s, 3H), 2.52 (s, 3H). ^{13}C NMR (126 MHz, $CDCl_3$) δ 158.6, 149.7, 148.3, 141.9, 140.3, 137.3, 135.6, 134.6, 132.8, 130.2, 129.7, 129.2, 128.7, 128.7, 128.4, 127.7, 127.6, 127.0, 126.2, 126.0, 125.0, 120.6, 119.6, 105.6, 55.4, 48.4, 15.4. HRMS. Calculated for $C_{33}H_{28}N_3OS$ ($M+H^+$): 514.1948, found: 514.1964.

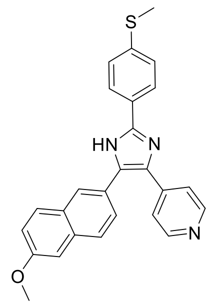

Procedure for the Synthesis of 4-(5-(6-methoxynaphthalen-2-yl)-2-(4-(methylthio)phenyl)-1H-imidazol-4-yl)pyridine 6.

Pale brown solid, 80% yield (67 mg). ^{1}H NMR (500 MHz, DMSO-d_6) δ 13.02 (s, 1H), 8.45 (d, J = 5.8 Hz, 2H), 8.08 (d, J = 8.7 Hz, 3H), 7.88 (t, J = 8.9 Hz, 2H), 7.56 (dd, J = 8.5, 1.6 Hz, 1H), 7.52 (d, J = 6.3 Hz, 2H), 7.38 (d, J = 8.7 Hz, 3H), 7.22 (dd, J = 8.9, 2.4 Hz, 1H), 3.91 (s, 3H), 2.54 (s, 3H). ^{13}C NMR (126 MHz, DMSO-d_6) δ 158.2, 150.1, 139.2, 134.3, 132.1, 131.9, 130.0, 129.1, 128.85, 127.79, 127.78, 127.56, 127.53, 127.26, 127.25, 126.29, 126.25, 121.3, 119.6, 106.5, 55.7, 14.9. HRMS. Calculated for $C_{26}H_{22}N_3OS$ (M+H$^+$): 424.1478, found: 424.1483.

IV. References

1. Layer W. R. Chem. Rev. **1963**, 63, 489.

2. Vishwakarma, L. C.; Stringer, O. D.; Davis F. A. Organic Syntheses, **1988**, 66, 203.

3. Chemla, F.; Hebbe, V.; Normant, J-F. Synthesis, **2000**, *1*, 75.

4. Boultwood, T.; Bull, J. A. *Org. Lett.* **2014**, *16*, 2740.

5. Haddach, A.A.; Kelleman, A.; Deaton-Rewolinski, M.V. *Tetrahedron Lett*. **2002**, *43*, 399.

6. Siamaki, A. R.; Arndtsen, B. A. *J. Am. Chem. Soc.* **2006**, *128*, 6050.

V. NMR Spectra

^{1}H and ^{13}C NMR spectra of 1a

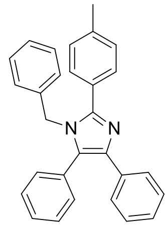

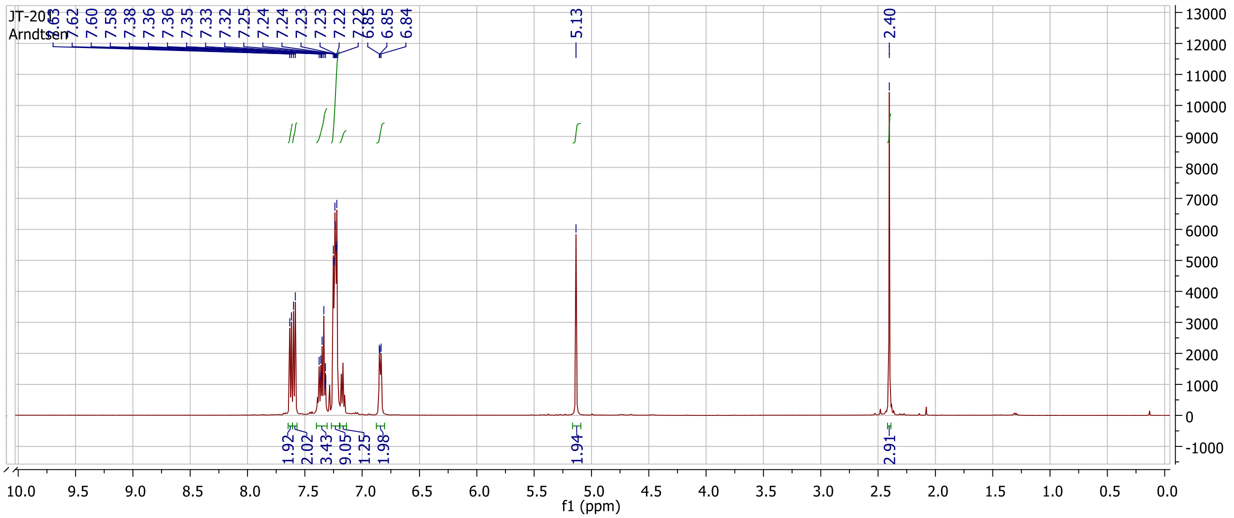

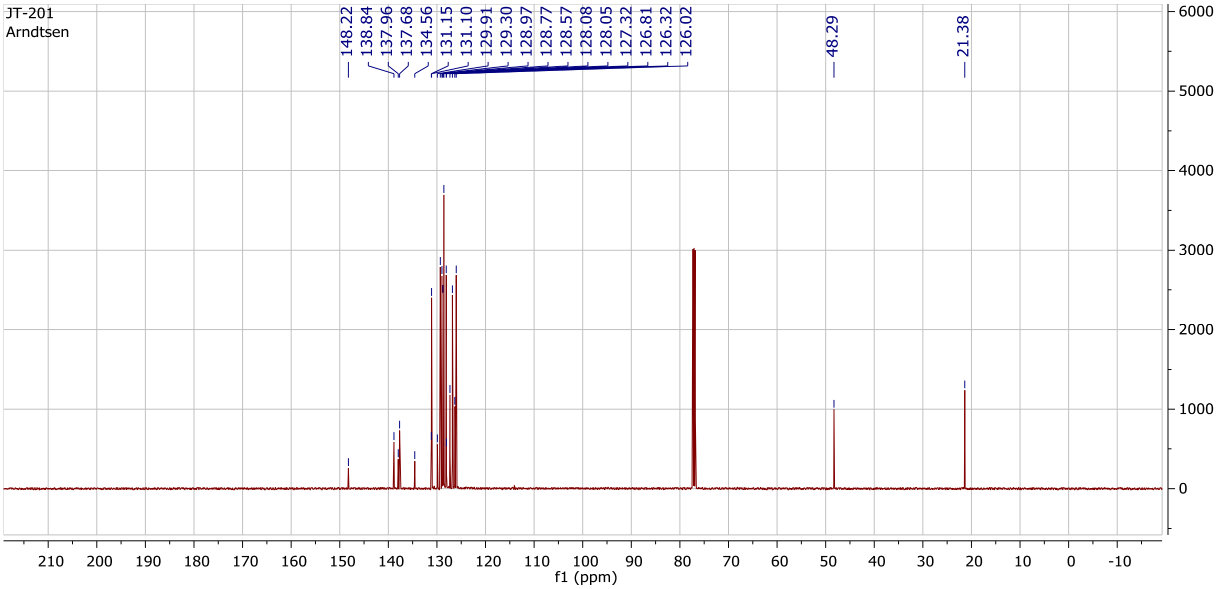

^{1}H and ^{13}C NMR spectra of 1b

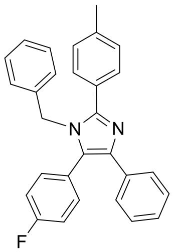

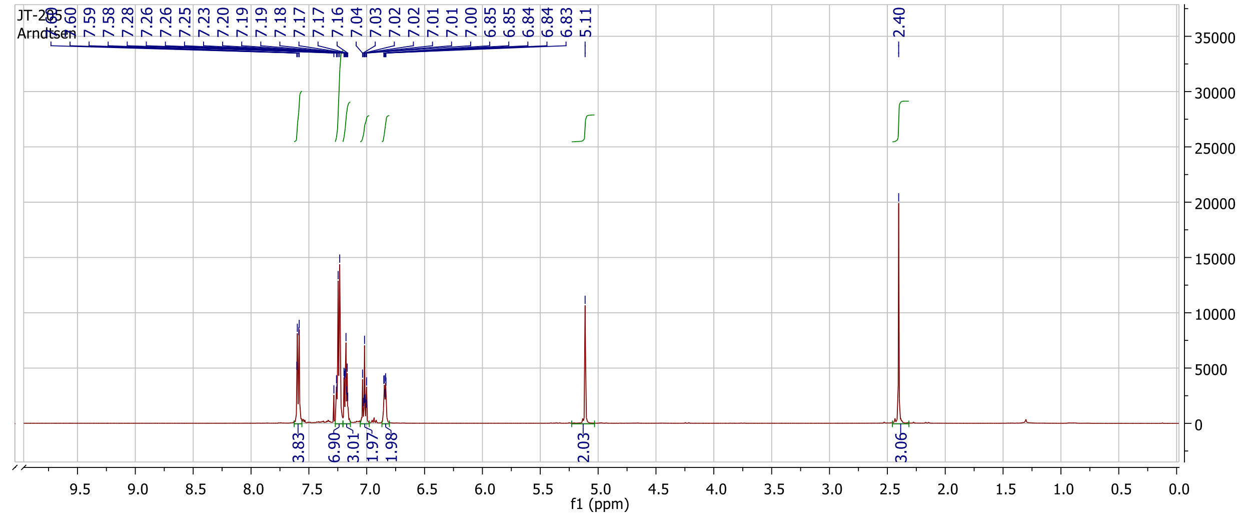

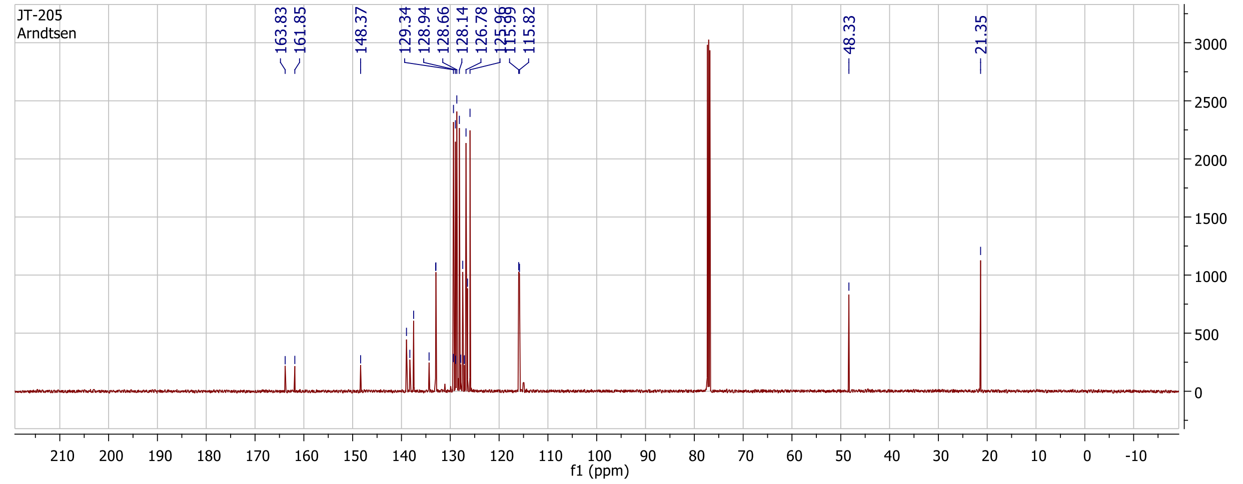

^{1}H and ^{13}C NMR spectra of 1c

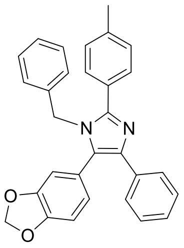

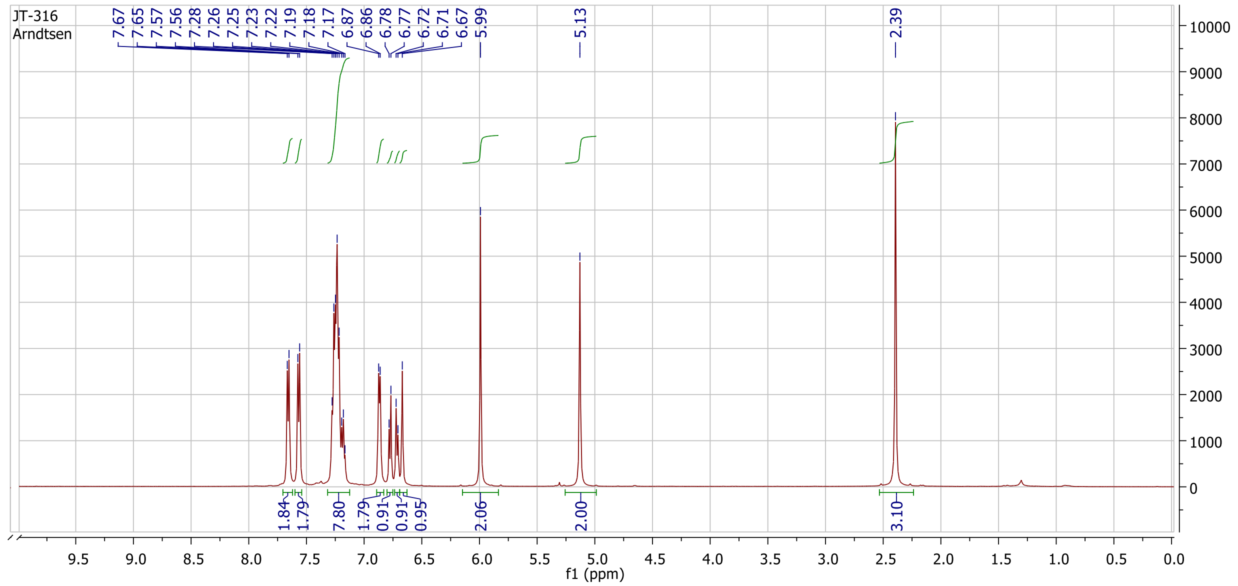

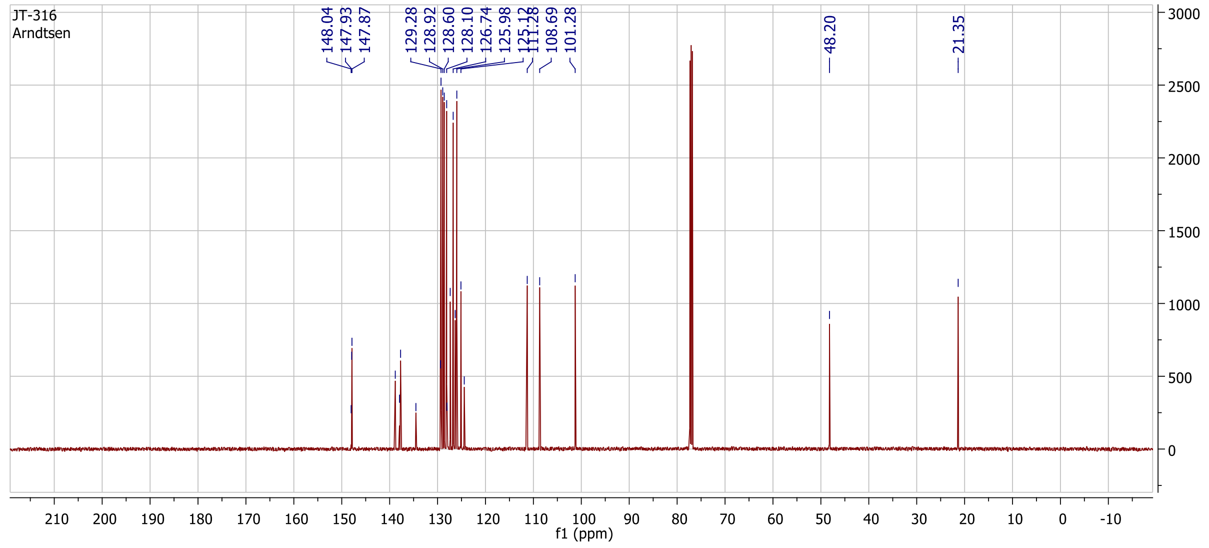

^{1}H and ^{13}C NMR spectra of 1d

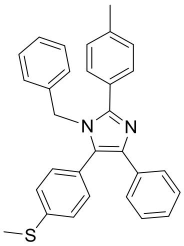

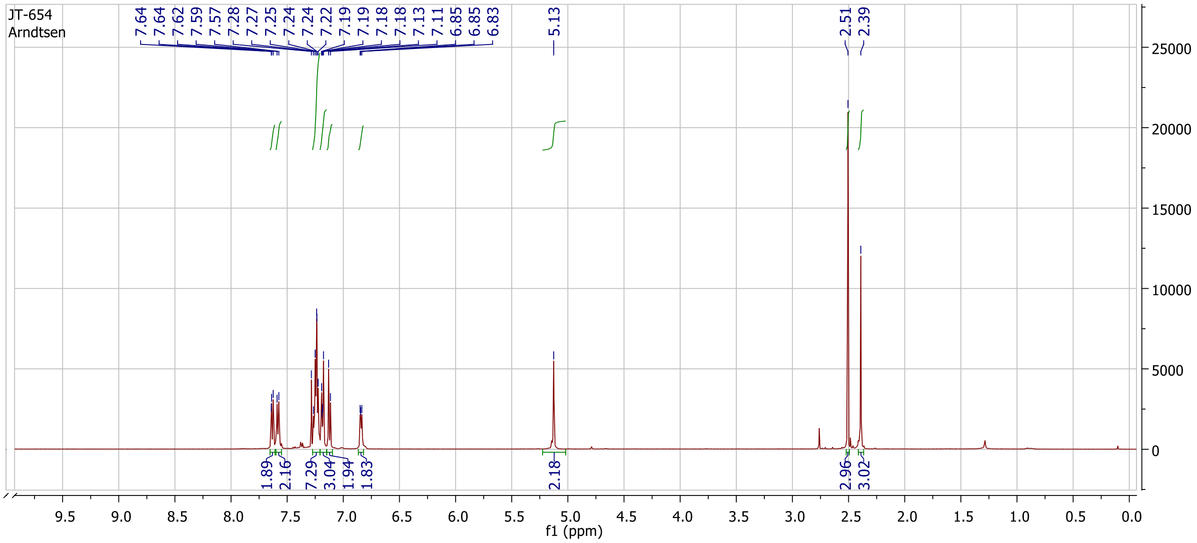

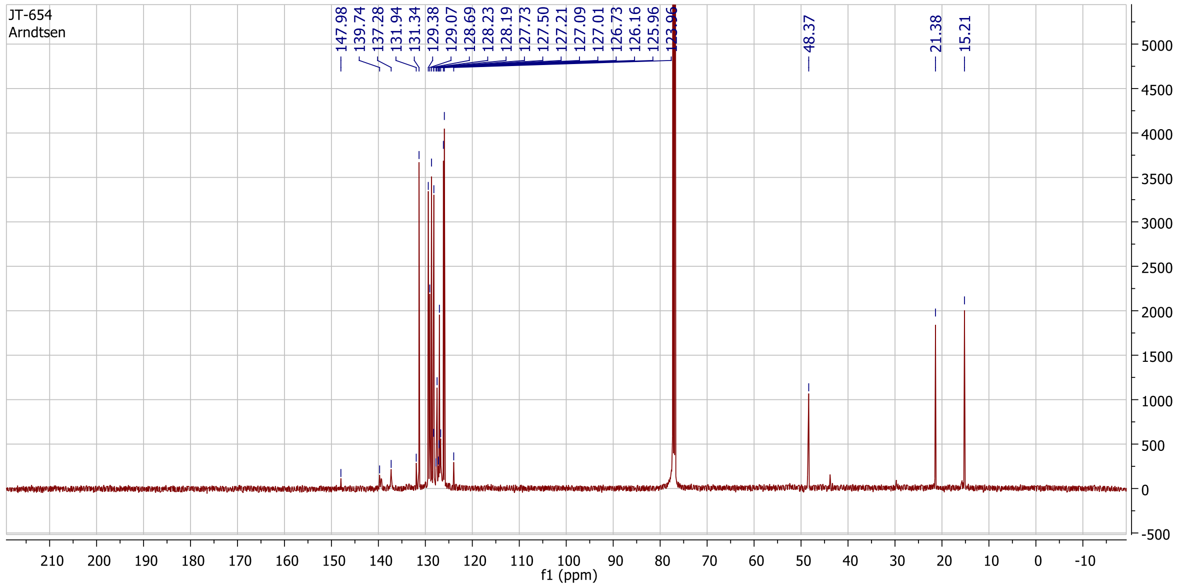

^{1}H and ^{13}C NMR spectra of 1e

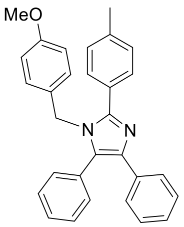

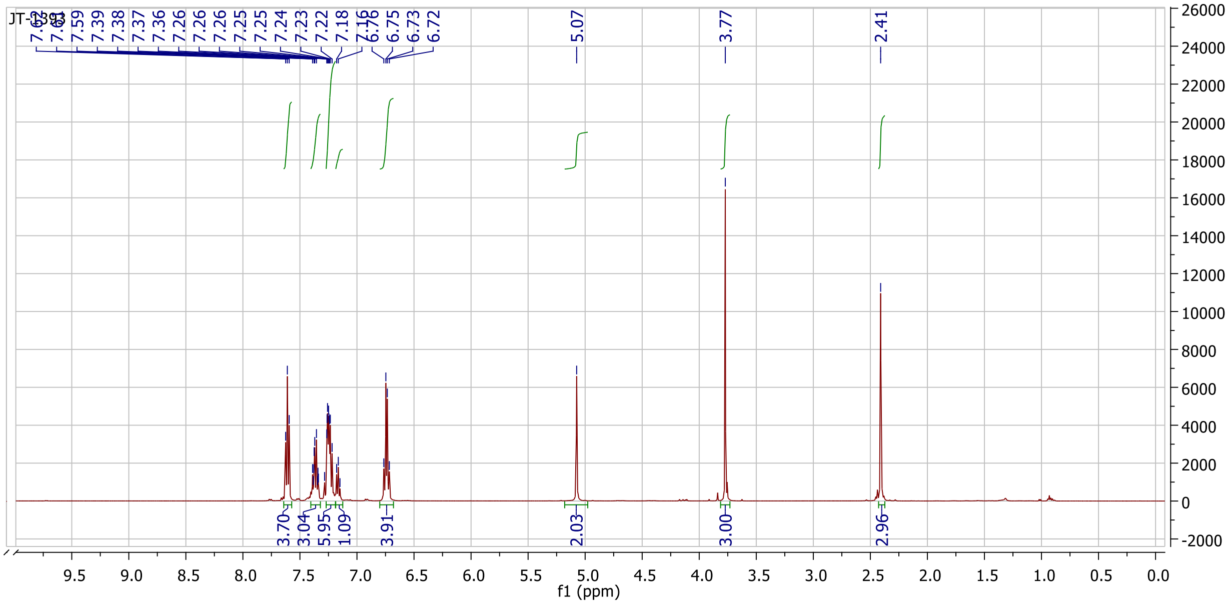

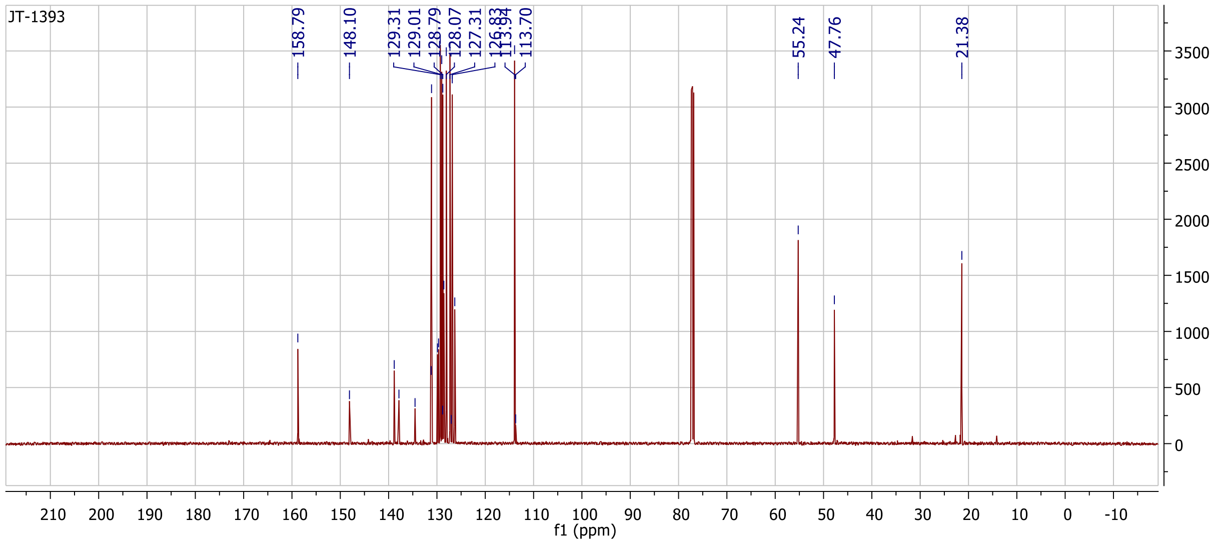

^{1}H and ^{13}C NMR spectra of 1f

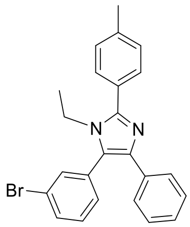

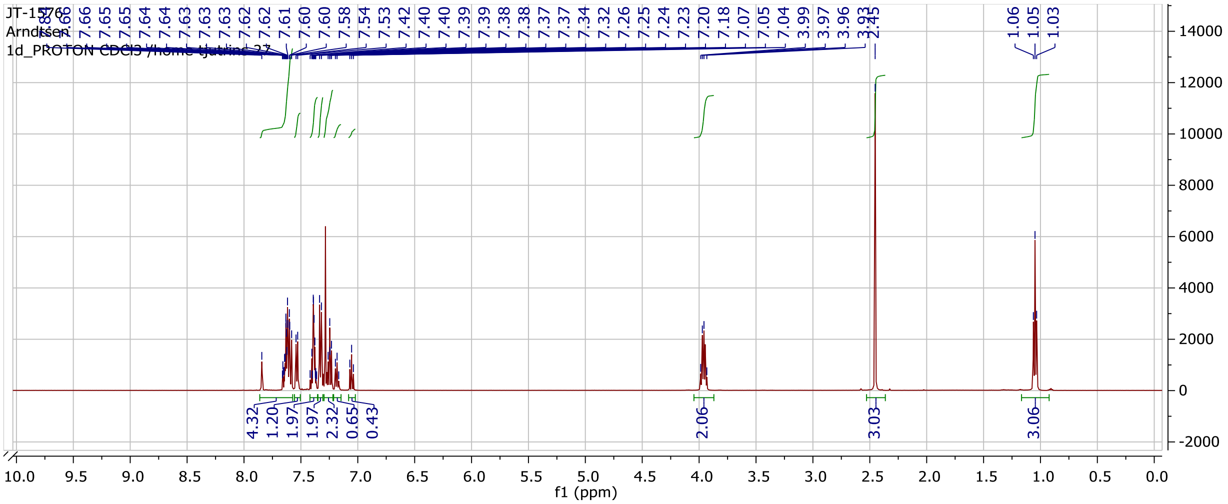

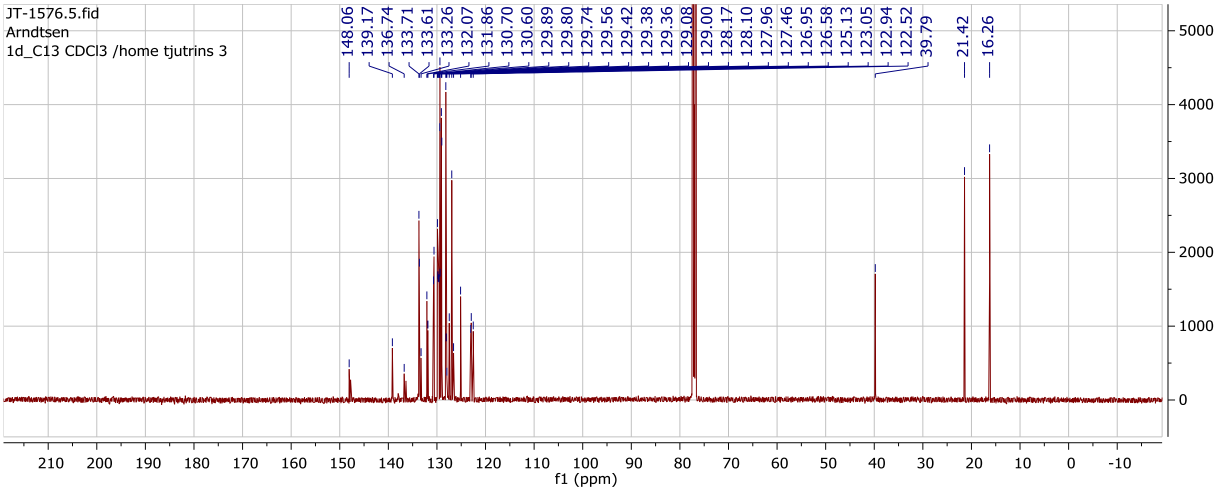

^{1}H and ^{13}C NMR spectra of 1g

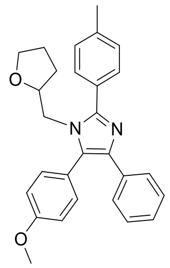

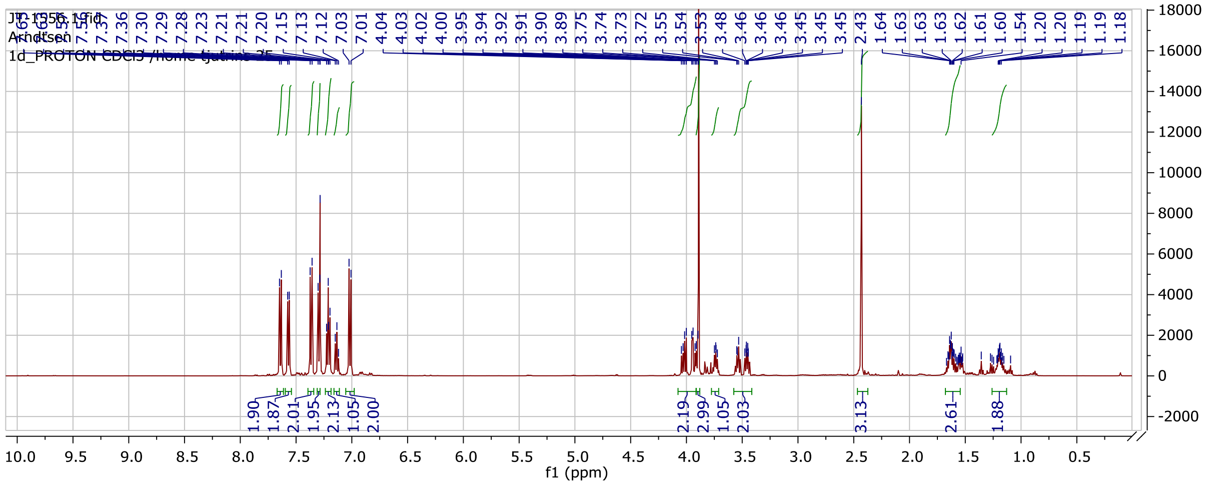

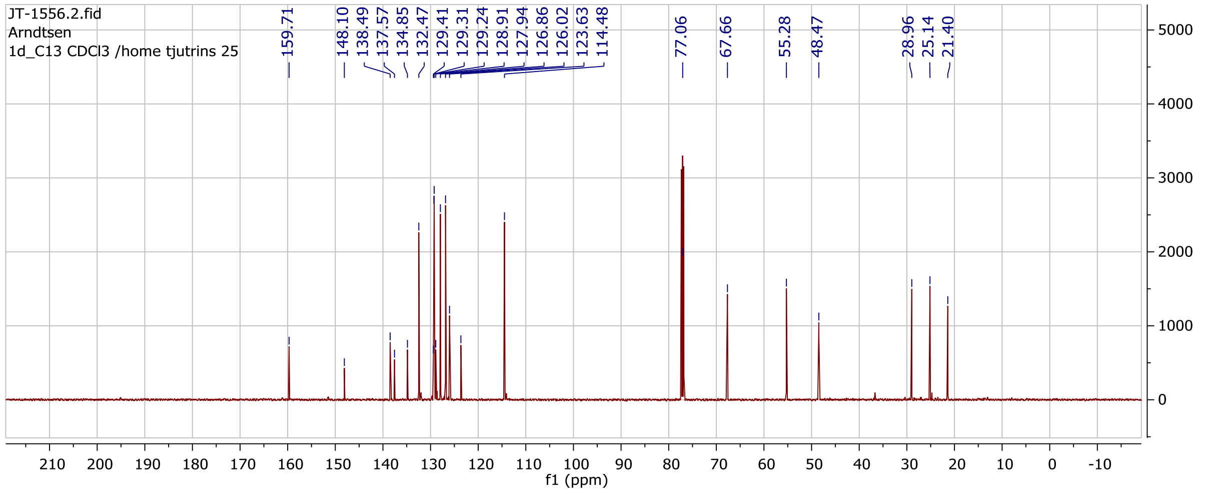

^{1}H and ^{13}C NMR spectra of 1h

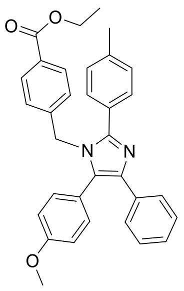

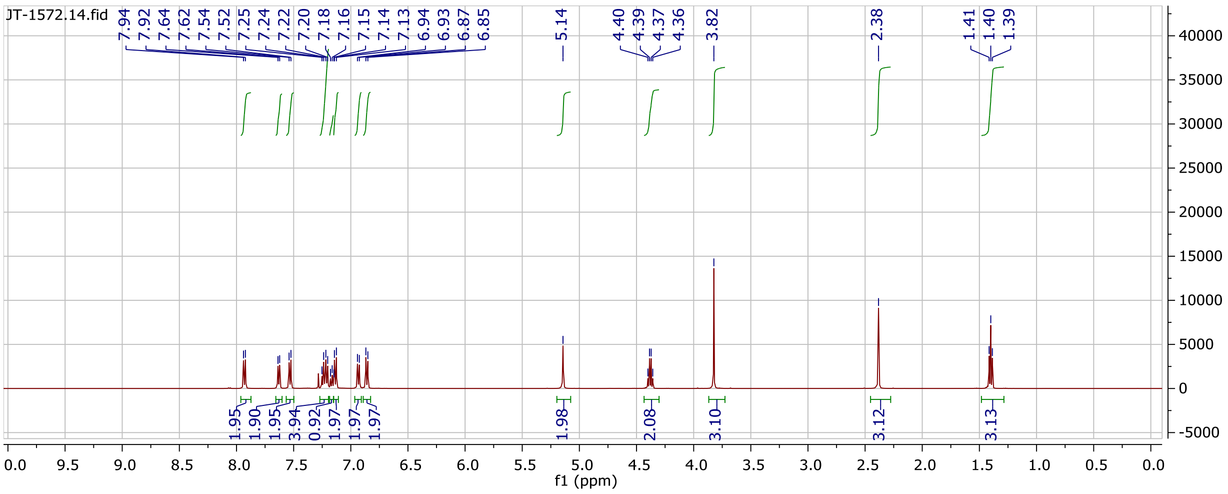

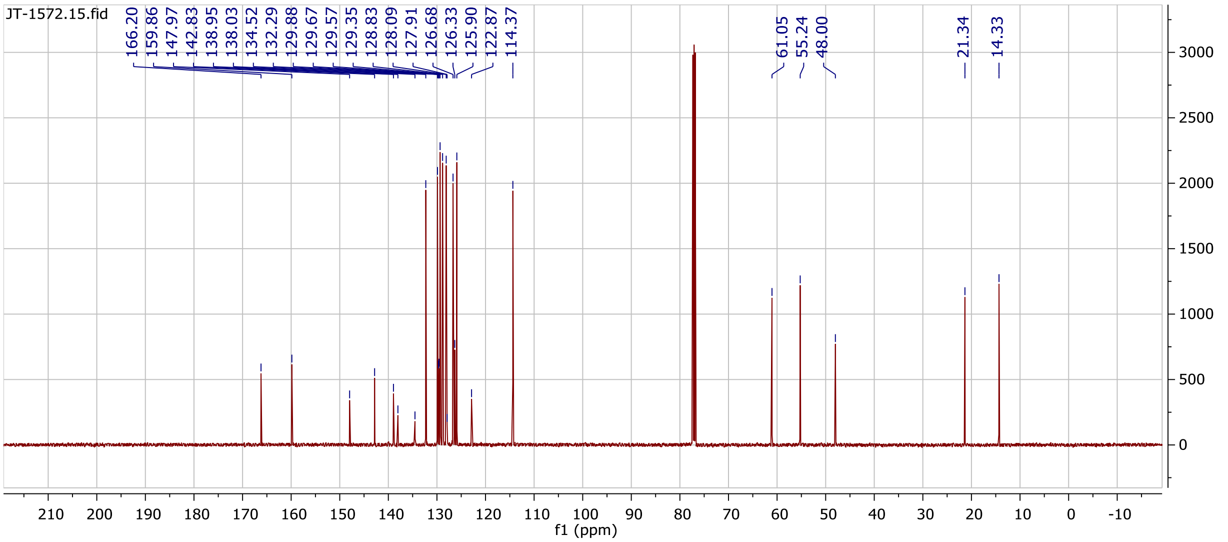

^{1}H and ^{13}C NMR spectra of 1i

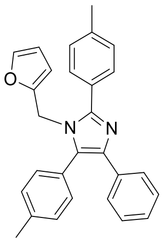

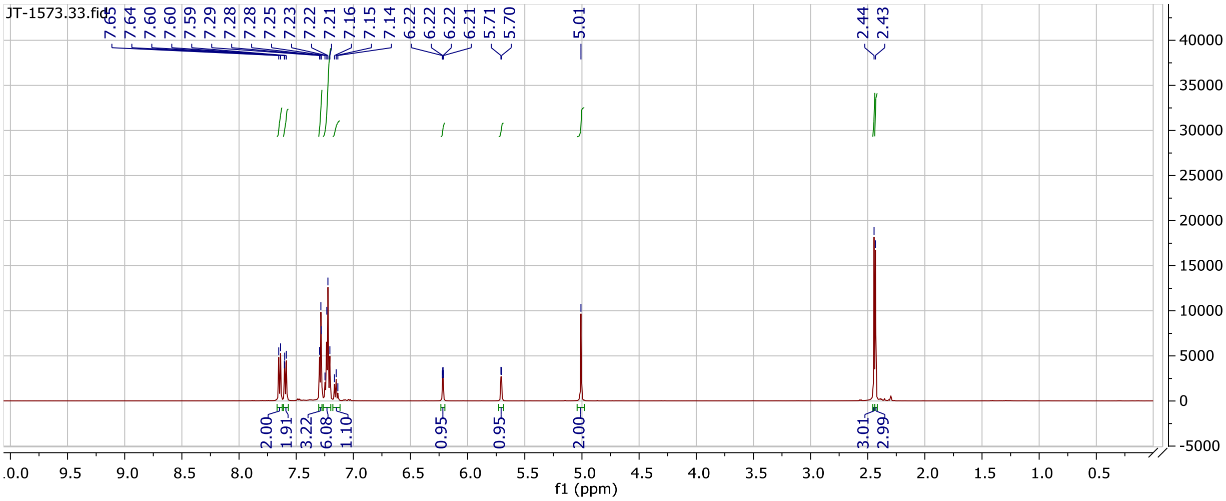

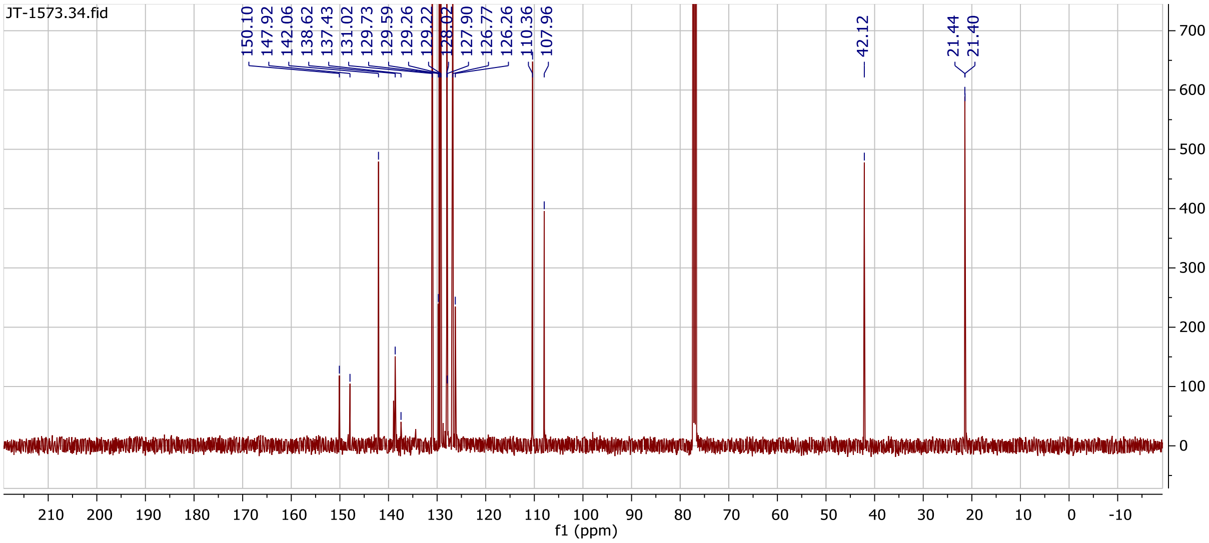

^{1}H and ^{13}C NMR spectra of 1j

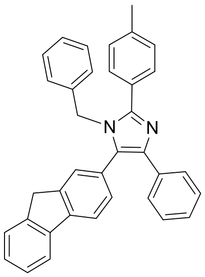

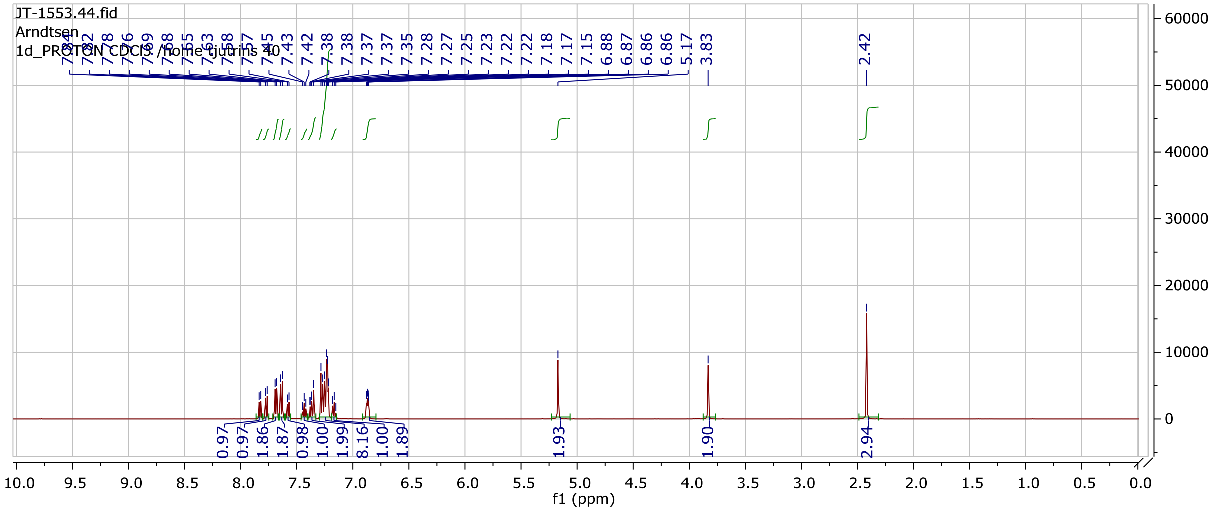

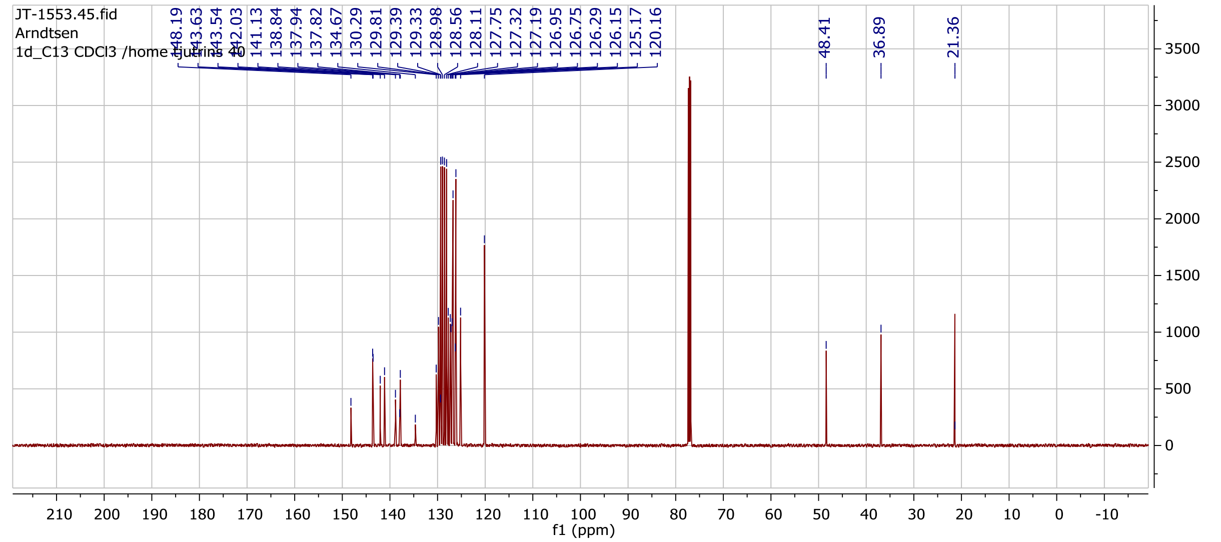

^{1}H and ^{13}C NMR spectra of 1k

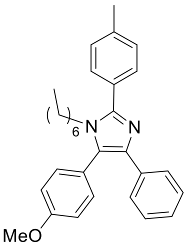

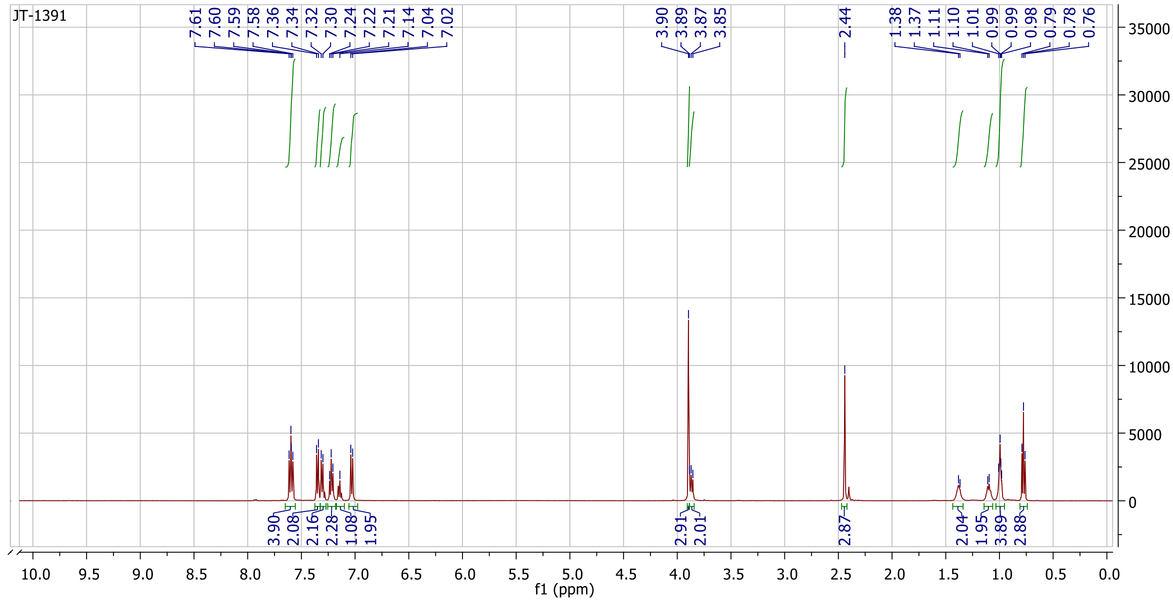

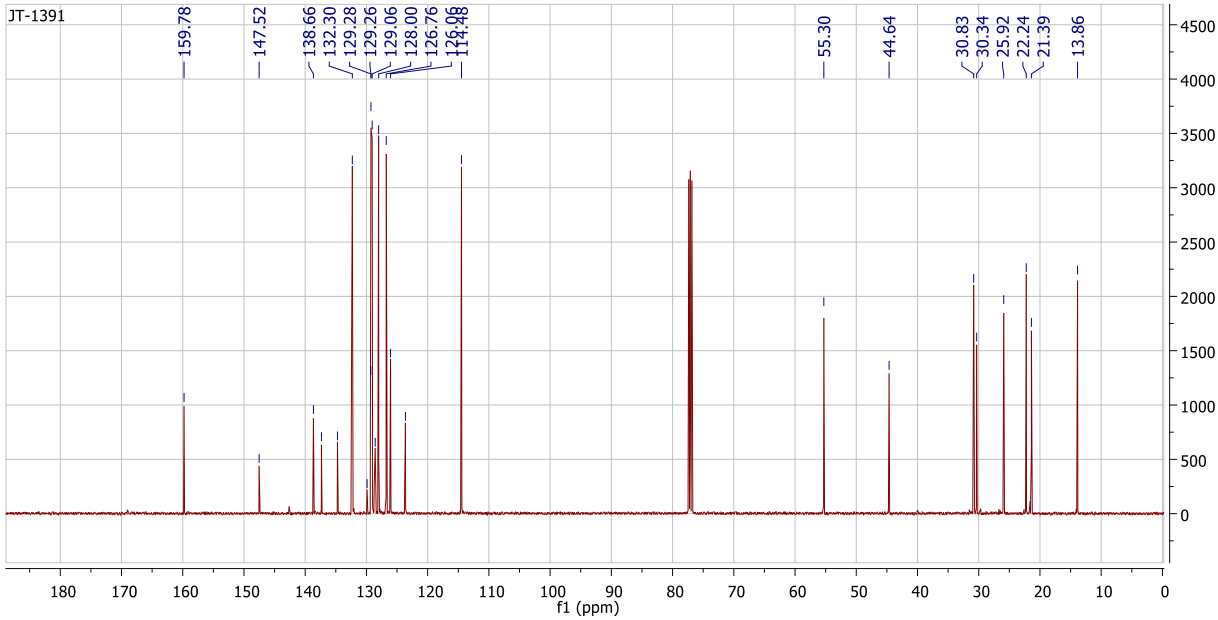

^{1}H and ^{13}C NMR spectra of 1l

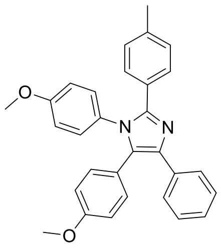

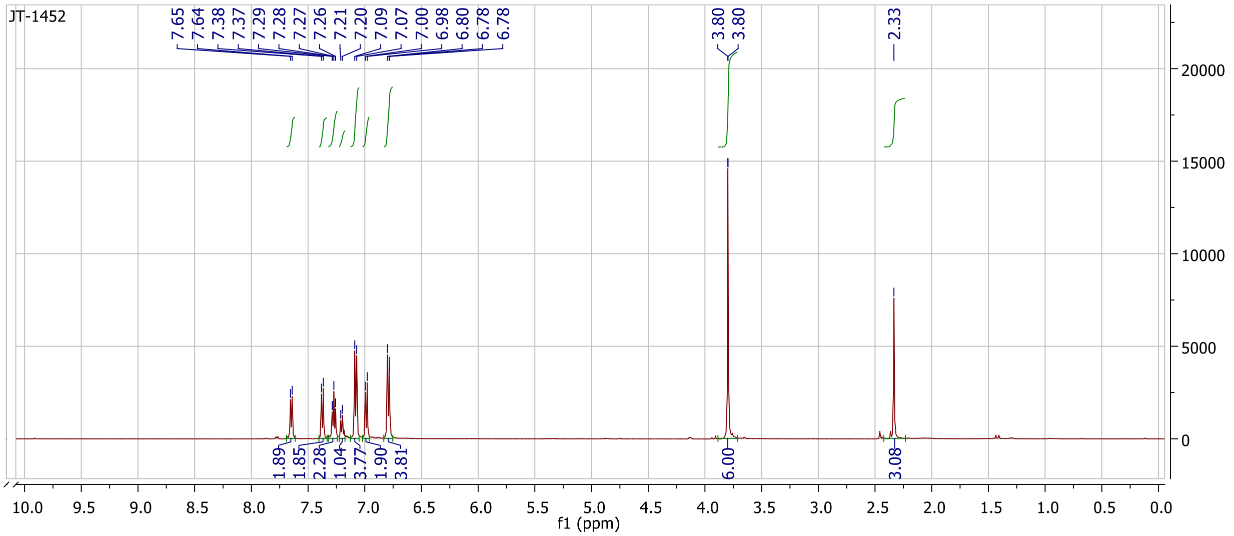

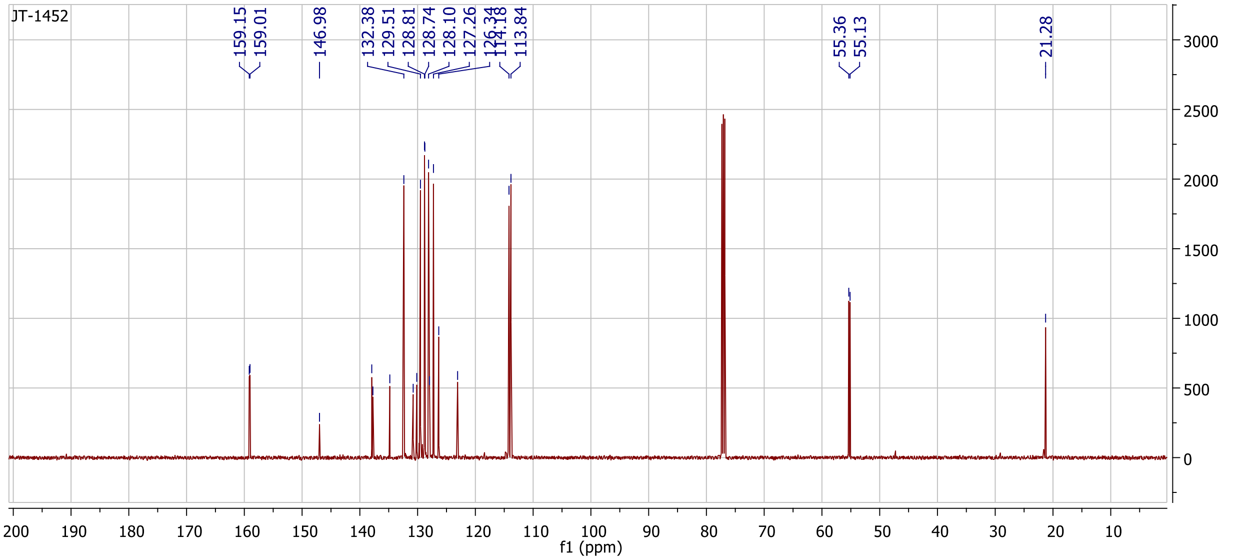

^{1}H and ^{13}C NMR spectra of 1l

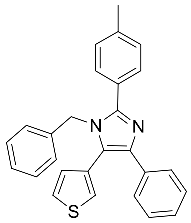

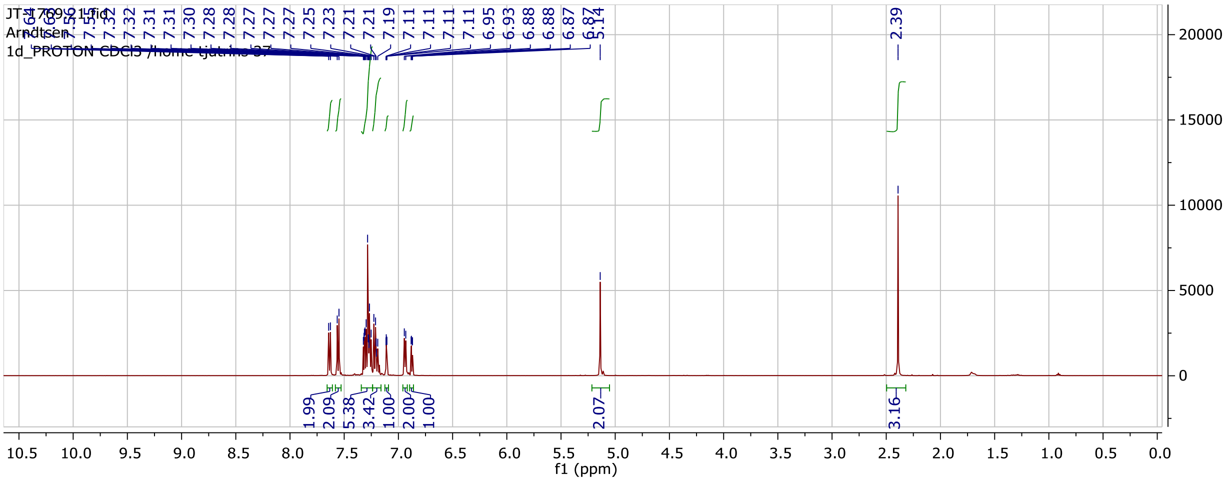

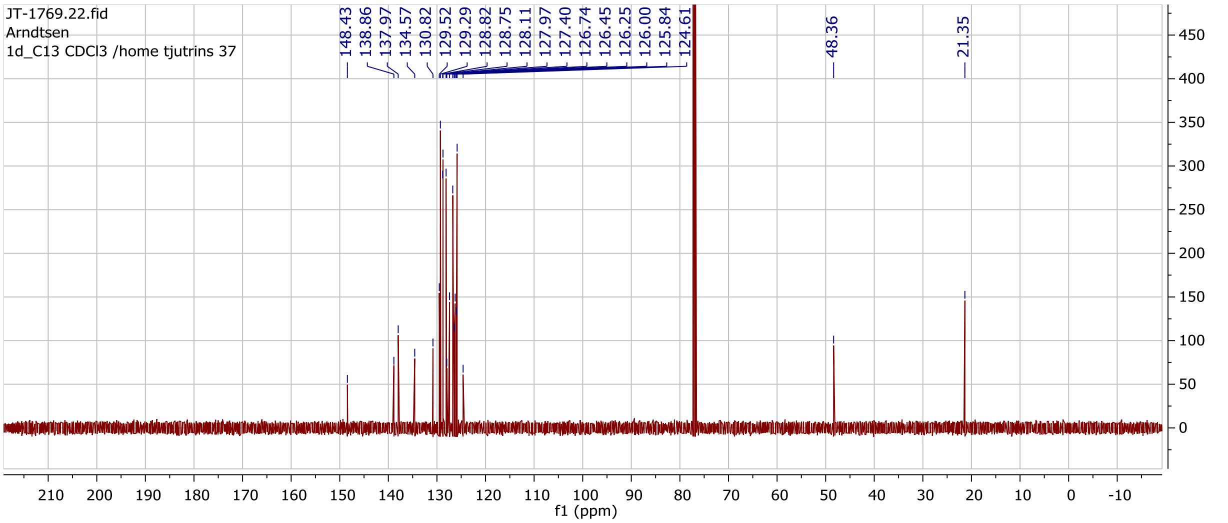

^{1}H and ^{13}C NMR spectra of 1n

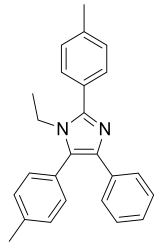

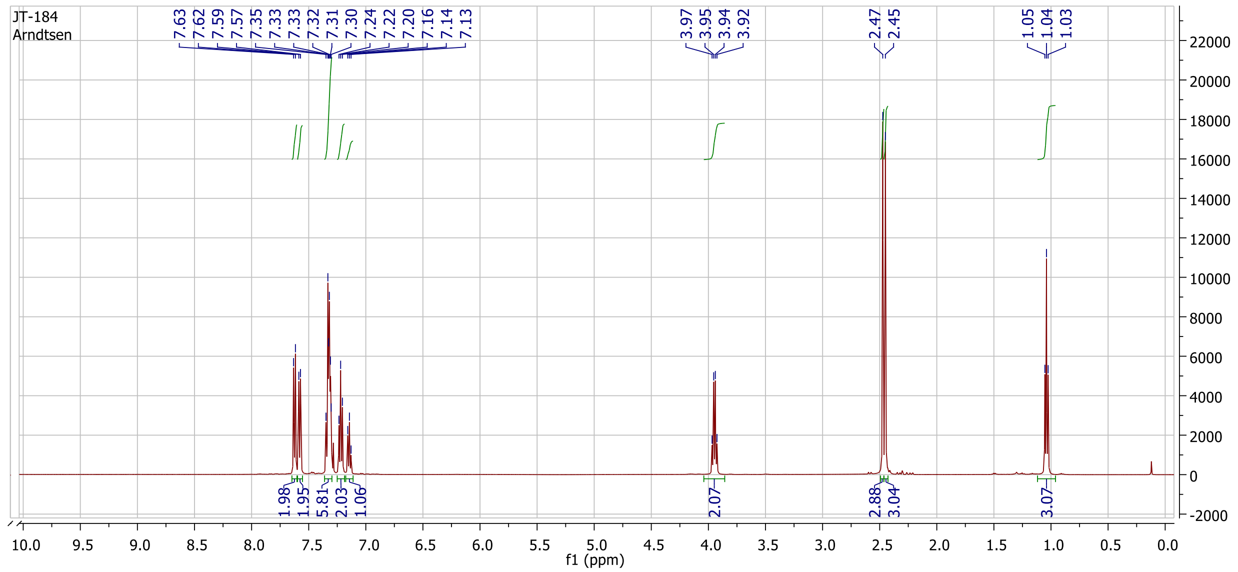

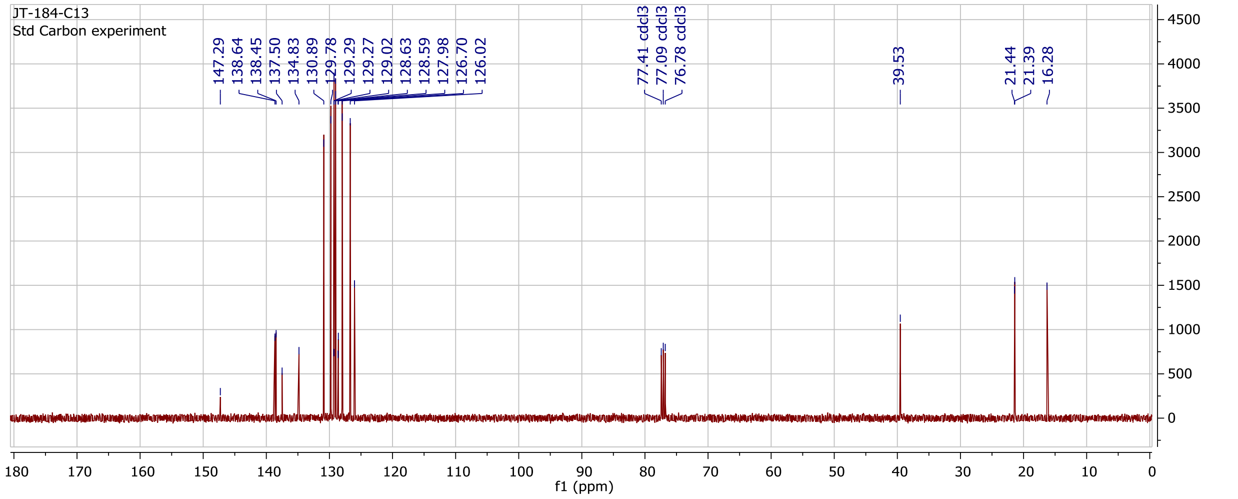

^{1}H and ^{13}C NMR spectra of 1o

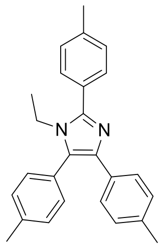

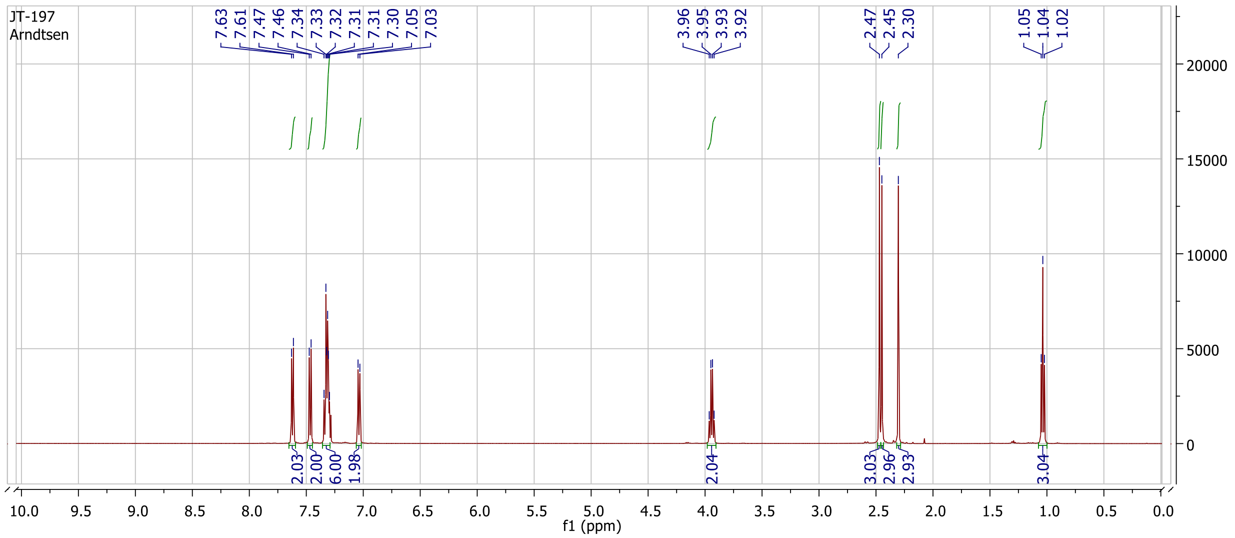

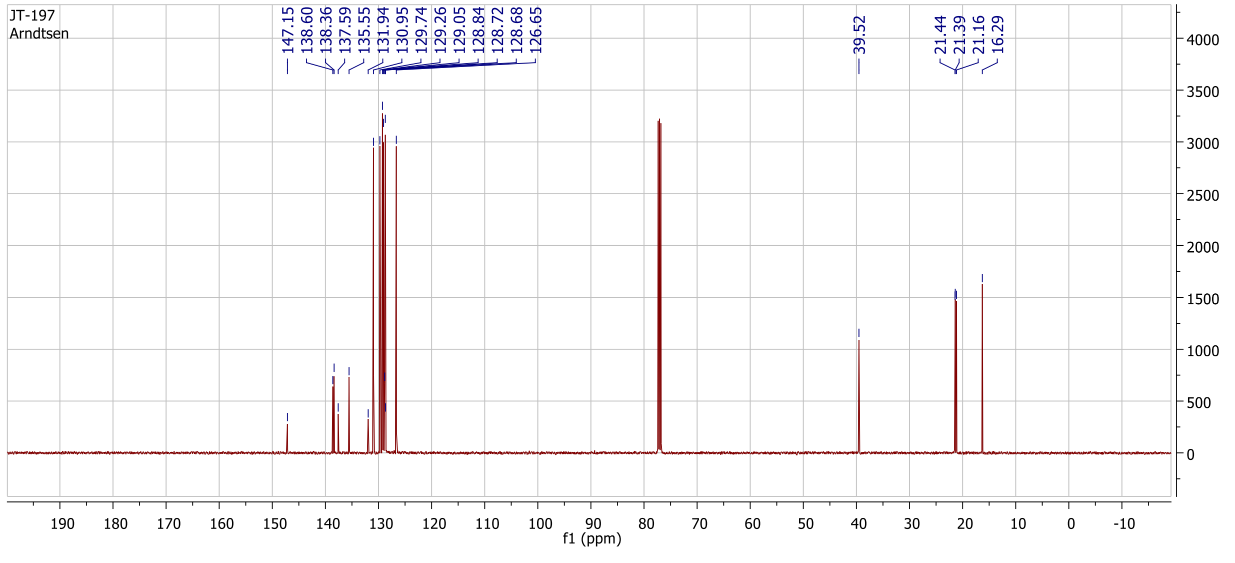

^{1}H and ^{13}C NMR spectra of 1p

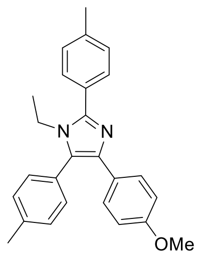

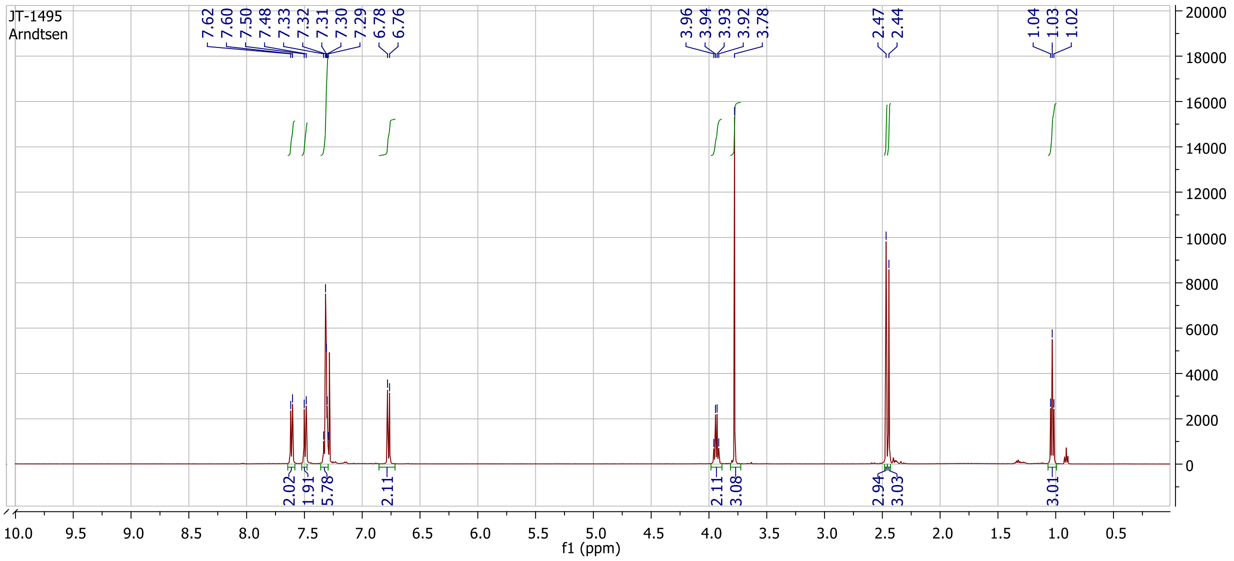

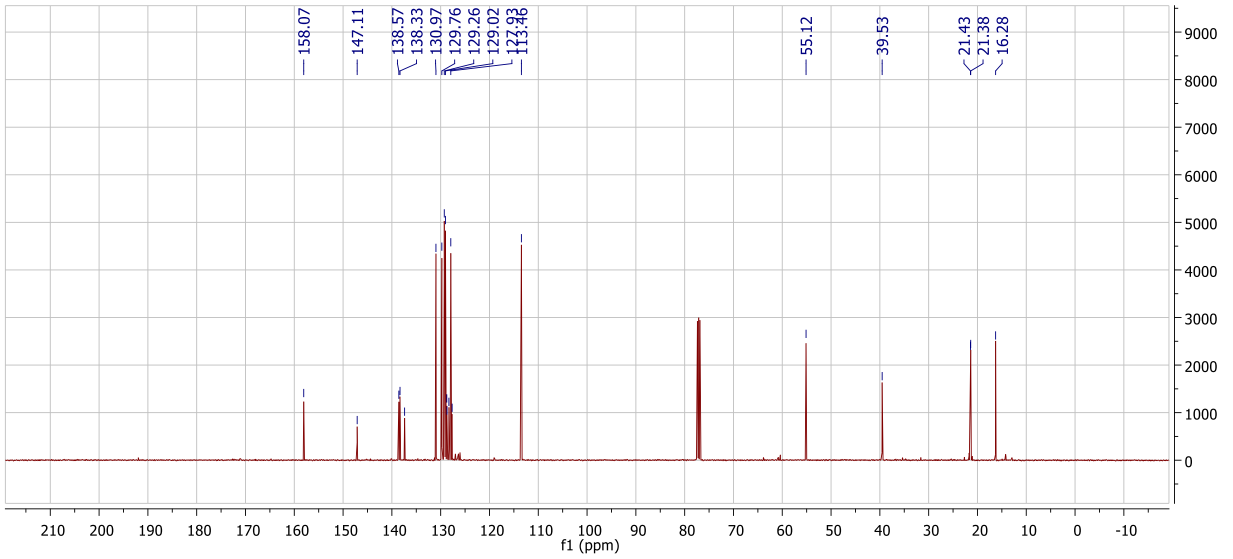

^{1}H and ^{13}C NMR spectra of 1q

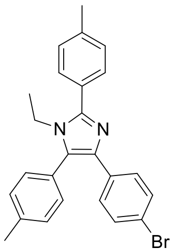

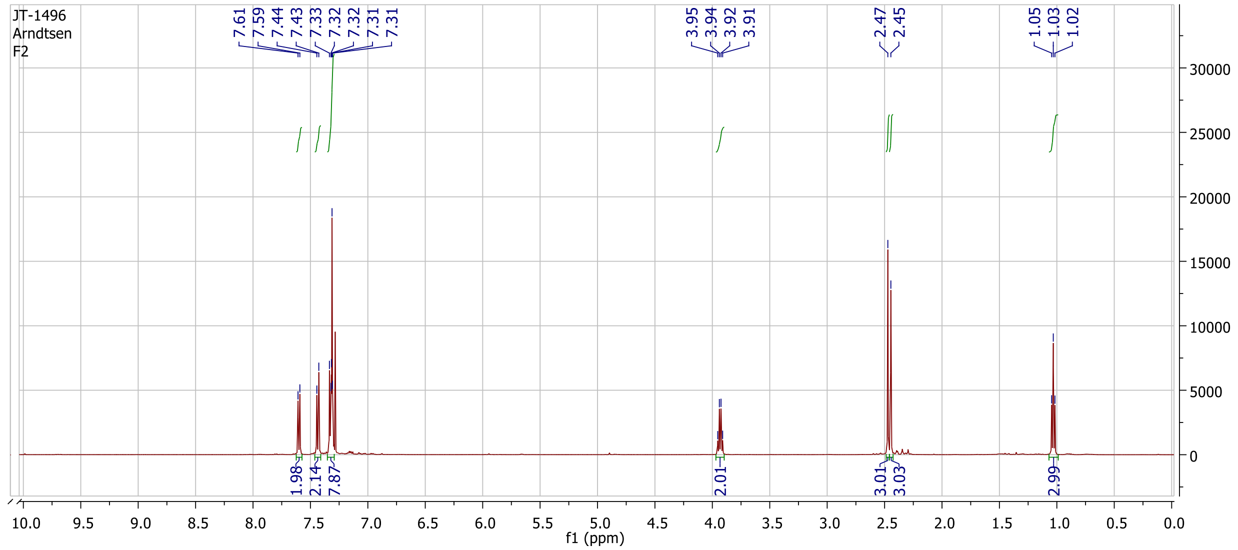

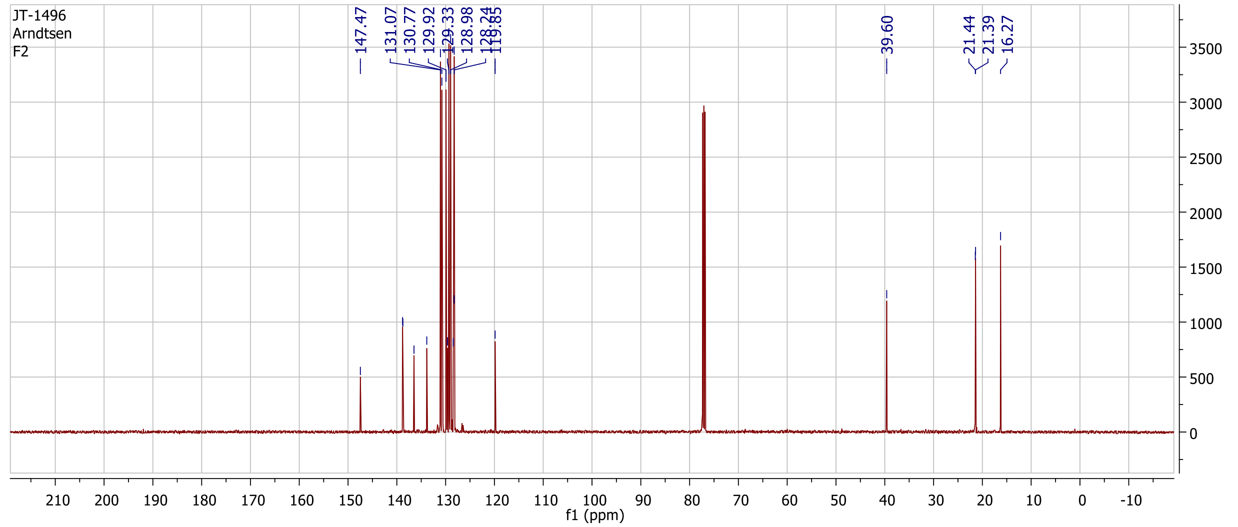

^{1}H and ^{13}C NMR spectra of 1r

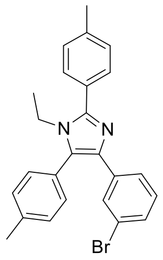

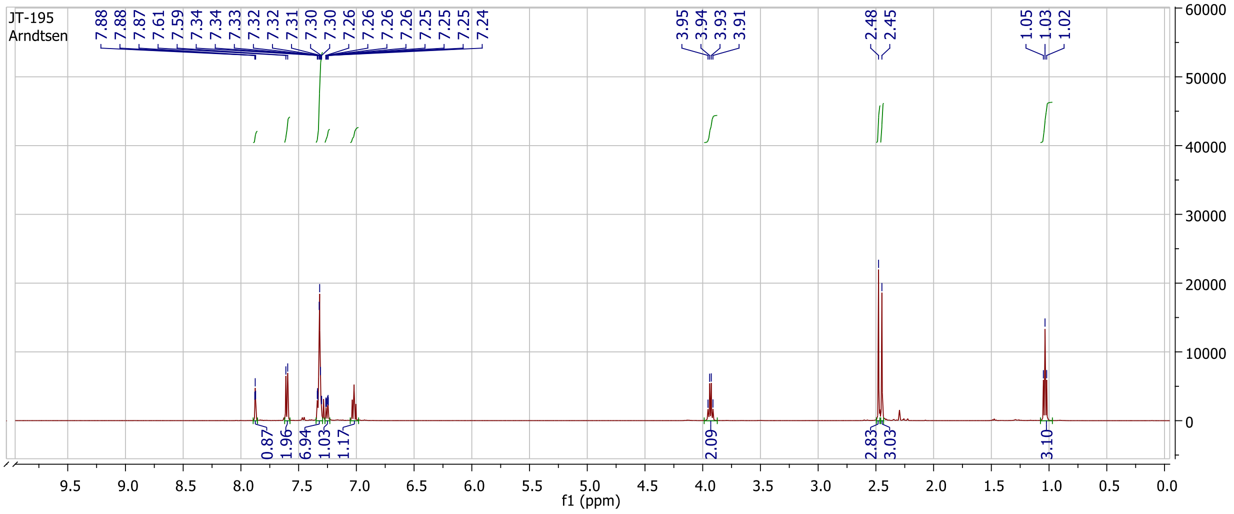

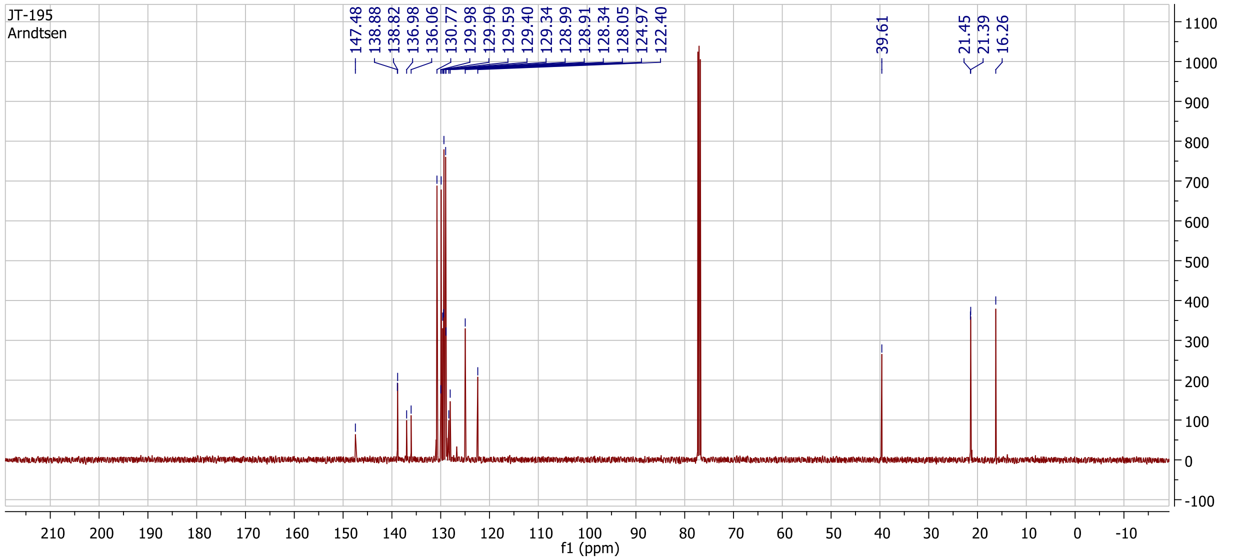

^{1}H and ^{13}C NMR spectra of 1s

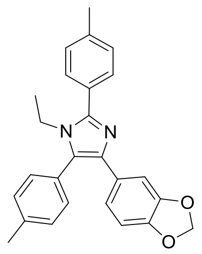

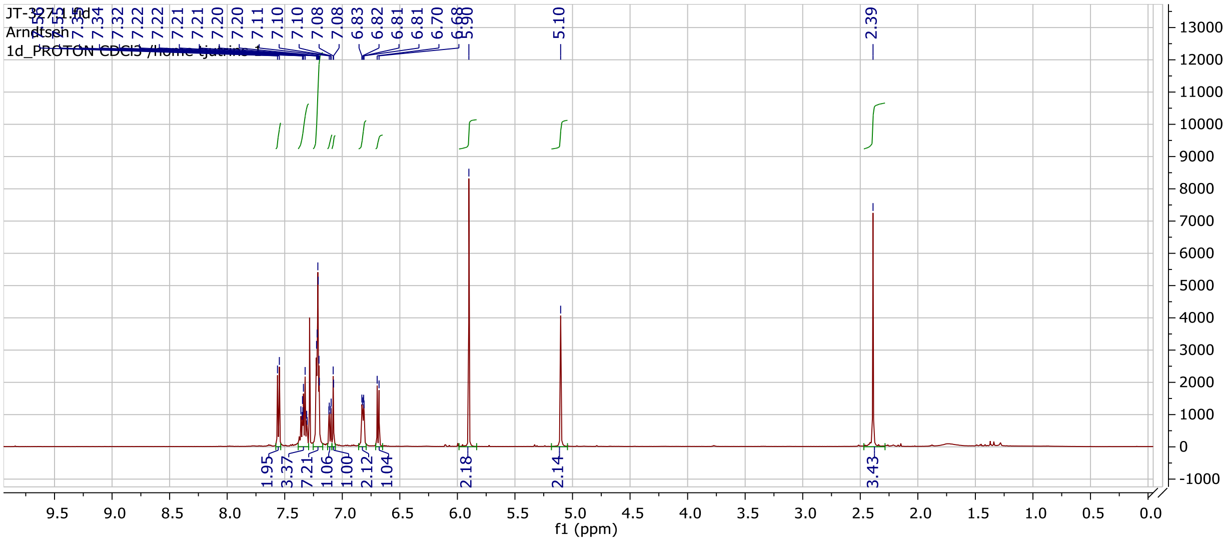

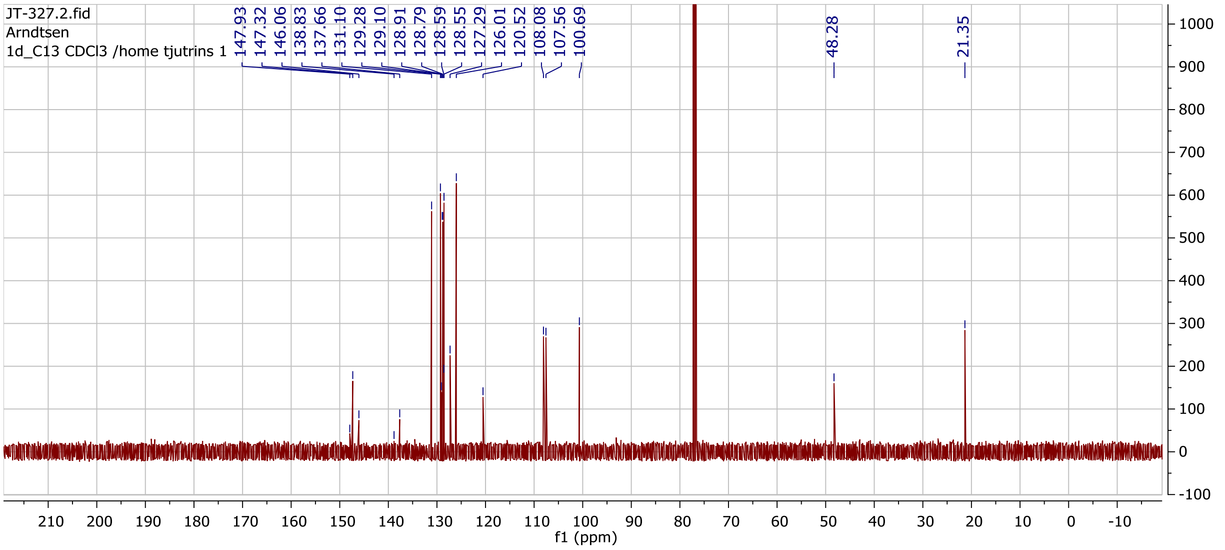

^{1}H and ^{13}C NMR spectra of 1t

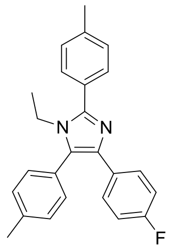

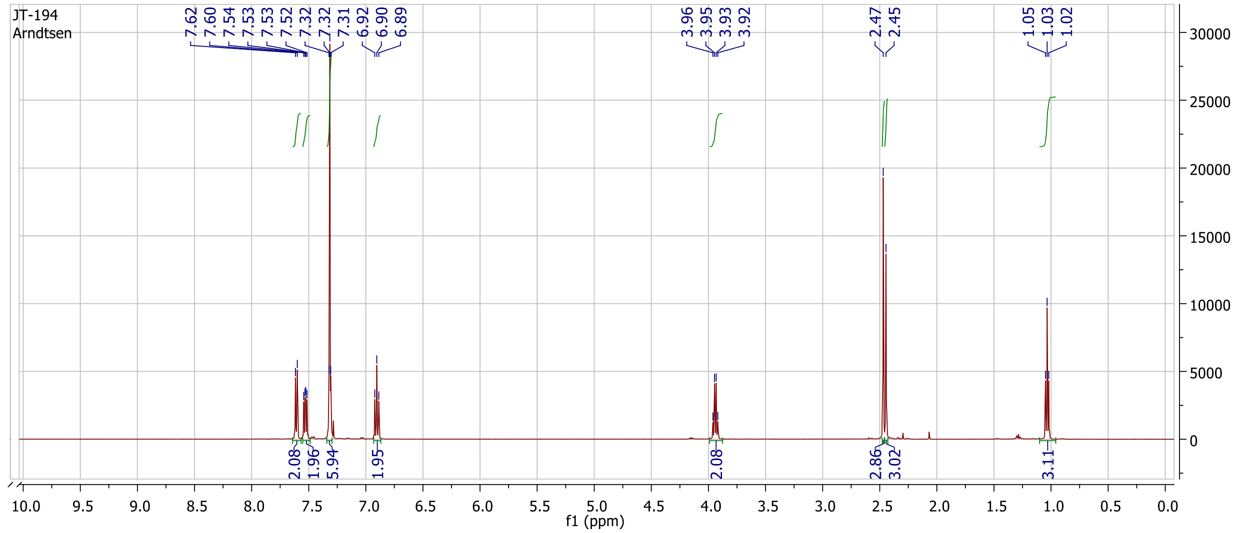

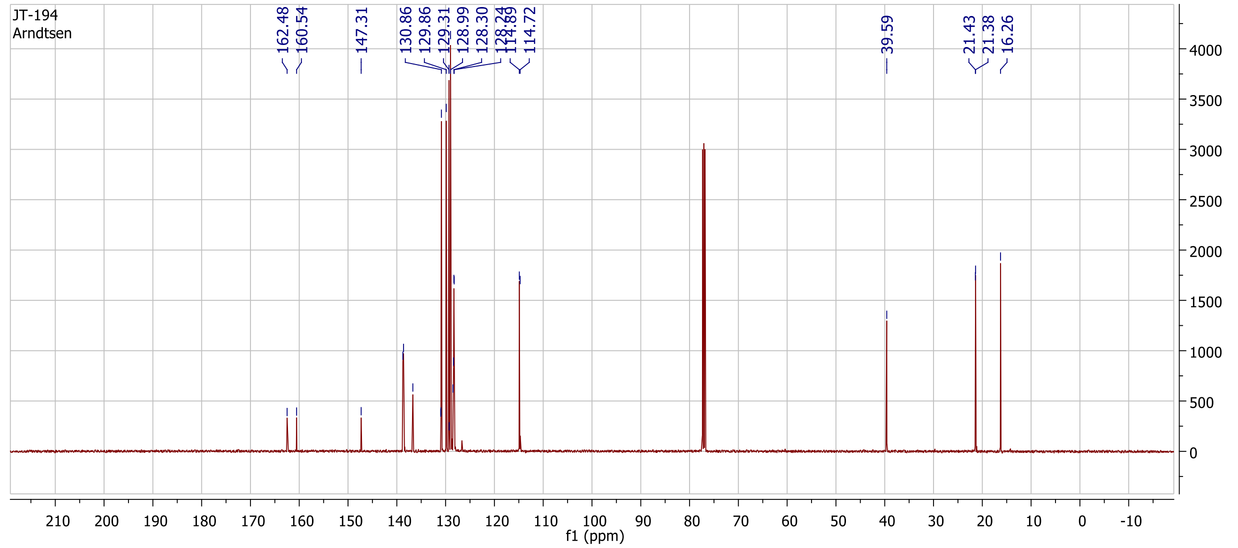

^{1}H and ^{13}C NMR spectra of 1u

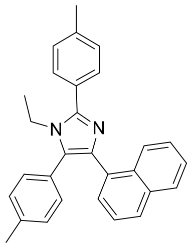

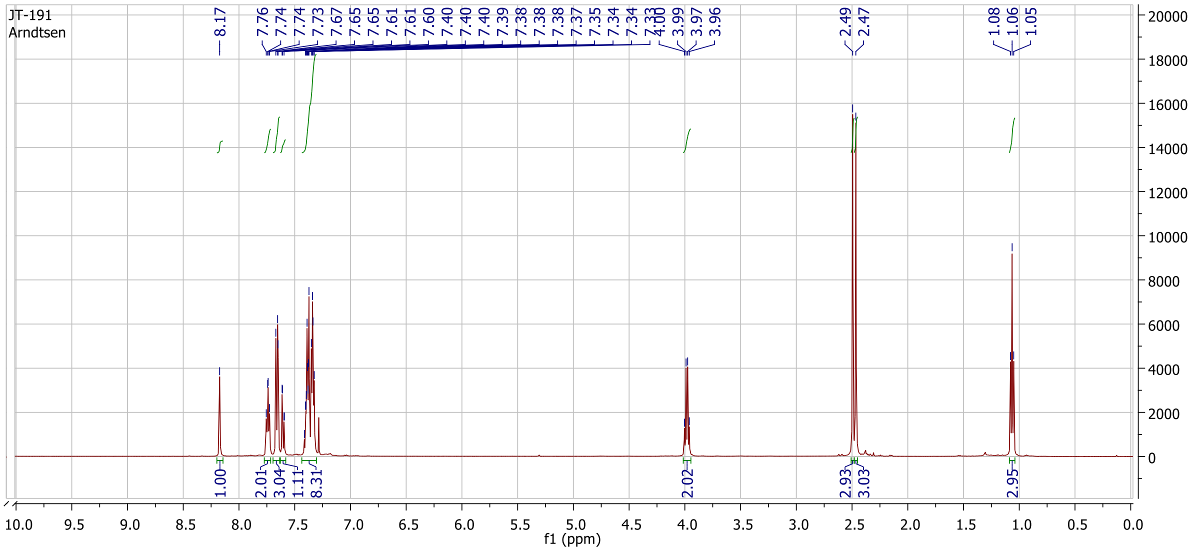

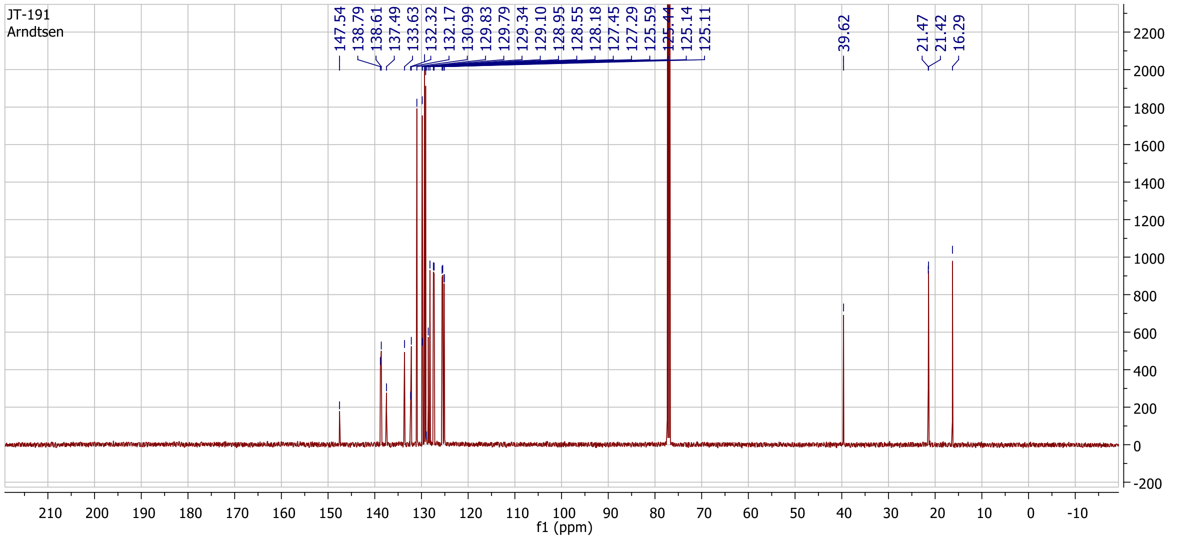

^{1}H and ^{13}C NMR spectra of 1v

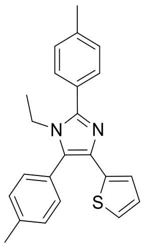

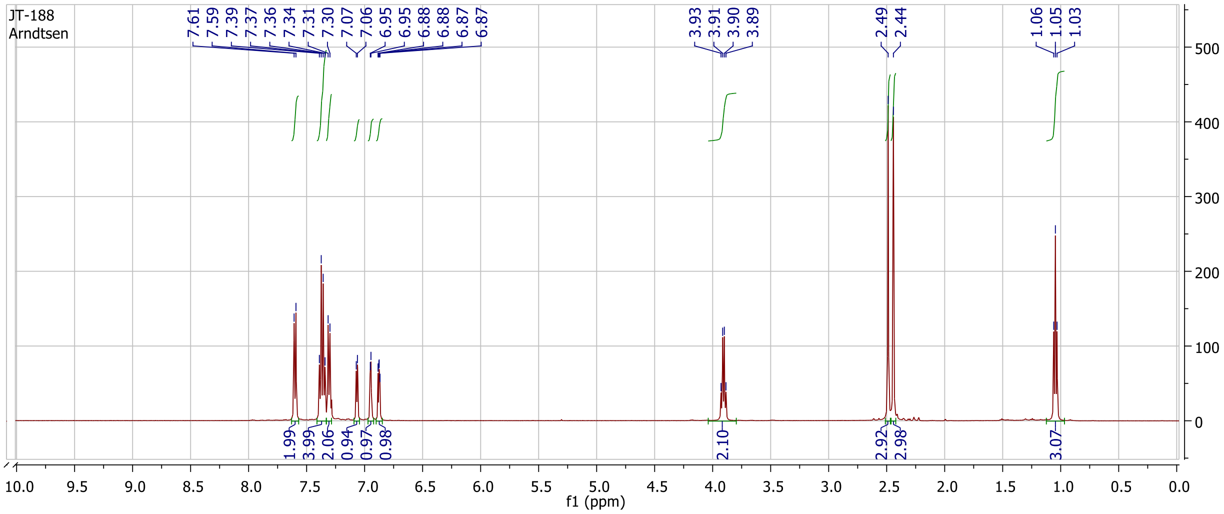

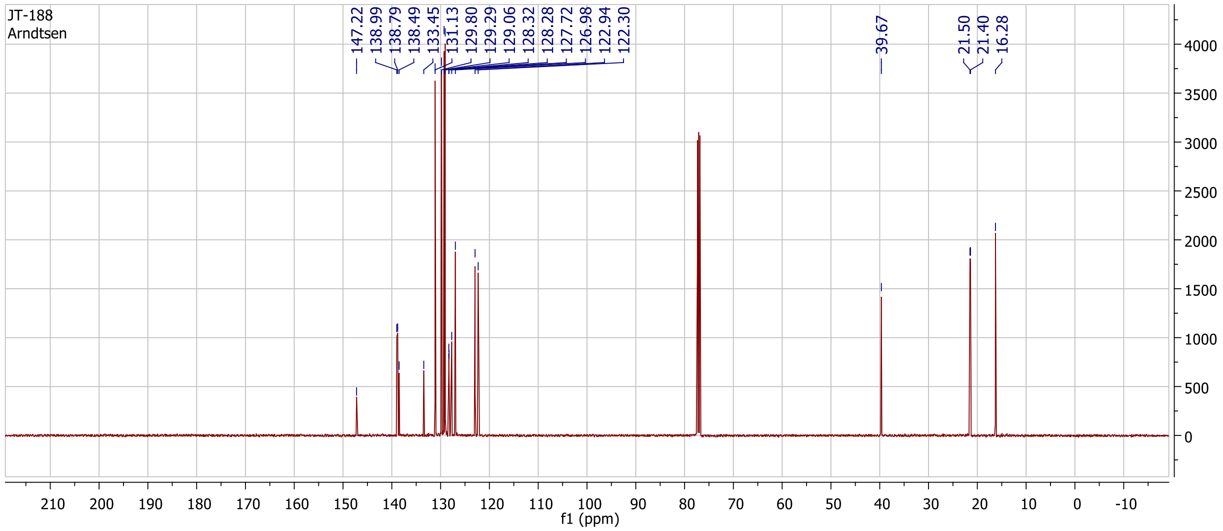

^{1}H and ^{13}C NMR spectra of 1w

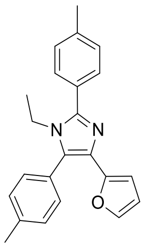

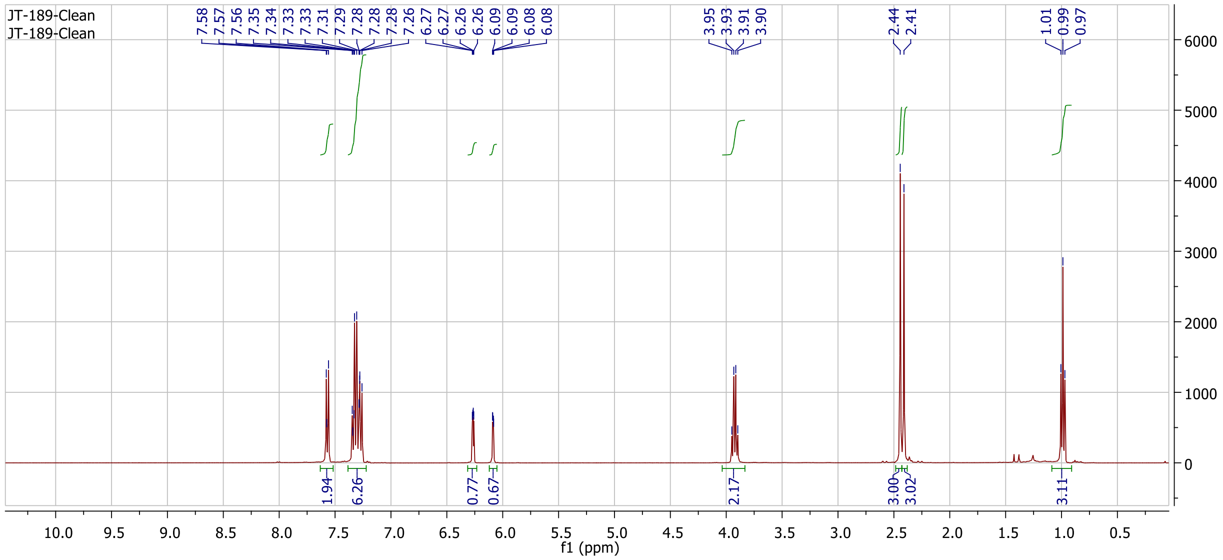

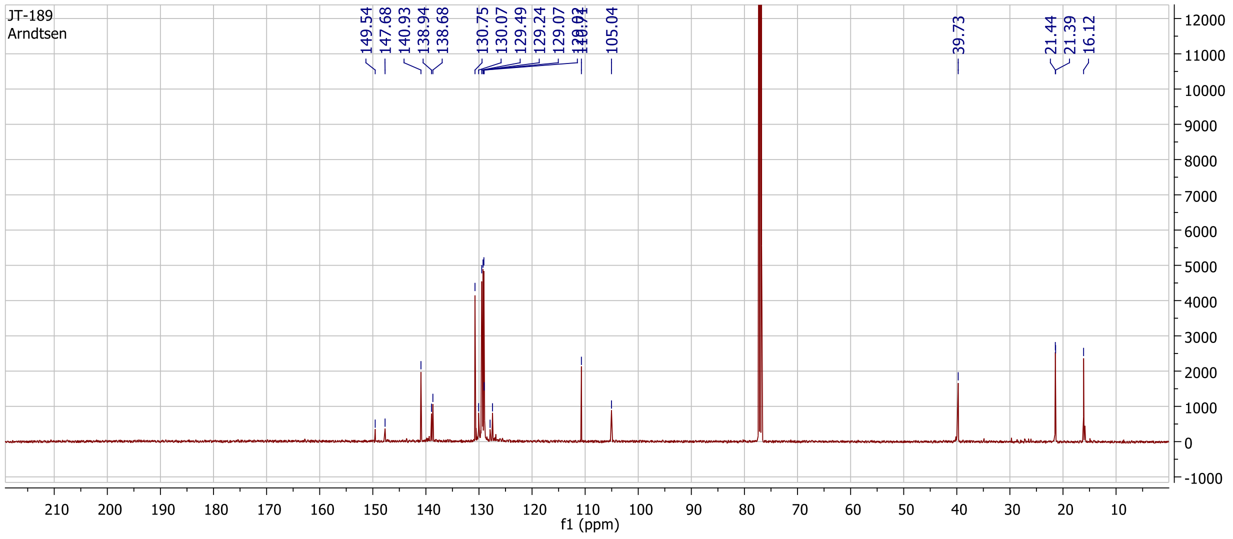

^{1}H and ^{13}C NMR spectra of 1x

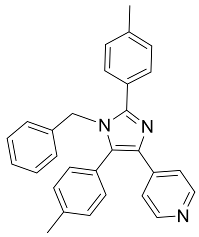

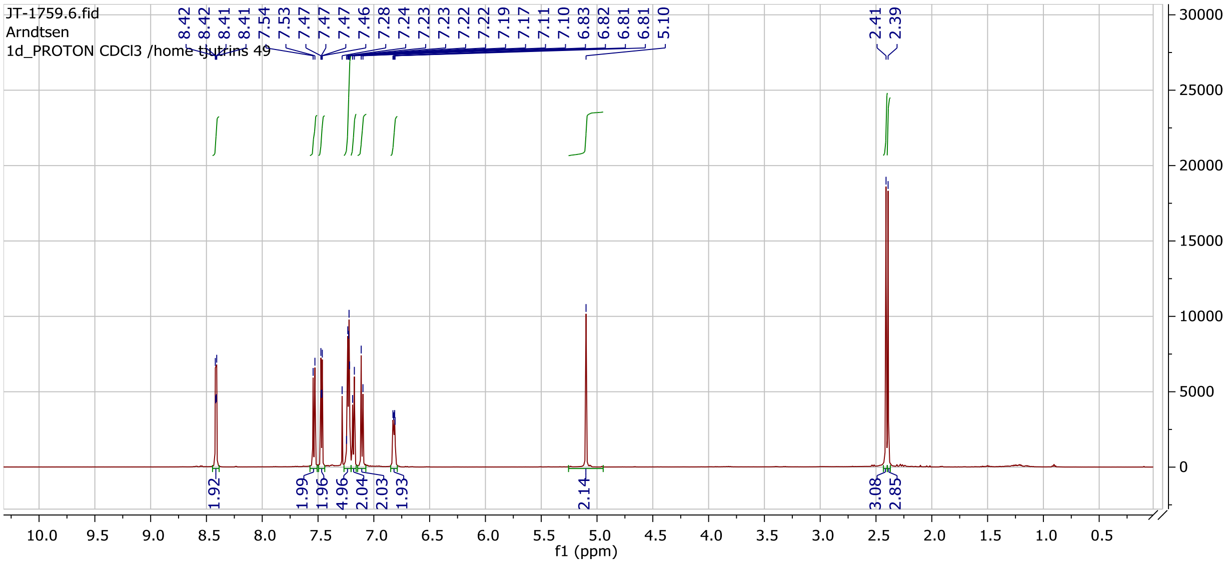

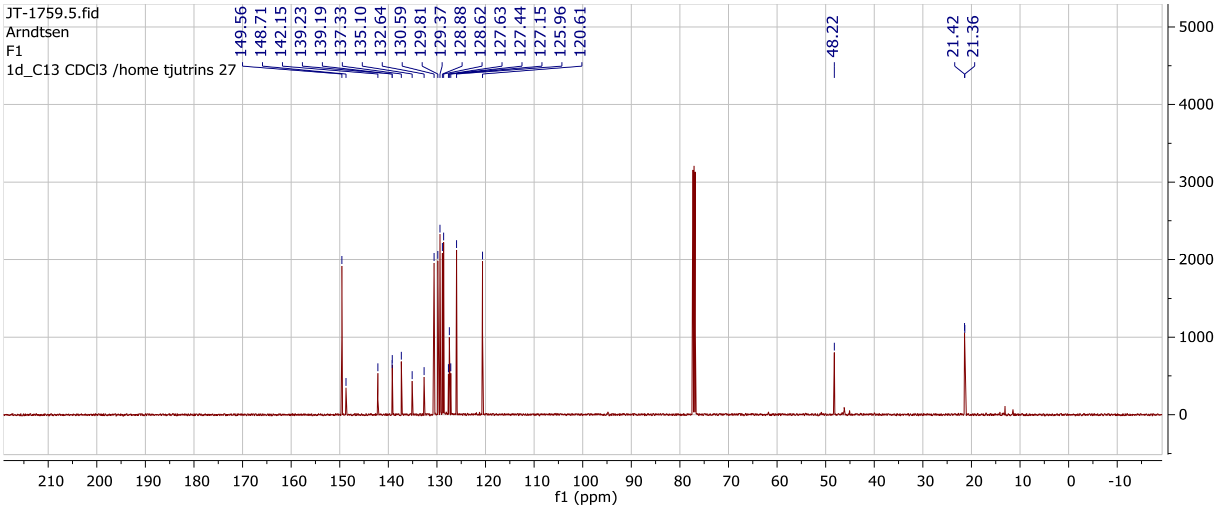

^{1}H and ^{13}C NMR spectra of 1y

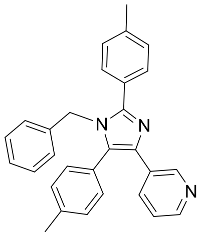

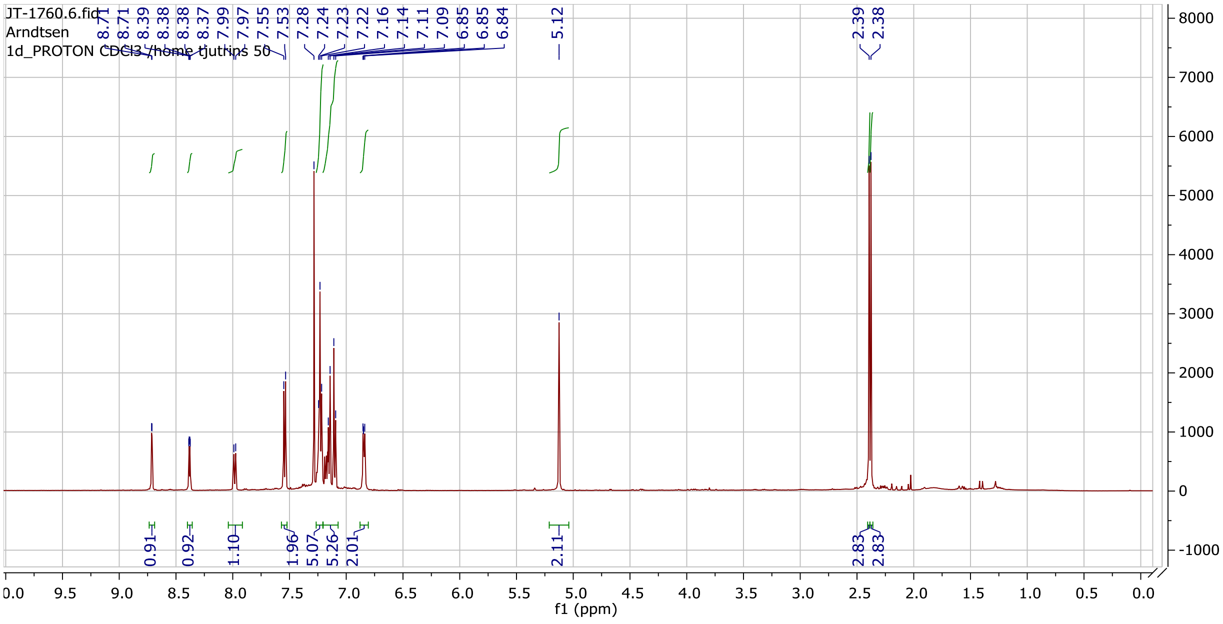

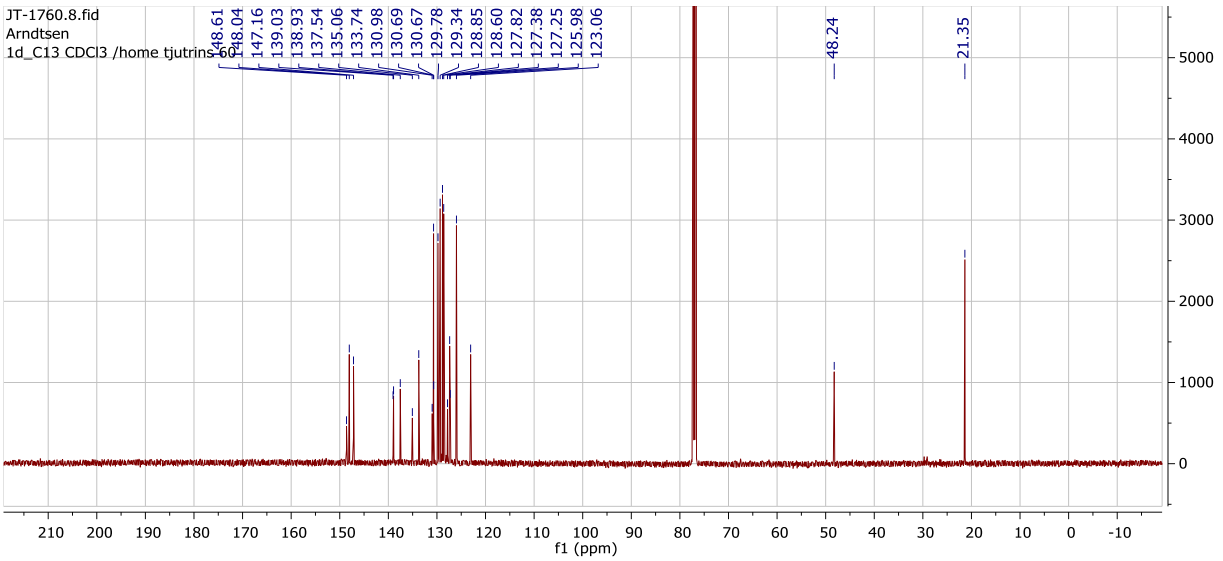

^{1}H and ^{13}C NMR spectra of 1z

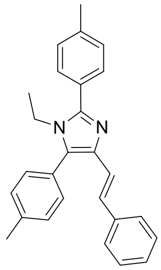

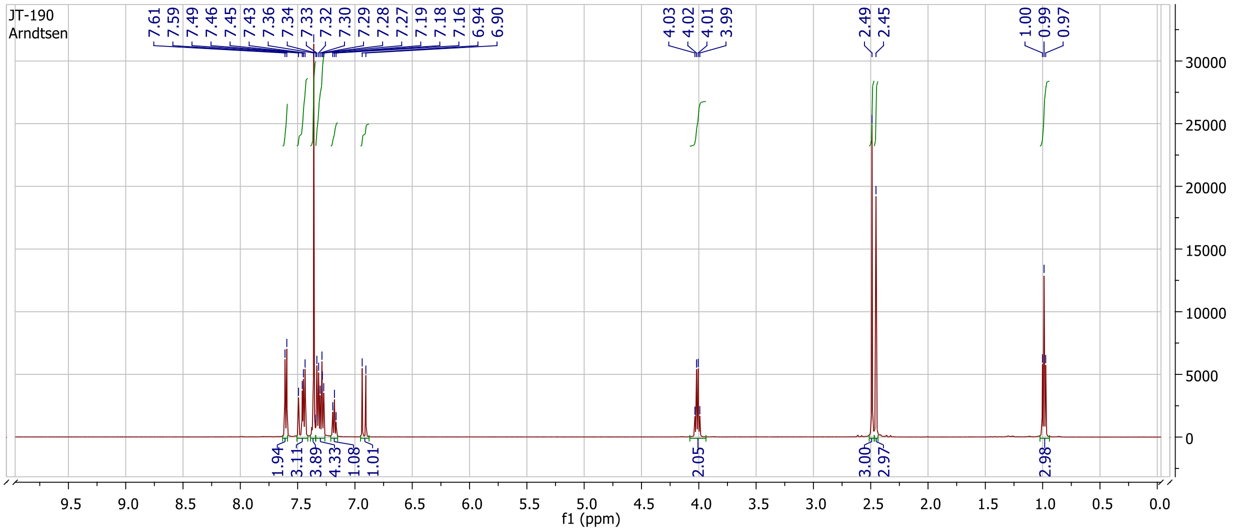

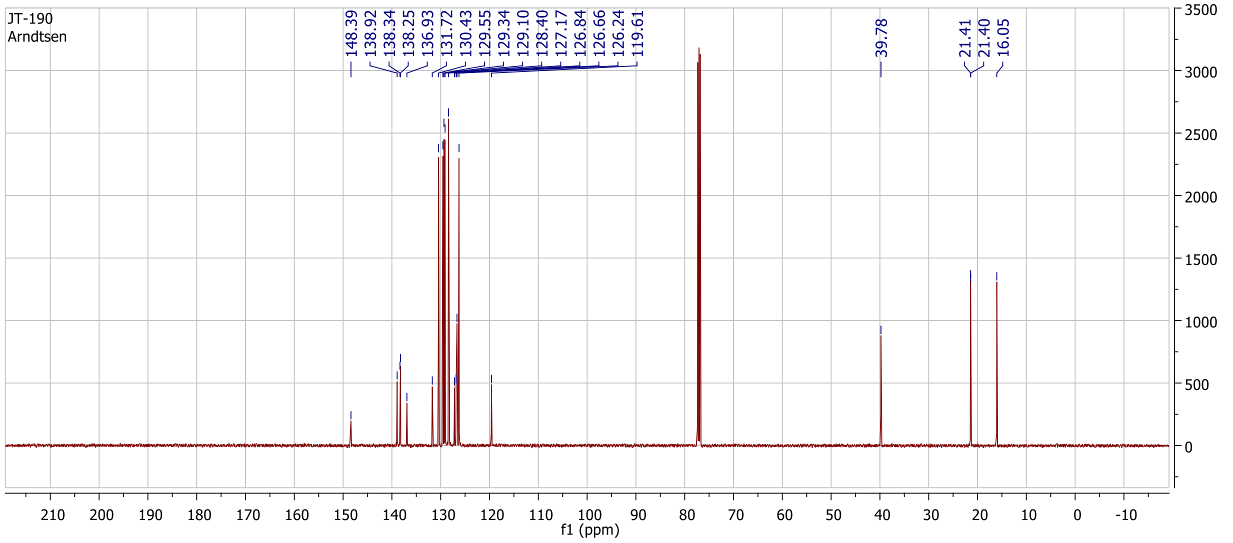

^{1}H and ^{13}C NMR spectra of 1aa

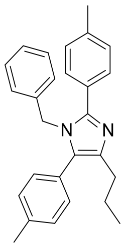

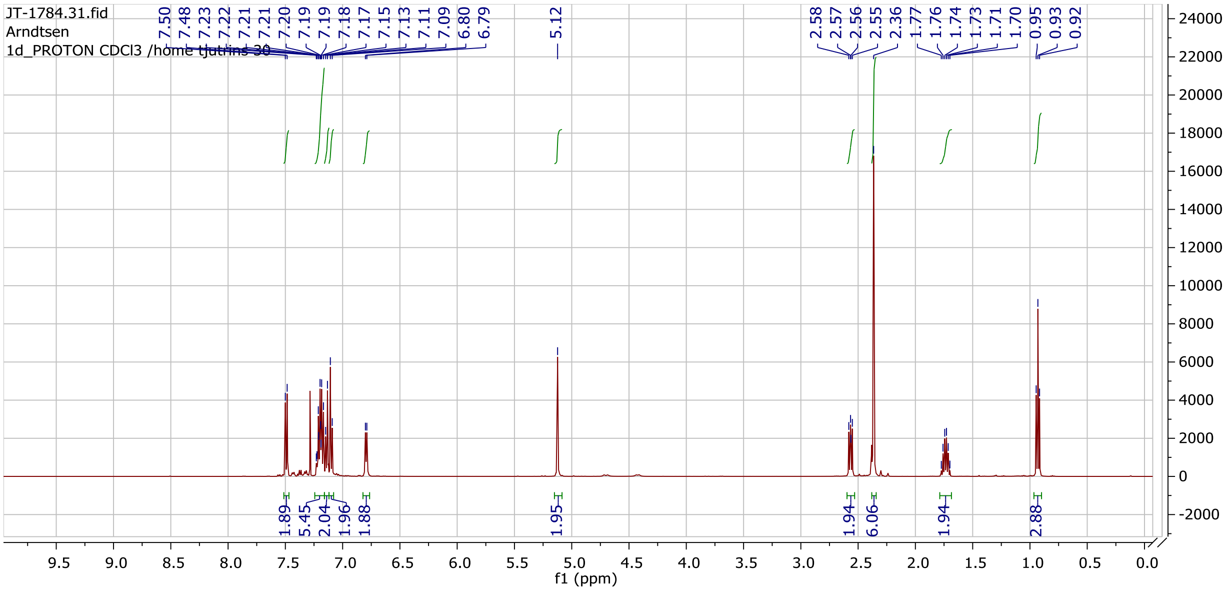

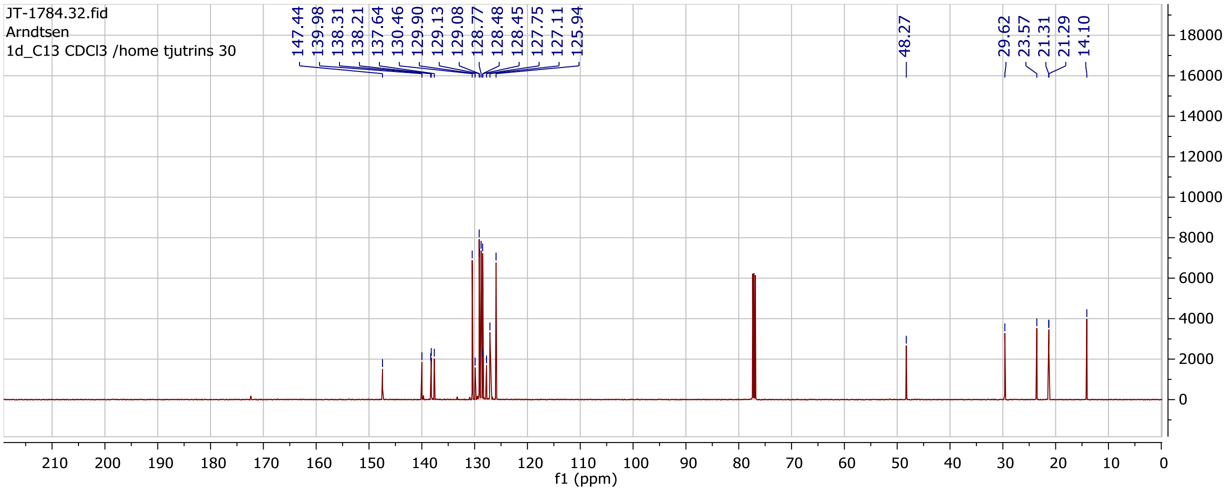

^{1}H and ^{13}C NMR spectra of 2a

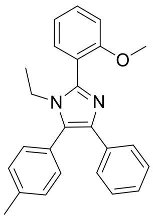

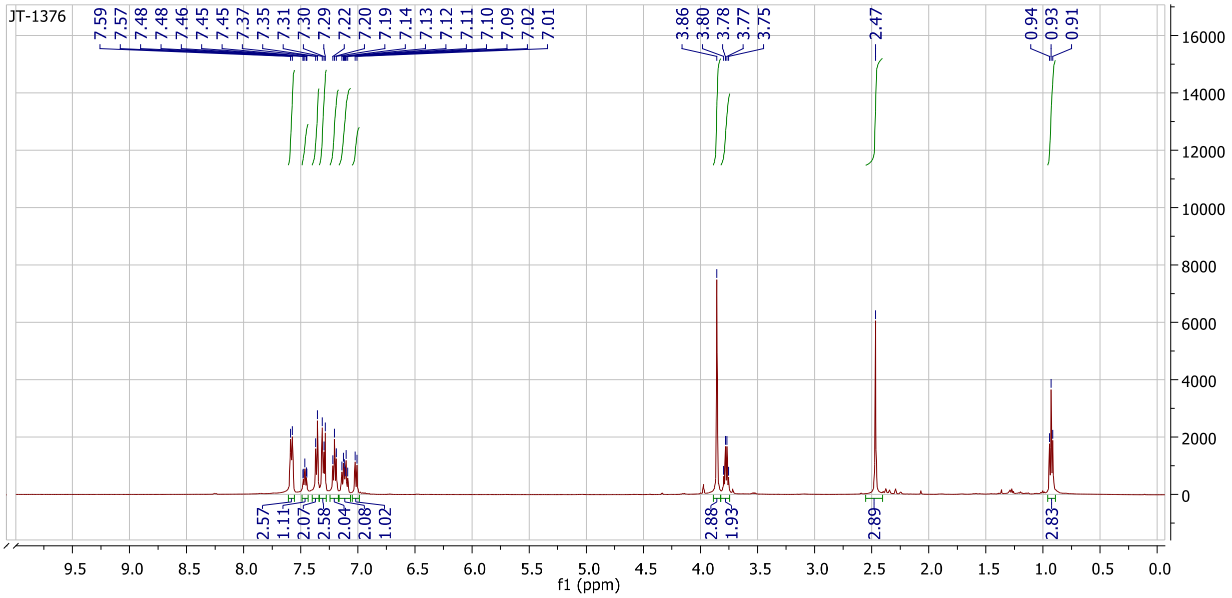

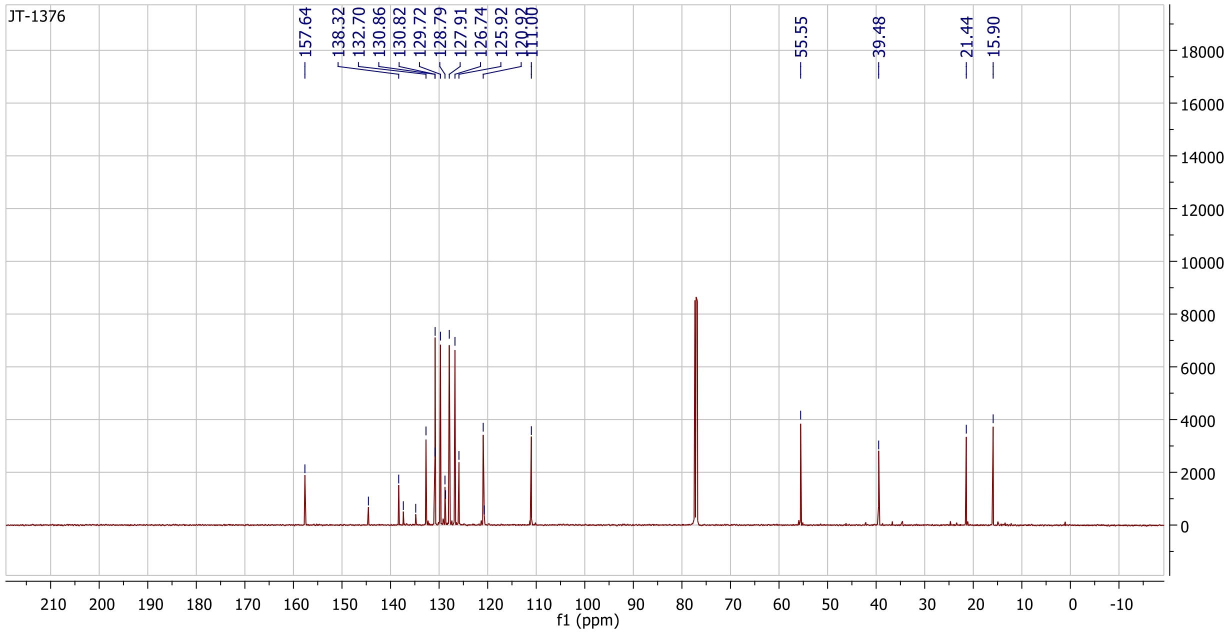

^{1}H and ^{13}C NMR spectra of 2b

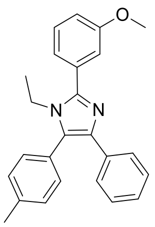

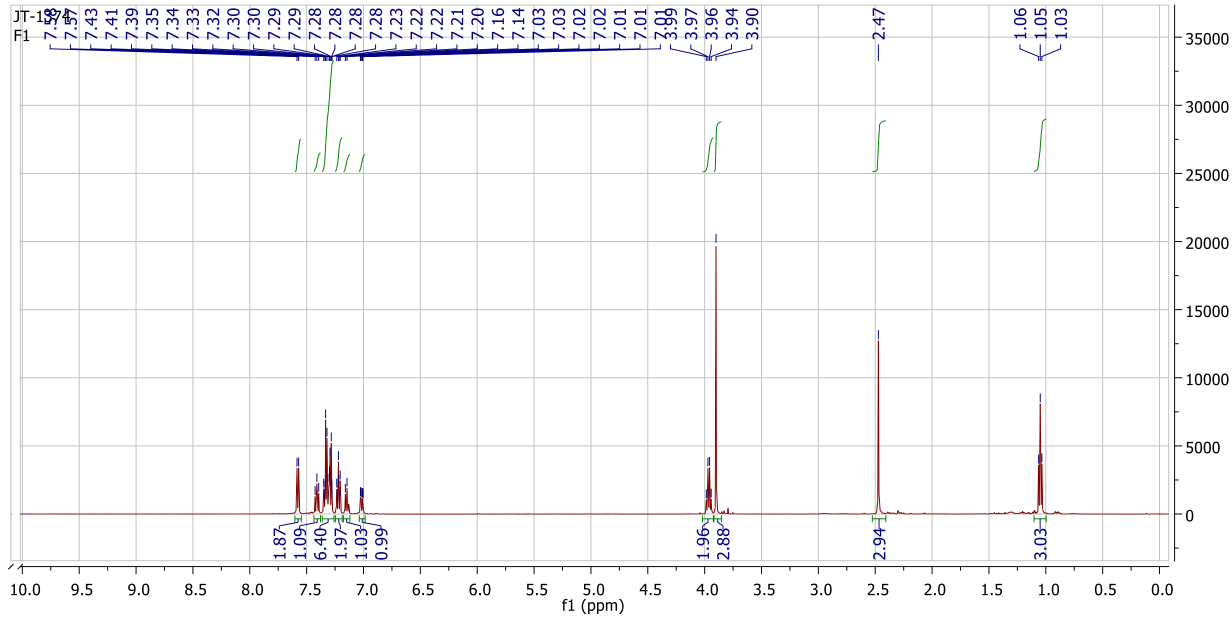

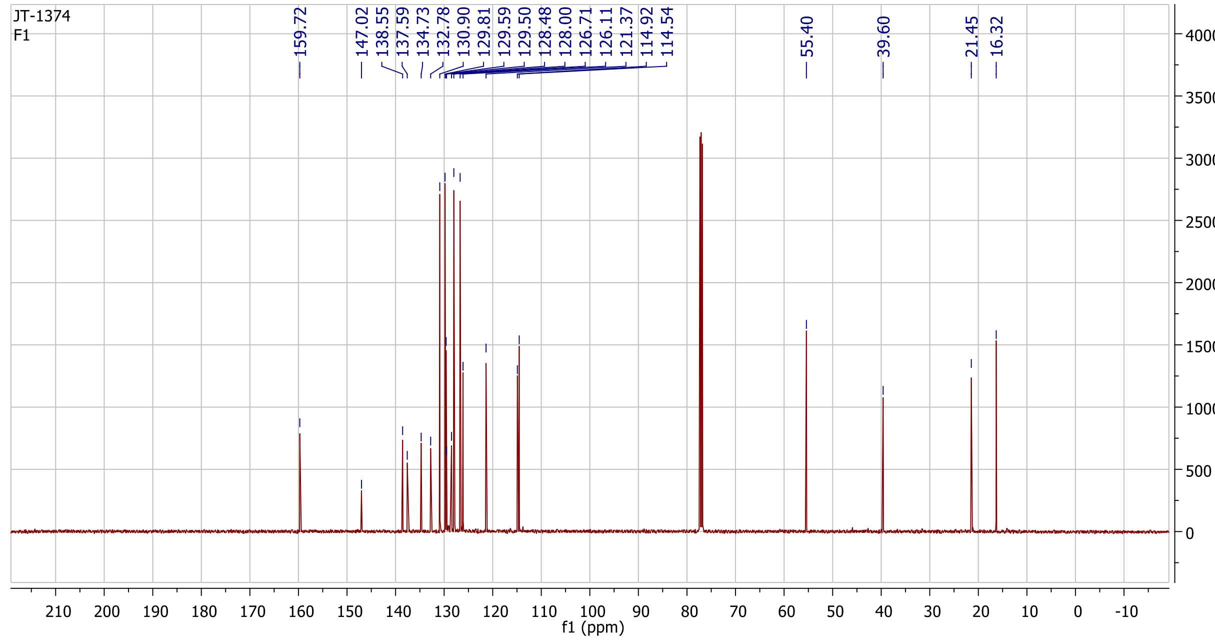

^{1}H and ^{13}C NMR spectra of 2c

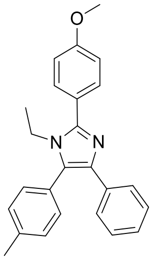

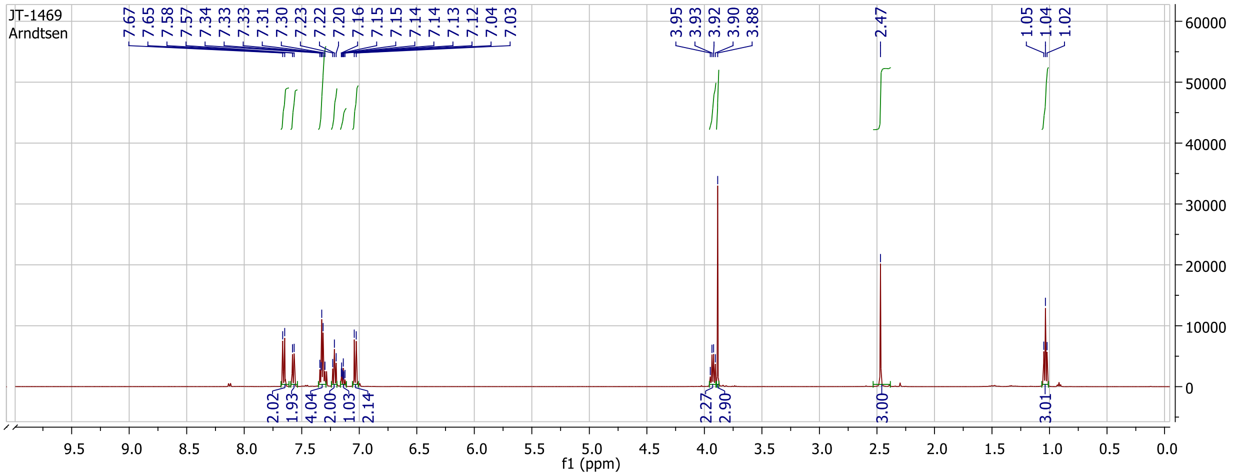

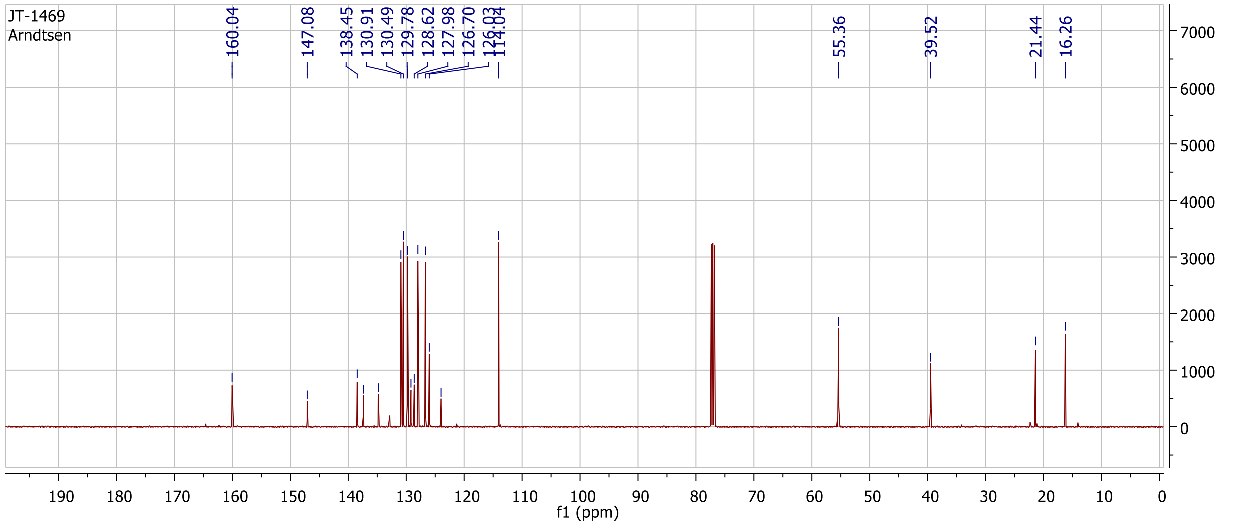

^{1}H and ^{13}C NMR spectra of 2d

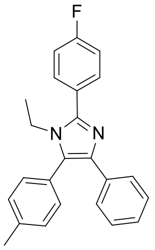

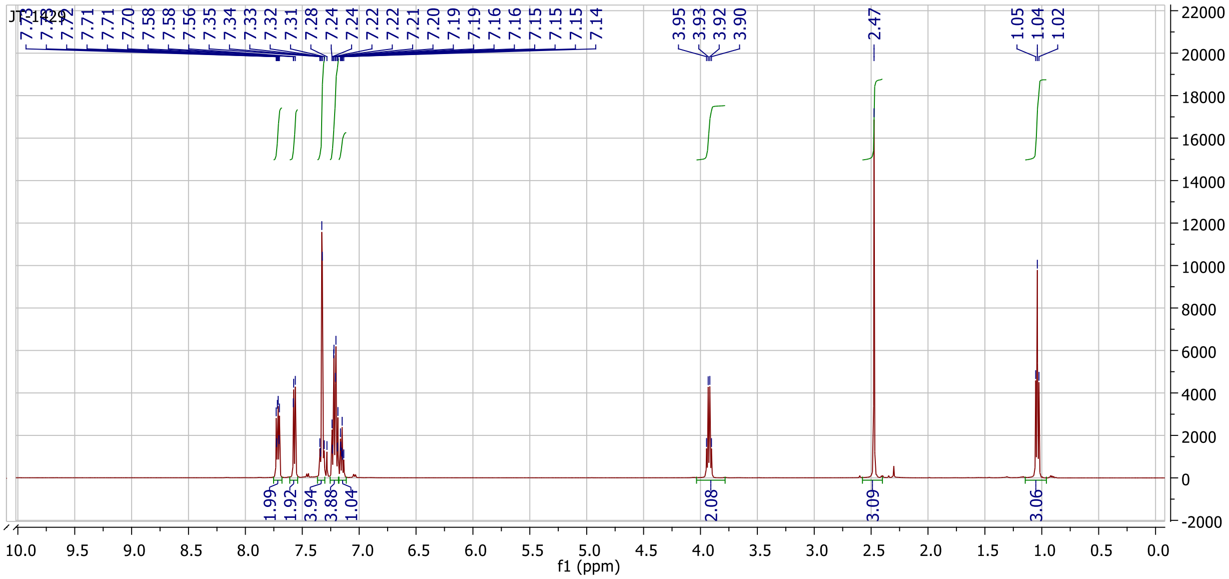

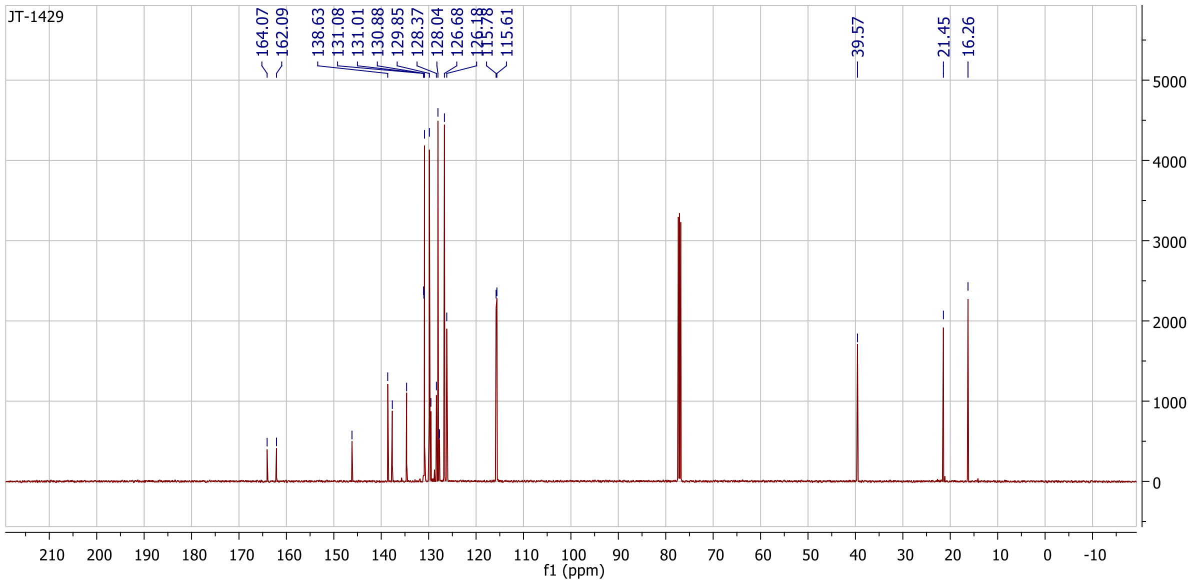

^{1}H and ^{13}C NMR spectra of 2e

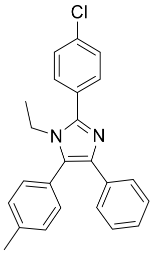

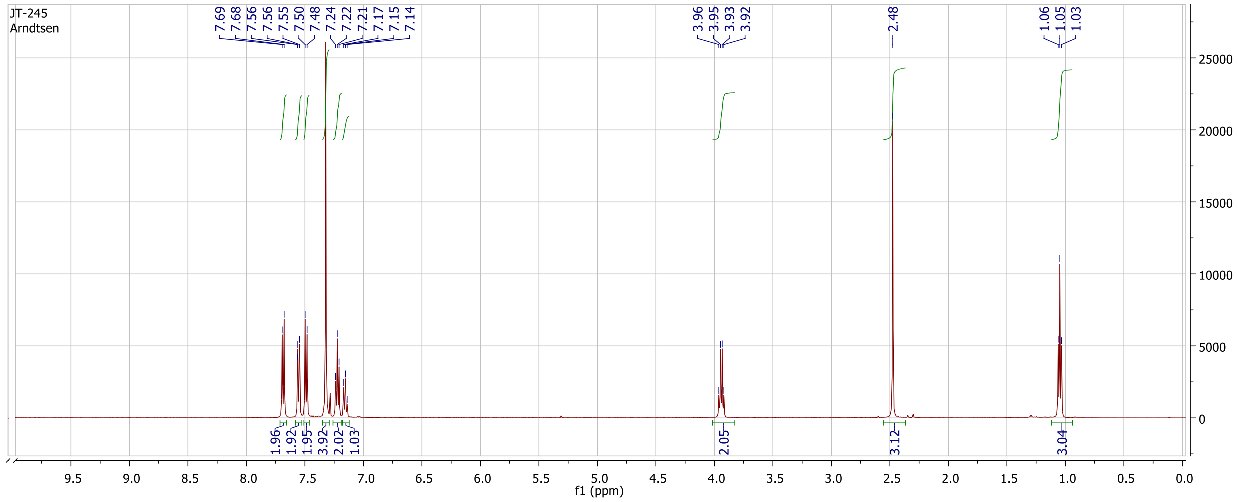

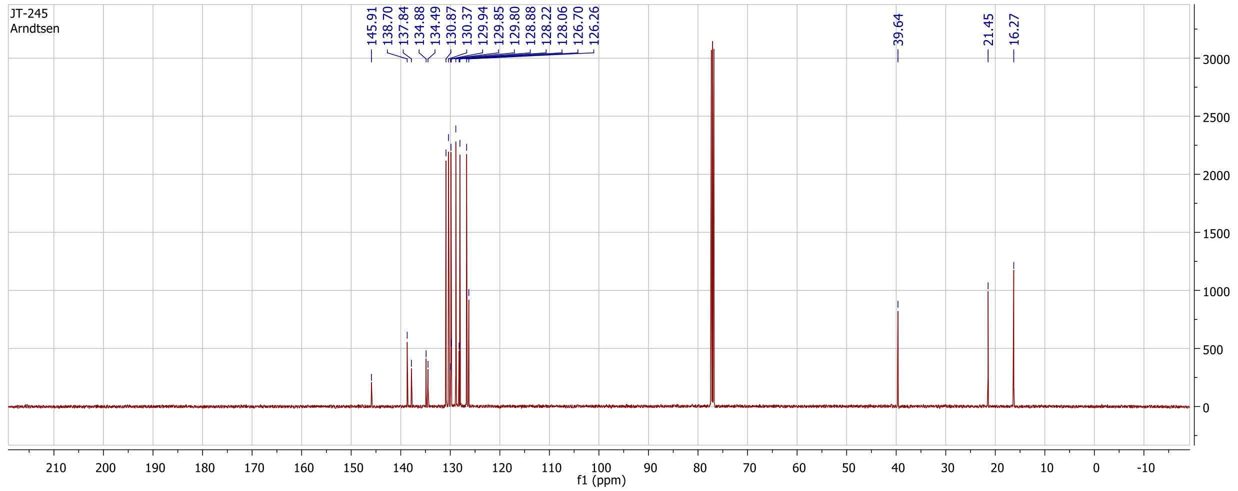

^{1}H and ^{13}C NMR spectra of 2f

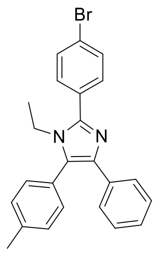

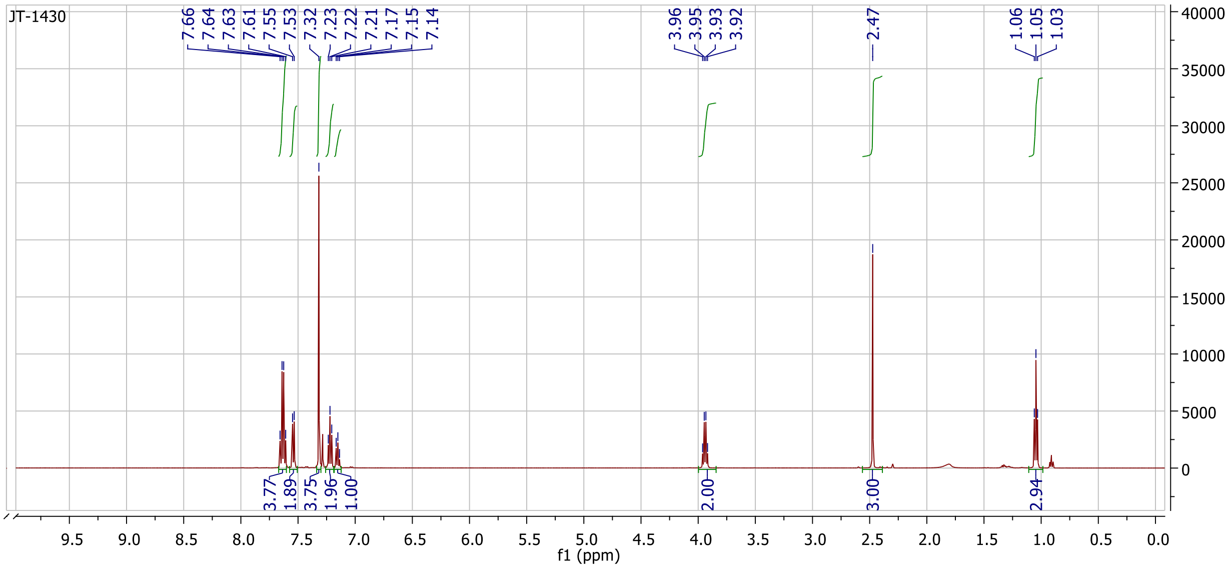

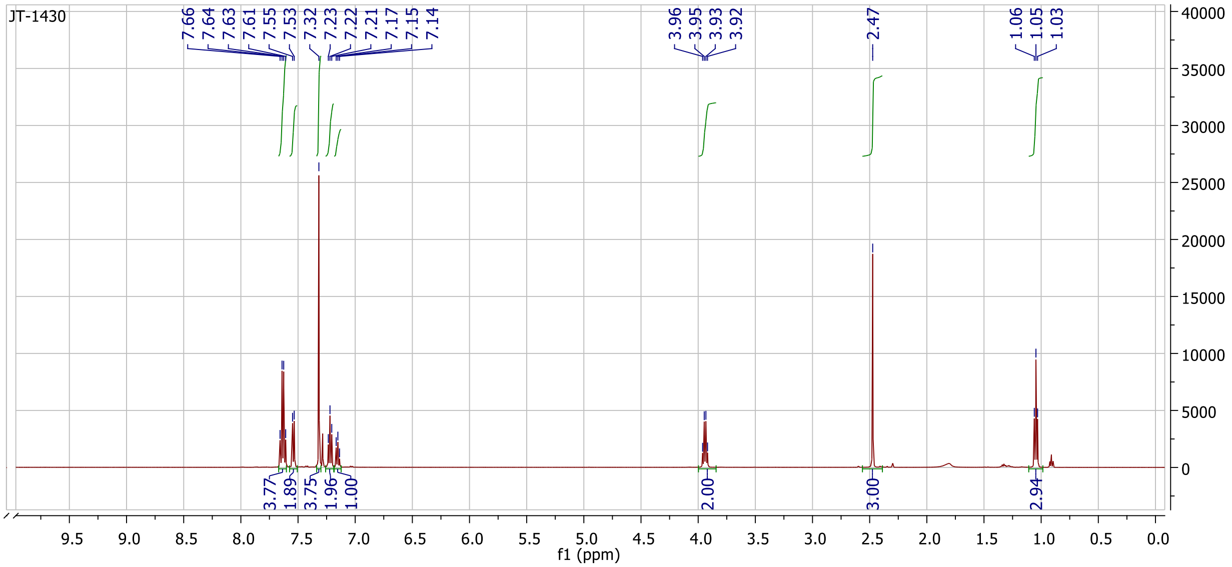

^{1}H and ^{13}C NMR spectra of 2g

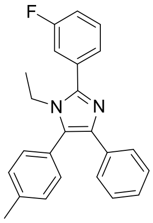

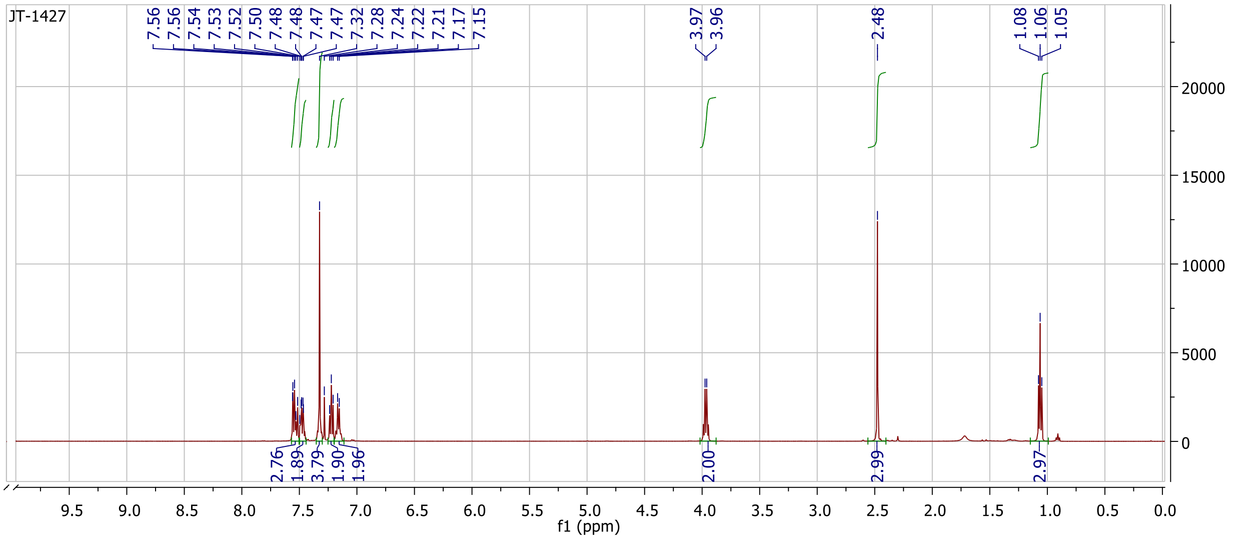

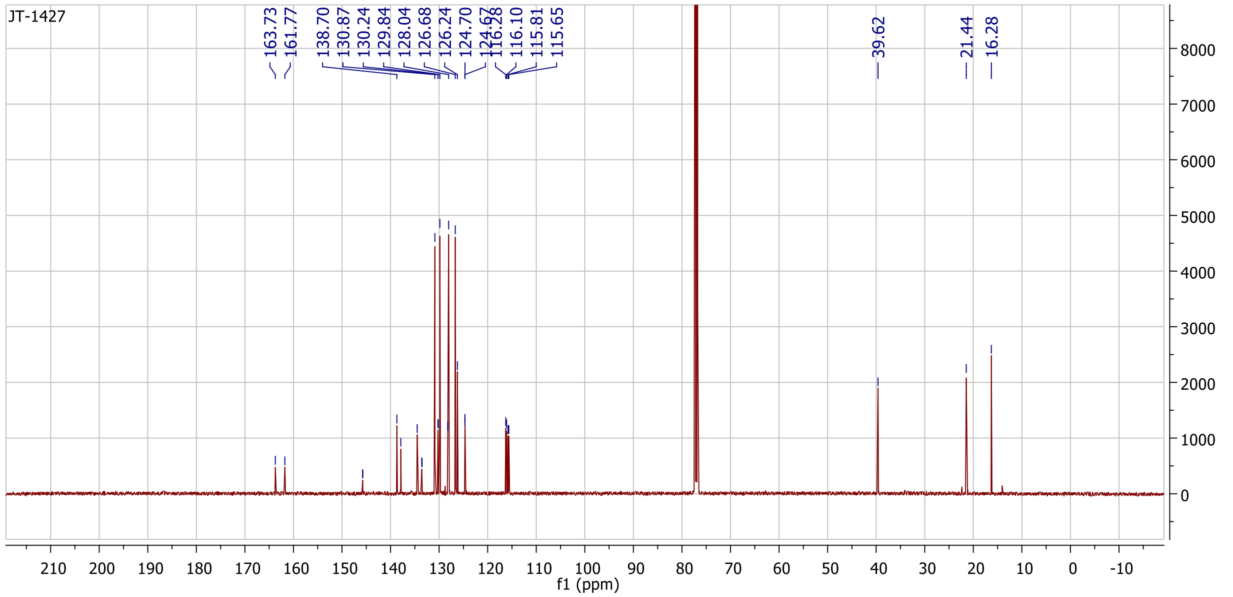

^{1}H and ^{13}C NMR spectra of 2h

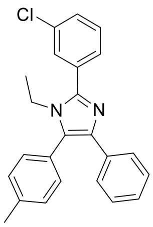

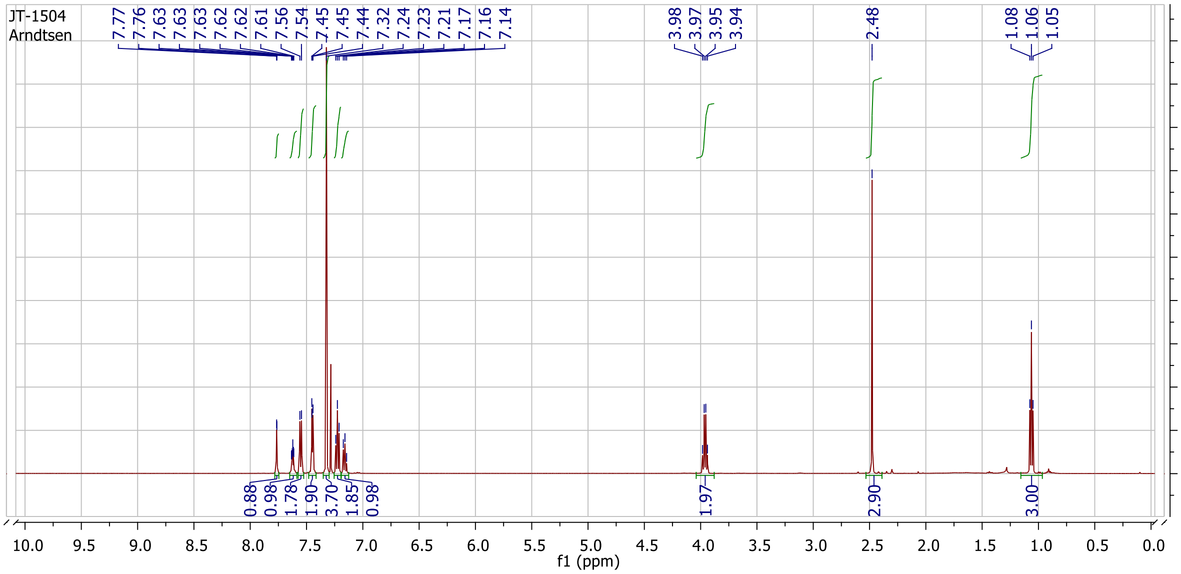

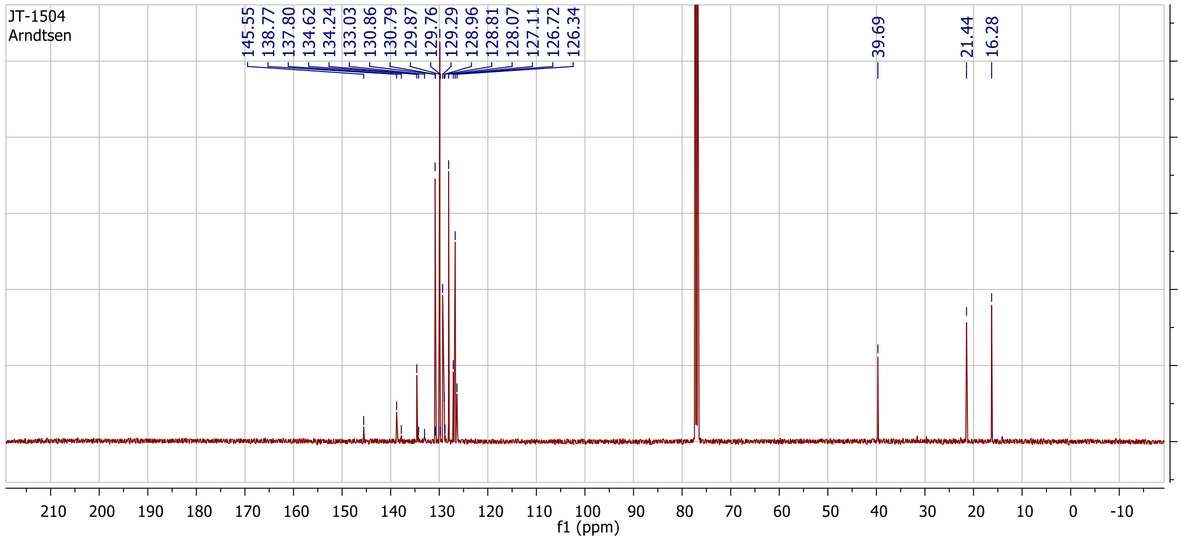

^{1}H and ^{13}C NMR spectra of 2i

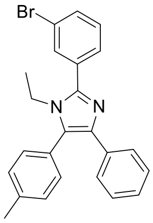

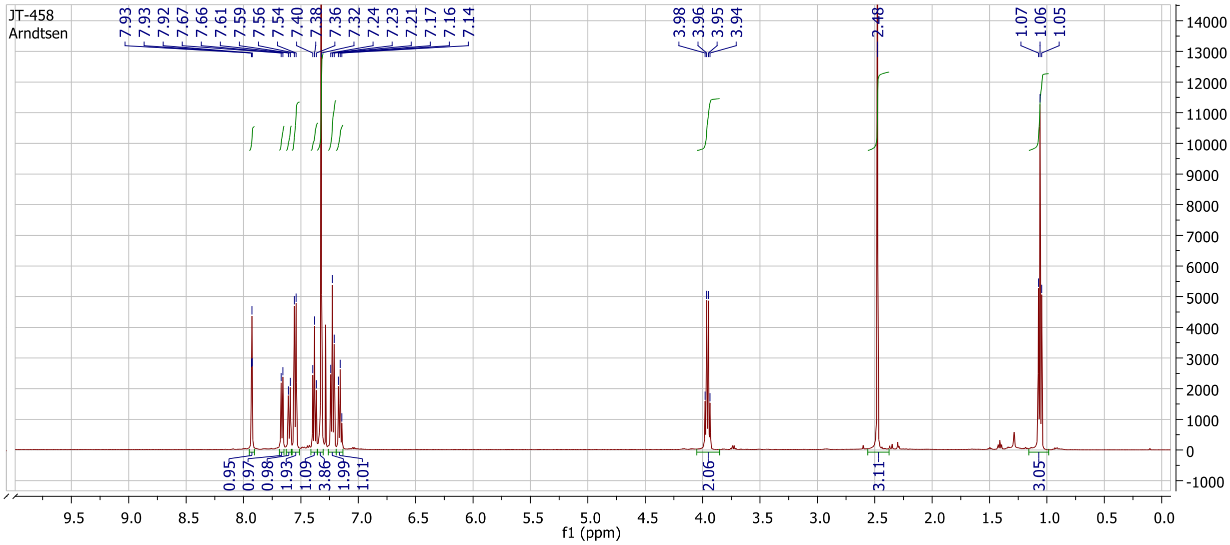

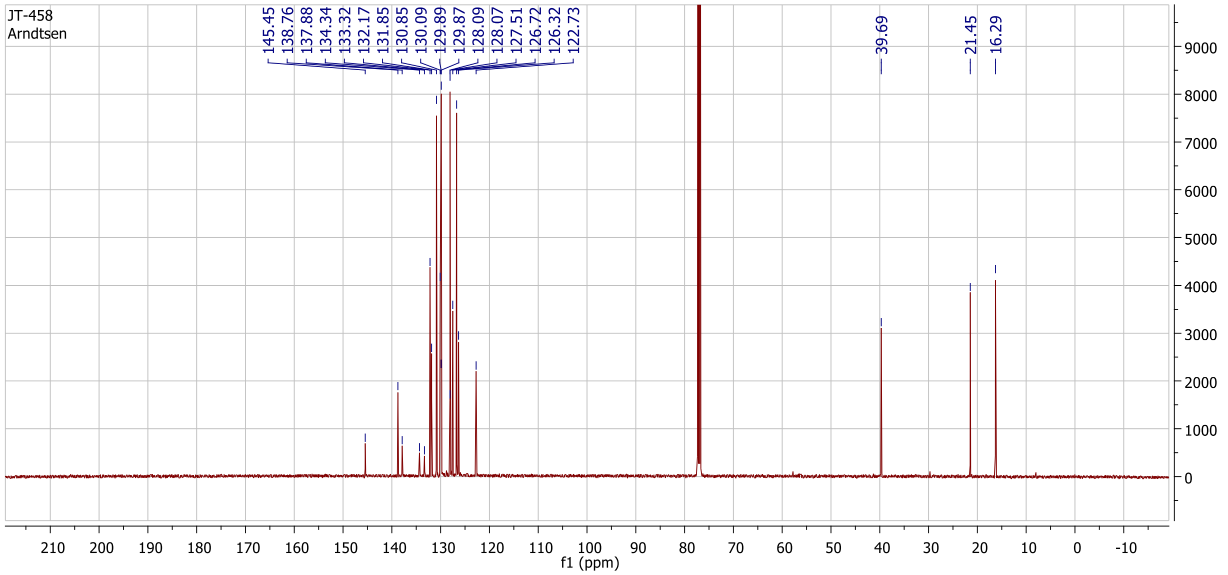

^{1}H and ^{13}C NMR spectra of 2j

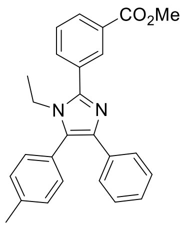

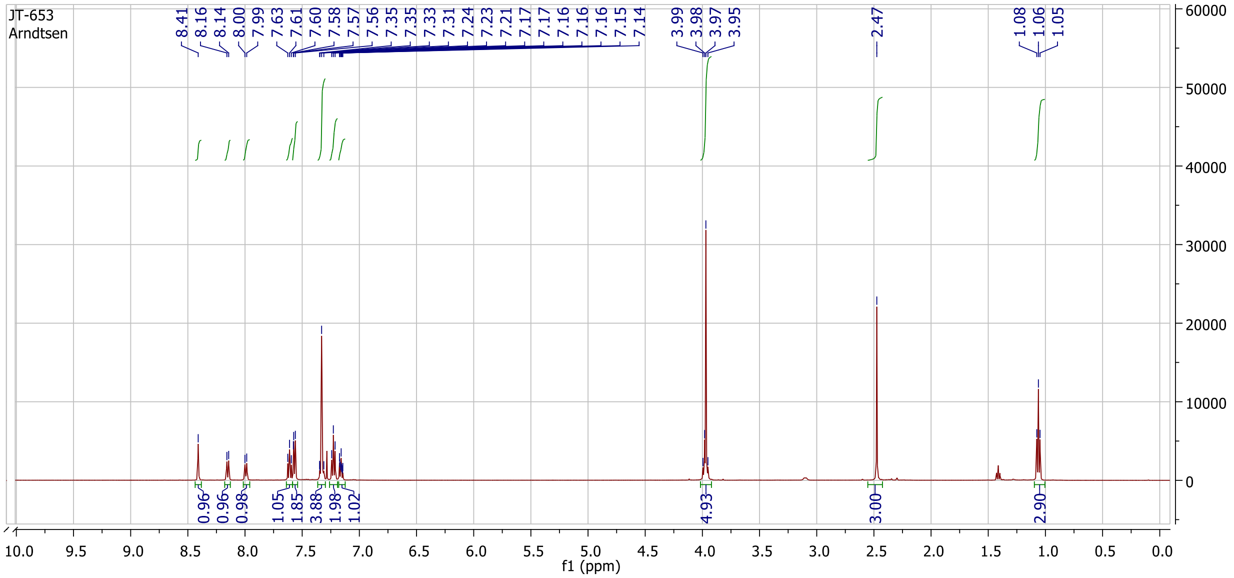

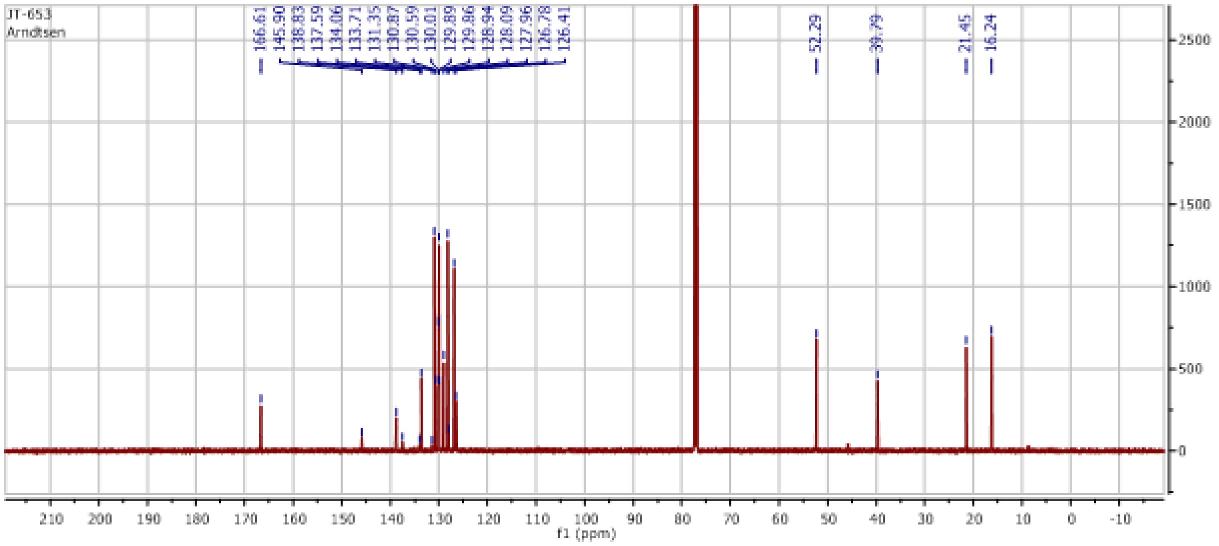

^{1}H and ^{13}C NMR spectra of 2k

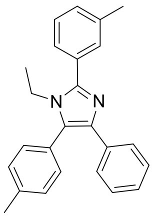

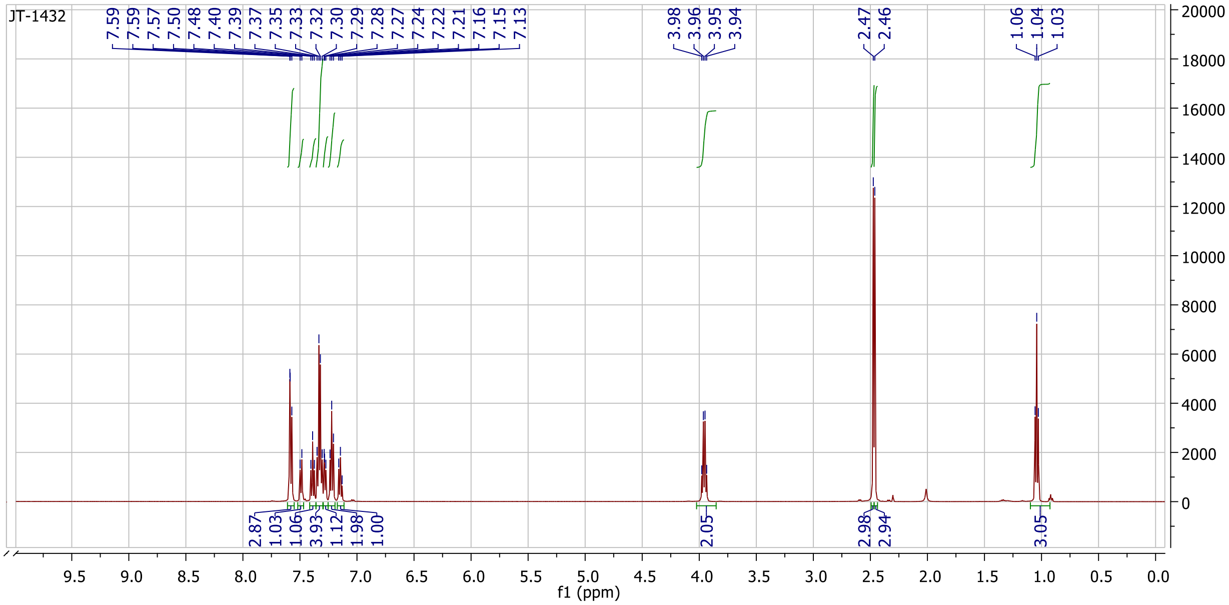

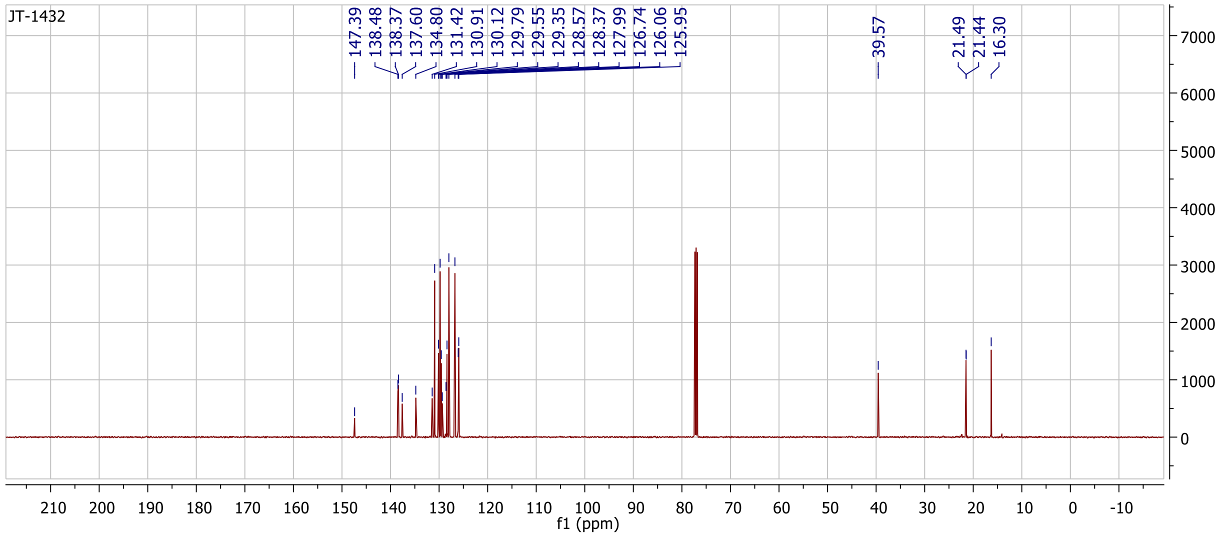

^{1}H and ^{13}C NMR spectra of 2l

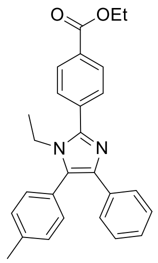

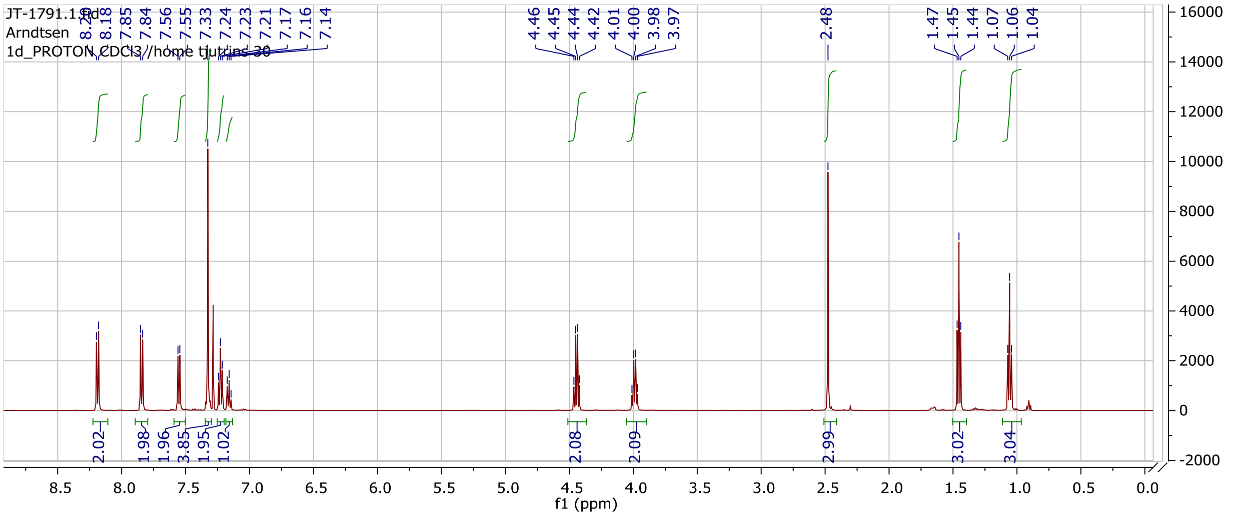

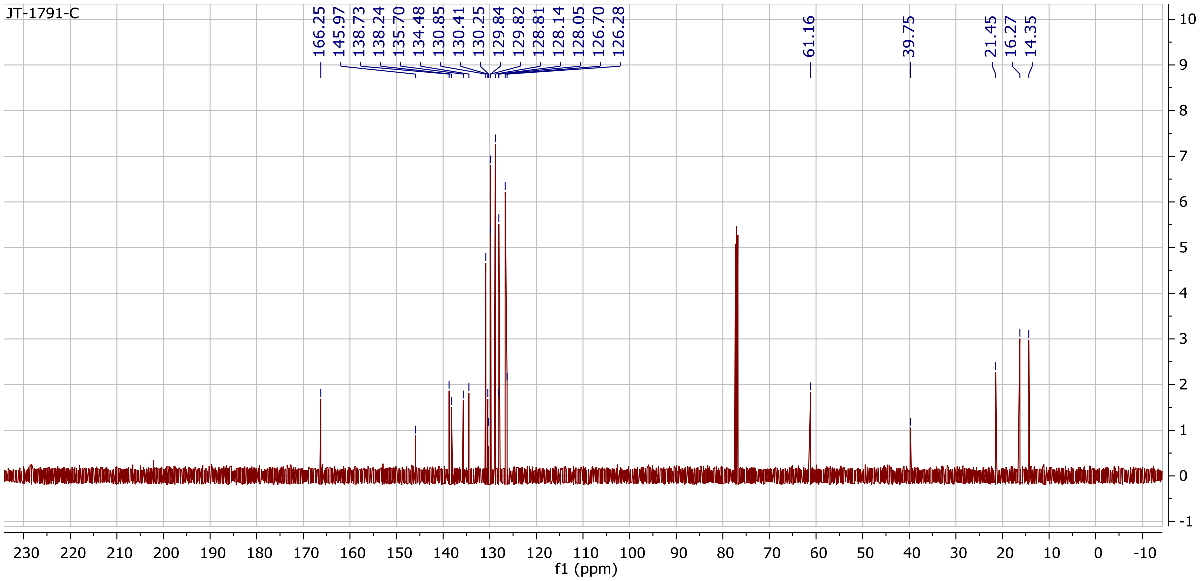

^{1}H and ^{13}C NMR spectra of 2m

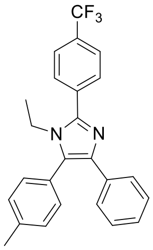

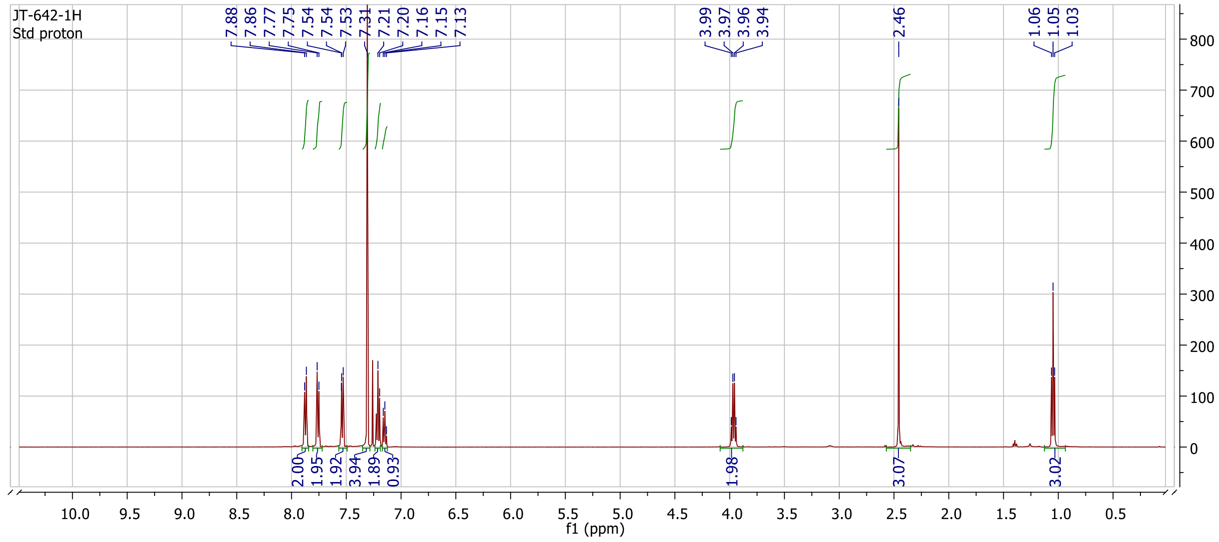

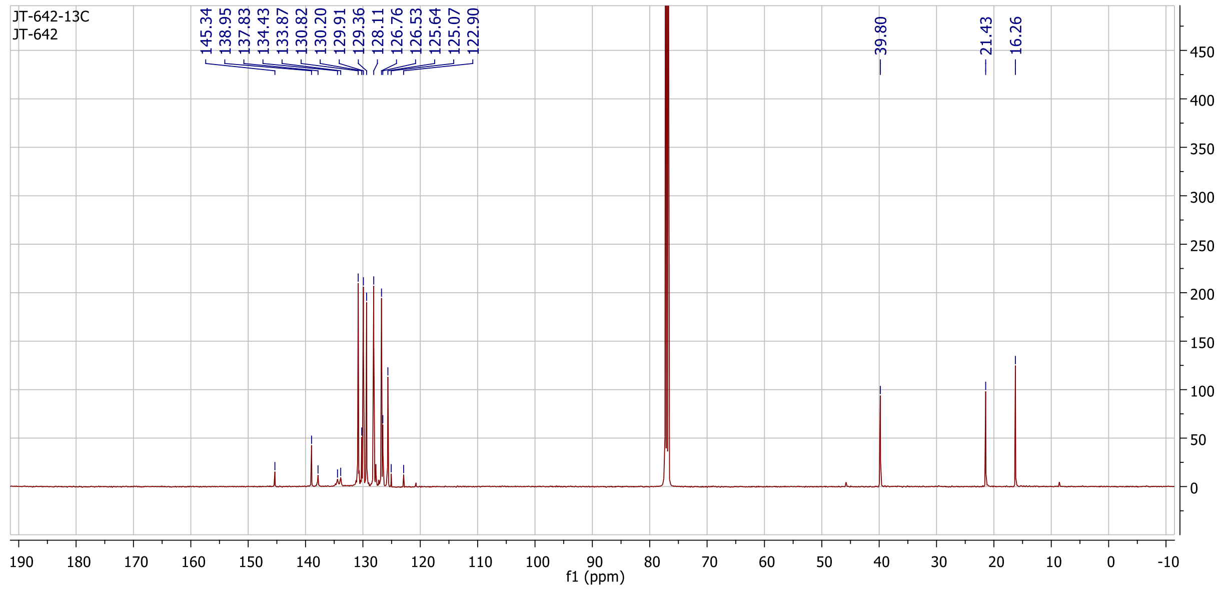

^{1}H and ^{13}C NMR spectra of 2n

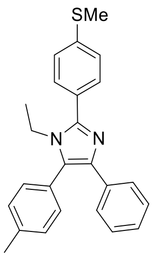

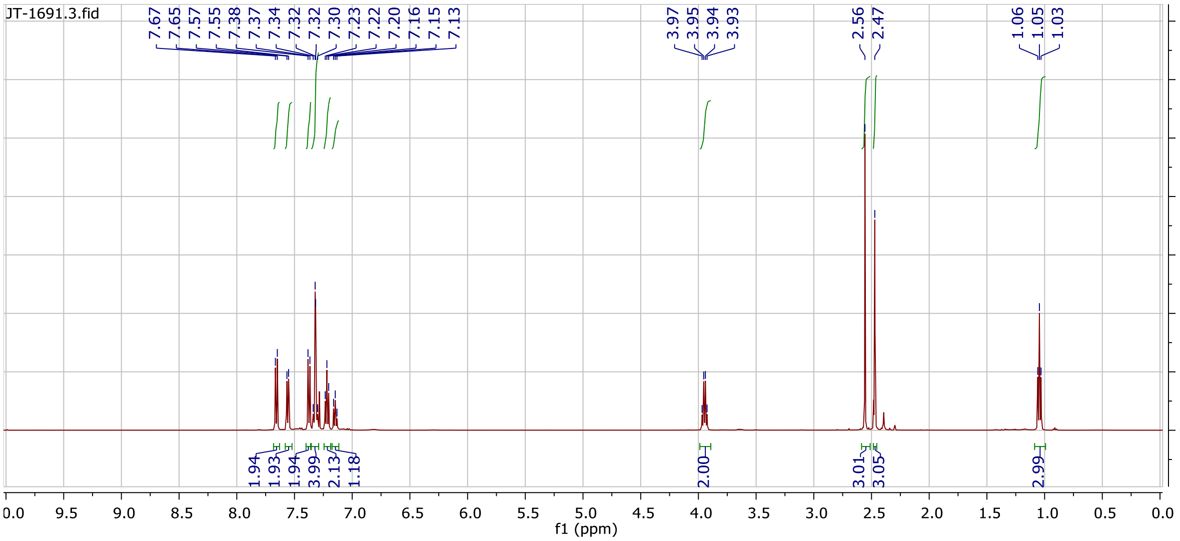

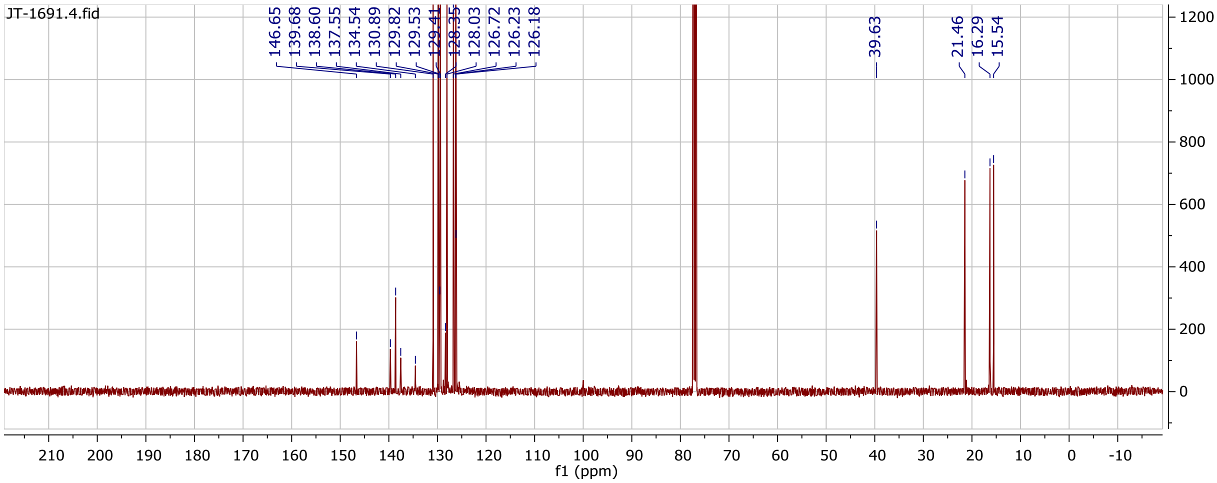

^{1}H and ^{13}C NMR spectra of 2o

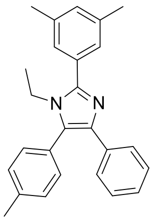

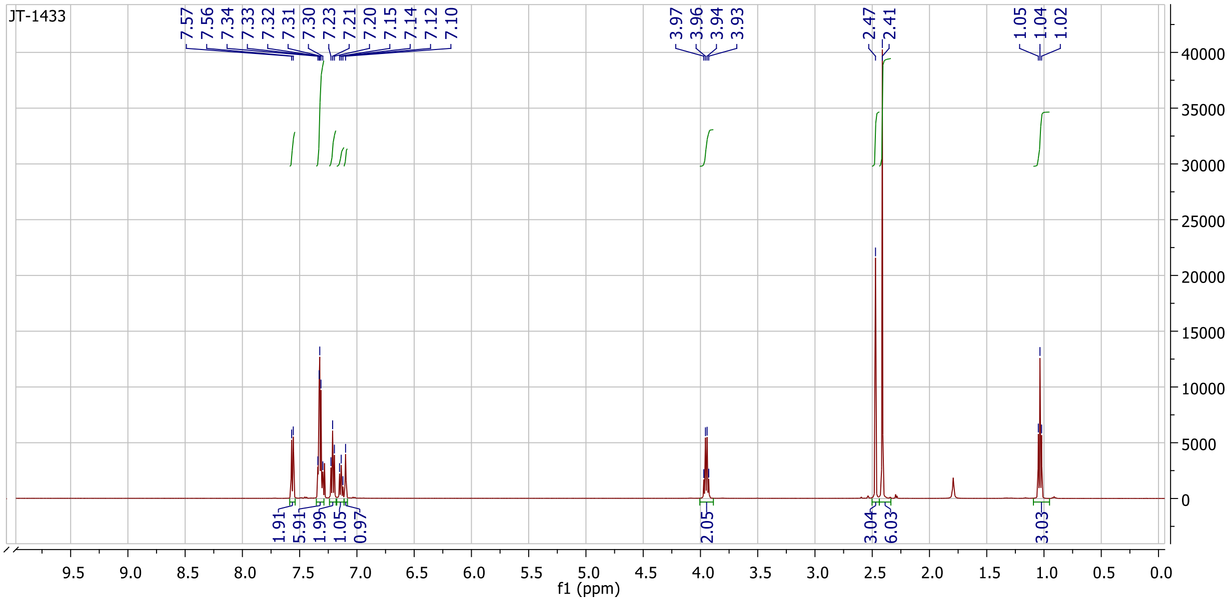

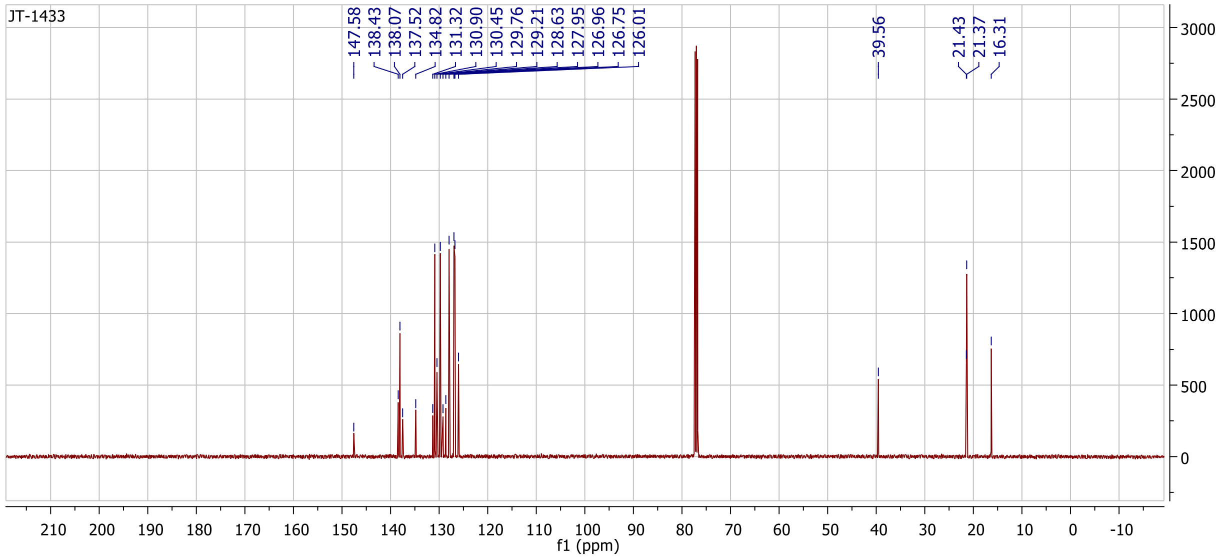

^{1}H and ^{13}C NMR spectra of 2p

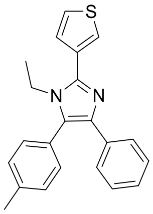

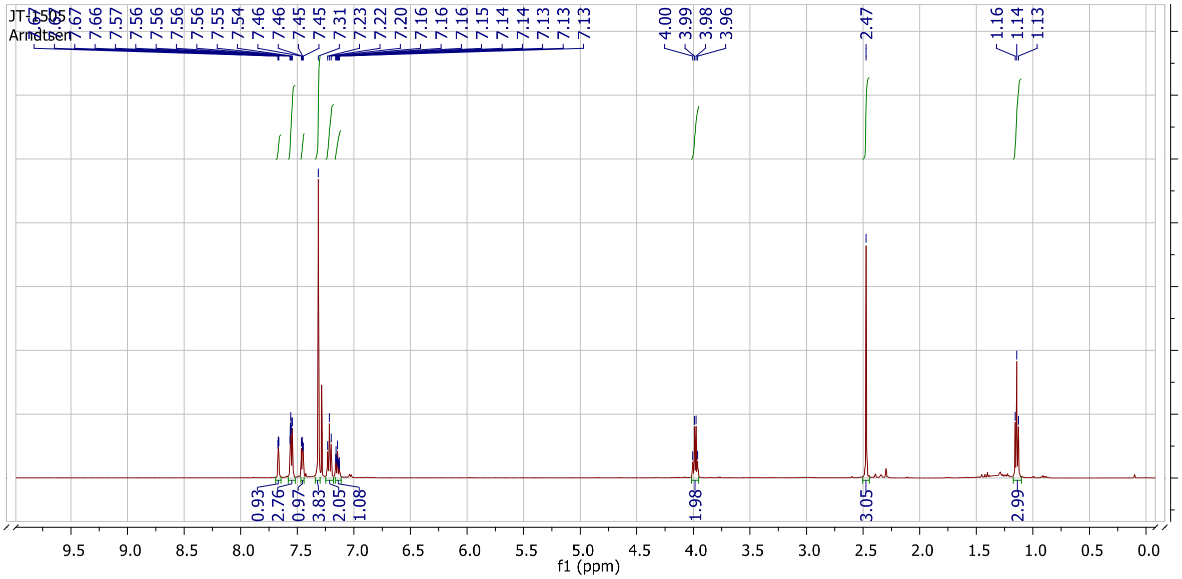

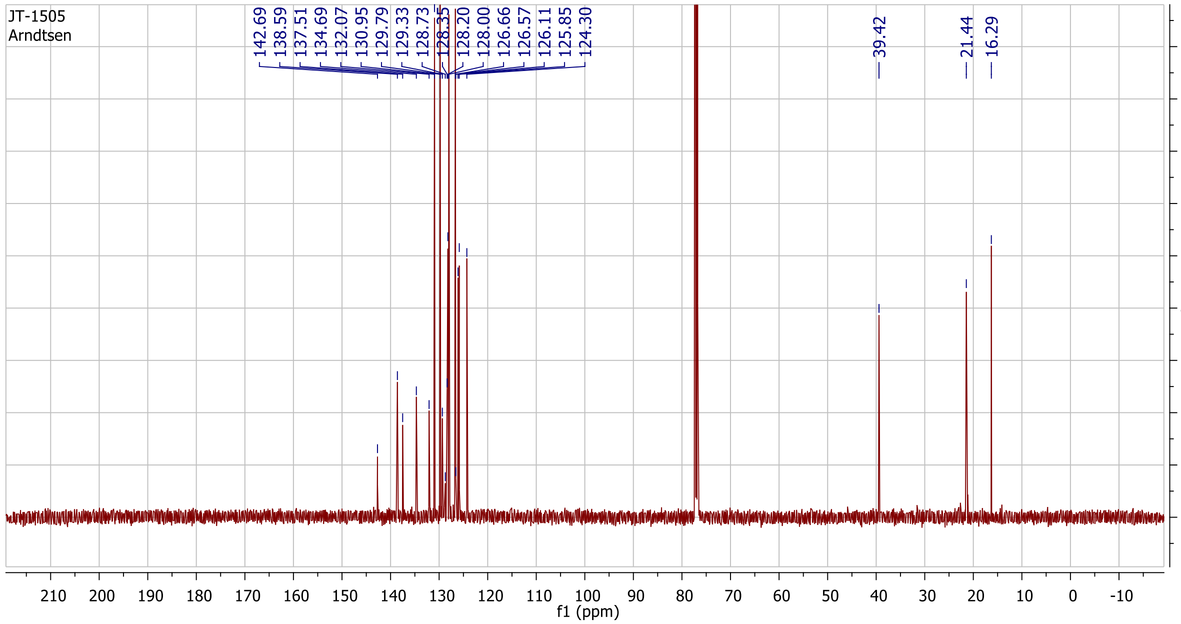

^{1}H and ^{13}C NMR spectra of 2q

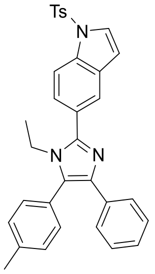

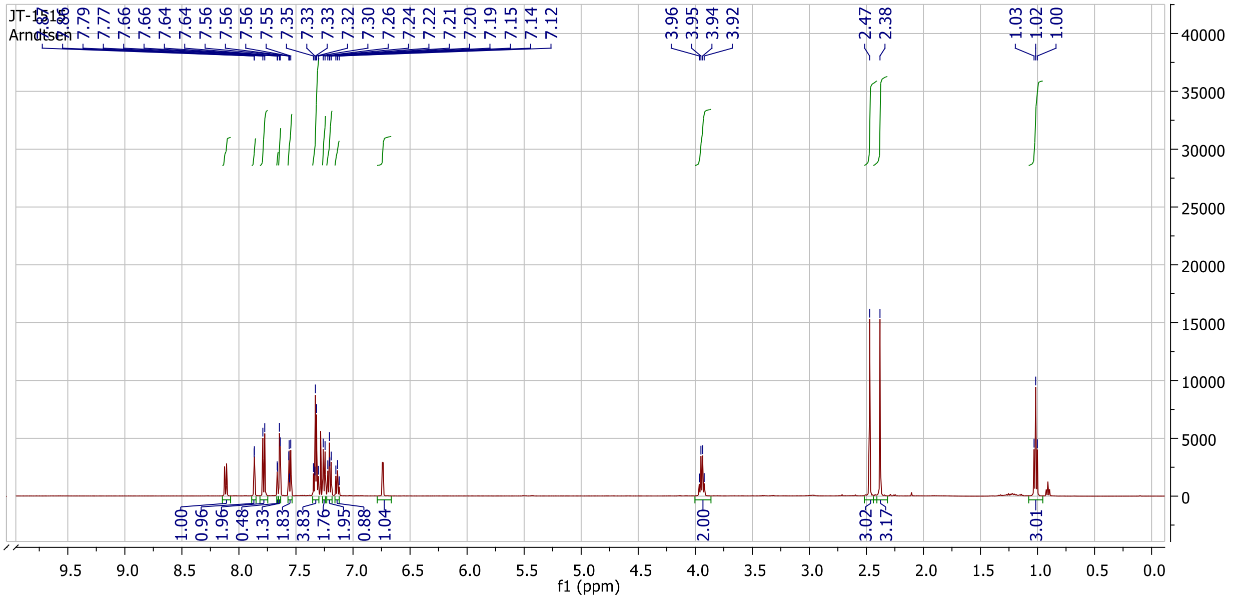

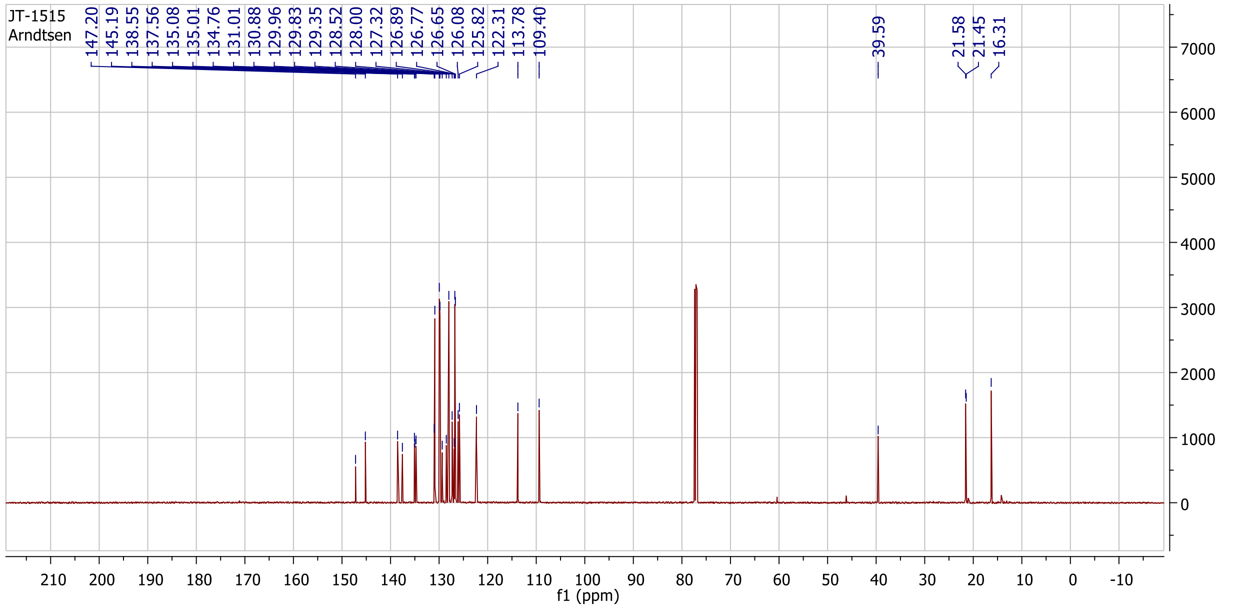

^{1}H and ^{13}C NMR spectra of 4-(1-benzyl-5-(6-methoxynaphthalen-2-yl)-2-(4-(methylthio) phenyl)-1H-imidazol-4-yl)pyridine

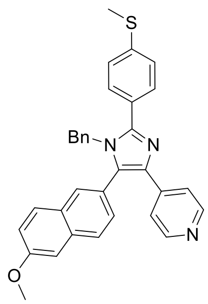

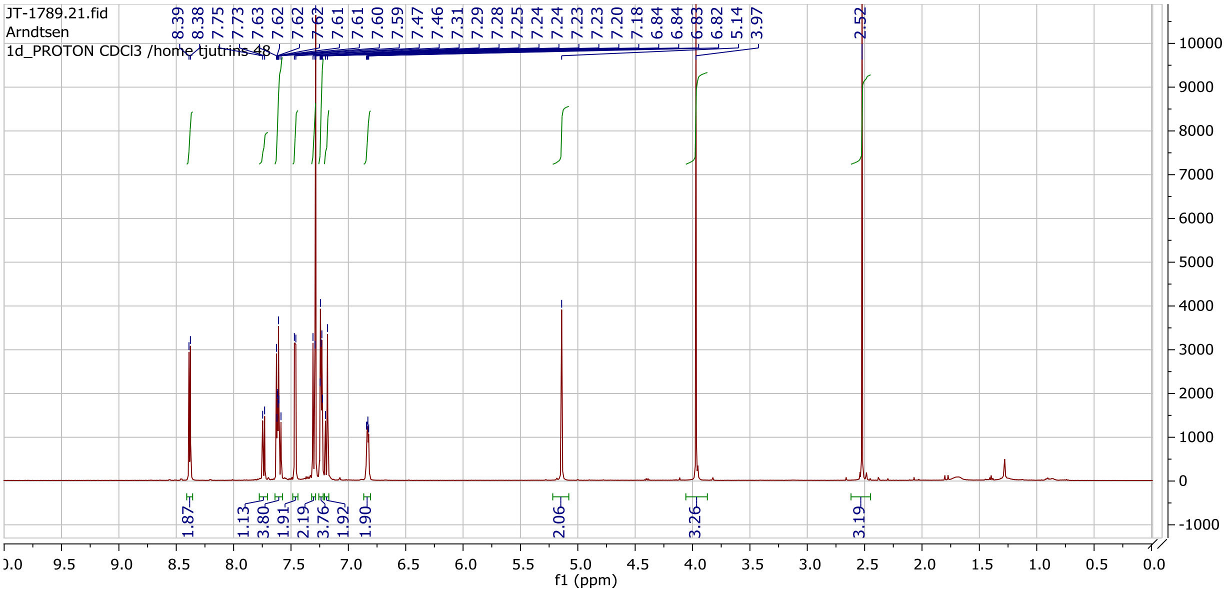

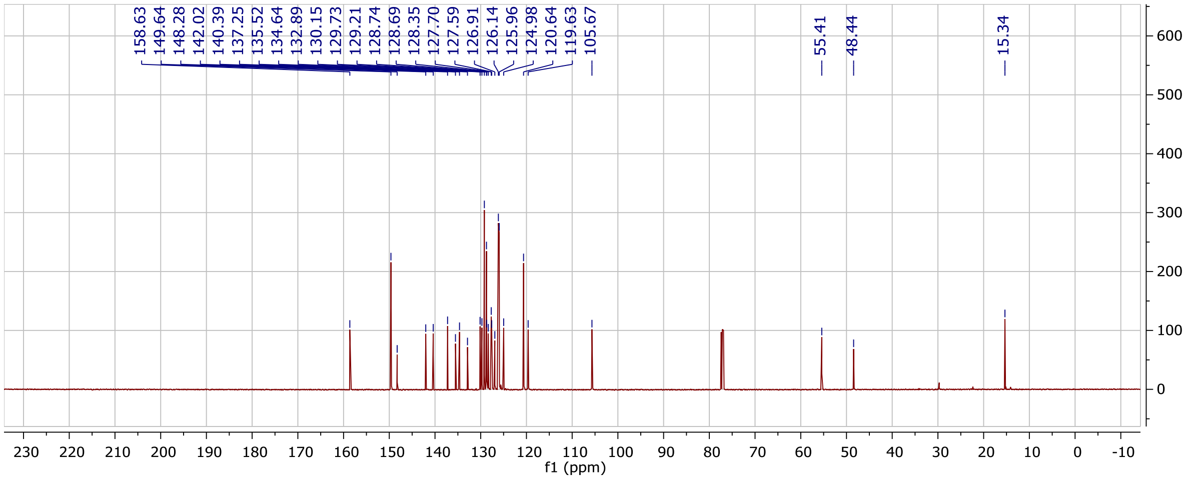

^{1}H and ^{13}C NMR spectra of 4-(1-benzyl-5-(6-methoxynaphthalen-2-yl)-2-(4-(methylthio) phenyl)-1H-imidazol-4-yl)pyridine 6

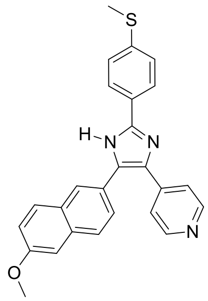

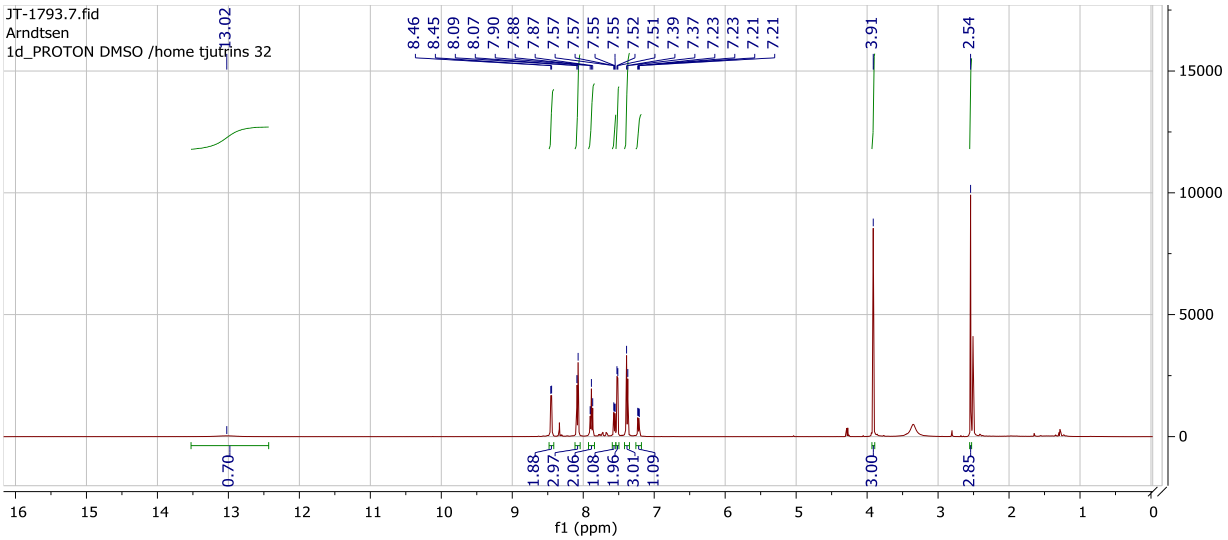

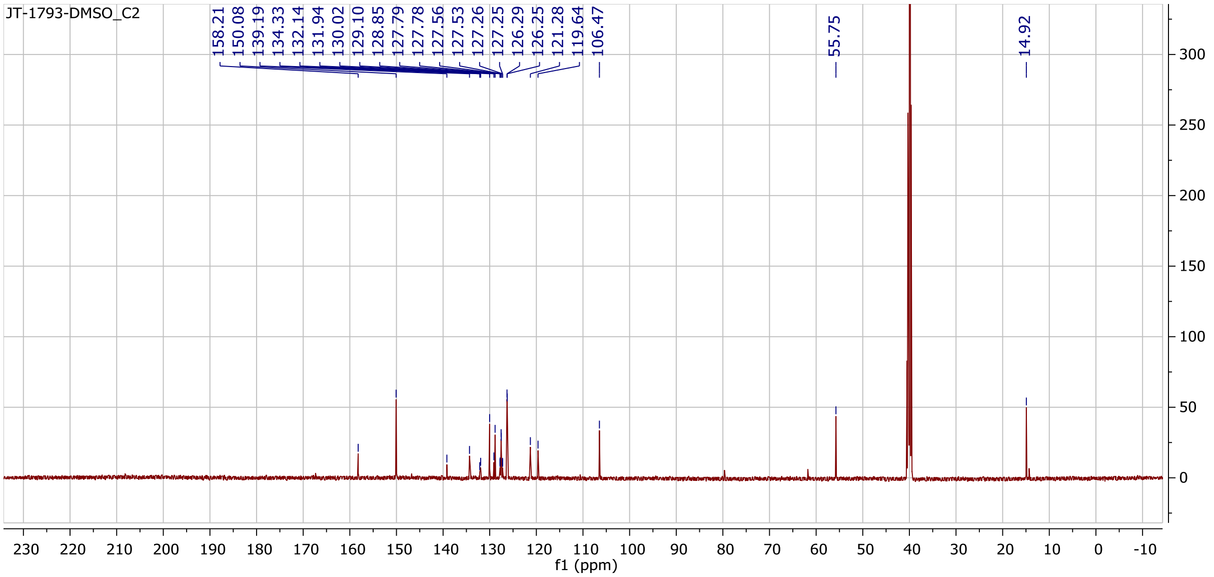

Supplement: Supplementary file 1 [file SC-008-C6SC04371B-s001.pdf]
